# Scandinavian guidelines for initial management of minor and moderate head trauma in children

**DOI:** 10.1186/s12916-016-0574-x

**Published:** 2016-02-18

**Authors:** Ramona Astrand, Christina Rosenlund, Johan Undén, for the Scandinavian Neurotrauma Committee (SNC)

**Affiliations:** Department of Neurosurgery, Neurocenter 2091, Rigshospitalet, Blegdamsvej 9, 2100 Copenhagen, Denmark; Department of Neurosurgery, Odense University Hospital, Sdr. Boulevard 29, 5000 Odense C, Denmark; Department of Intensive Care and Perioperative Medicine, Institute for Clinical Sciences, Skåne University Hospital, Södra Förstadsgatan 101, 20502 Malmö, Sweden

**Keywords:** Guidelines, Prediction rule, Children, Mild, moderate, head trauma, Brain injury, Computed tomography, Evidence-based, GRADE

## Abstract

**Background:**

The management of minor and moderate head trauma in children differs widely between countries. Presently, there are no existing guidelines for management of these children in Scandinavia. The purpose of this study was to produce new evidence-based guidelines for the initial management of head trauma in the paediatric population in Scandinavia. The primary aim was to detect all children in need of neurosurgical intervention. Detection of any traumatic intracranial injury on CT scan was an important secondary aim.

**Methods:**

General methodology according to the Appraisal of Guidelines for Research and Evaluation (AGREE) II and the Grading of Recommendations Assessment, Development and Evaluation (GRADE) system was used. Systematic evidence-based review was performed according to the Preferred Reporting Items for Systematic Reviews and Meta-Analyses (PRISMA) methodology and based upon relevant clinical questions with respect to patient-important outcomes. Quality ratings of the included studies were performed using Quality Assessment of Diagnostic Accuracy Studies (QUADAS)-2 and Centre of Evidence Based Medicine (CEBM)-2 tools. Based upon the results, GRADE recommendations, a guideline, discharge instructions and in-hospital observation instructions were drafted. For elements with low evidence, a modified Delphi process was used for consensus, which included relevant clinical stakeholders.

**Results:**

The guidelines include criteria for selecting children for CT scans, in-hospital observation or early discharge, and suggestions for monitoring routines and discharge advice for children and guardians. The guidelines separate mild head trauma patients into high-, medium- and low-risk categories, favouring observation for mild, low-risk patients as an attempt to reduce CT scans in children.

**Conclusions:**

We present new evidence and consensus based Scandinavian Neurotrauma Committee guidelines for initial management of minor and moderate head trauma in children. These guidelines should be validated before extensive clinical use and updated within four years due to rapid development of new diagnostic tools within paediatric neurotrauma.

**Electronic supplementary material:**

The online version of this article (doi:10.1186/s12916-016-0574-x) contains supplementary material, which is available to authorized users.

## Background

Head trauma is a common reason for an emergency department (ED) visit, especially among adolescents and adults [1]. The incidence of head trauma in the paediatric population is estimated to 180 - 300 per 100,000 [2, 3]. About 80-90 % of these injuries are classed as minor head traumas (MHT), which includes both minimal and mild head trauma, whilst approximately 10 % have moderate to severe head trauma (Glasgow Coma Scale [GCS] score 3-8) [1, 4]. According to the Head Injury Severity Scale (HISS) classification [5], mild head injury patients are initially conscious at first assessment (GCS score 14-15), may have had a brief loss of consciousness (LOC) or amnesia, but do not have any focal neurological deficits on admission. Mortality and the need for neurosurgical interventions are rare in this patient group (0.1-0.2 %) [6, 7], and about 4-6 % have trauma related abnormality on the initial computed tomography (CT) scan [7–9]. Although serious complications after MHT in children are rare, intracranial lesions, such as epidural haematomas, can have major consequences and be potentially life-threatening if left untreated. Immediate CT scanning and in-hospital clinical observation are considered equally good strategies in triaging patients after MHT with respect to intracranial complications and medical outcome [10], although CT scanning and early discharge are economically more advantageous [11]. Due to the large number of head trauma patients and the low number of intracranial complications, CT scanning is both a public health issue as well as an economic dilemma.

During the last decades, CT use has rapidly increased. In the USA, more than half of the children seen in the ED for MHT will receive a head CT [12]. In 2012, the Nordic Radiation Protection Authorities published a joint statement concerning the increased use of CT in the Nordic countries, advocating for increased awareness of radiation risks and urging that CT scans only be done when clinically justified [13]. A previous study from Sweden has also showed that CT of the head is the most common CT investigation (50 % of all CTs performed) which was especially true among 0 - 4 year-olds (59 % of all CTs) [14]. Especially, children are of concern since they are more sensitive to radiation-induced malignancies, such as leukaemia and brain tumours, and have a longer lifespan with ongoing harmful effects of radiation [15, 16]. Induction of leukaemia or brain tumours has been estimated to be 1 in 10,000 from a single CT scan in children younger than ten years. The same study also estimated a substantially increased risk of cancer after multiple scans with radiation doses from two to three head CTs (about 60 mGy cumulative brain dose) to triple the risk of brain tumours (RR 3.32) compared with doses less than 5 mGy [17]. Recent decision rules and head trauma guidelines from the USA and the UK have tried to address this issue. The PECARN study [7] has not been validated in the Scandinavian setting, but follow-up studies after implementation in the USA have shown a decrease in CT rate from 21 % to 15 % [18].

Presently, there are no specific guidelines for children with MHT available in Scandinavia. According to the survey of the management of paediatric MHT in both Sweden and Denmark, predominately local guidelines exist, often based on the adult head injury guidelines from the Scandinavian Neurotrauma Committee (SNC) from the year 2000 [19–21]. As a result of the lack of guidelines, there are large discrepancies in the management within and between the Scandinavian countries [19, 20]. International efforts have resulted in several paediatric guidelines [7, 22]. Although these are based upon sound methodology, they were not designed for the Scandinavian health care system. Also, during the validation process and introduction of the revised adult head injury guidelines [23], including the clinical introduction of serum marker S100B, interest has been raised for the possible use of this biomarker in paediatric head trauma management. The development of a head trauma guideline specifically for children, with the Scandinavian health care setting in mind, is therefore warranted.

### Aim of the study, target population

In the present report we aim to present evidence- and consensus-based guidelines for initial management of minor and moderate head trauma in children. The purpose of the proposed guidelines is to assist physicians in the initial management of children (<18 years of age) with head trauma within the first 24 hours following trauma, particularly to determine those children who need a head CT and/or in-hospital observation and those who can be directly discharged from the ED. The guidelines exclude children with severe head trauma as defined below (see definitions). They are intended for use by physicians in the ED, including paediatric EDs and to some extent general practitioners; hence, with focus on physicians who are not experts in the field of head trauma management. The guidelines are not intended for nurses or non-medical professionals.

The primary goal of the study was to identify all paediatric patients in need of intervention, such as neurosurgical and/or intensive care or who have an intracranial injury (critical patient-important outcome), and secondarily, those paediatric patients with any traumatic intracranial injury, including skull fractures (important patient-important outcome) following minor and moderate head trauma.

## Methods

**Fig. 1:**
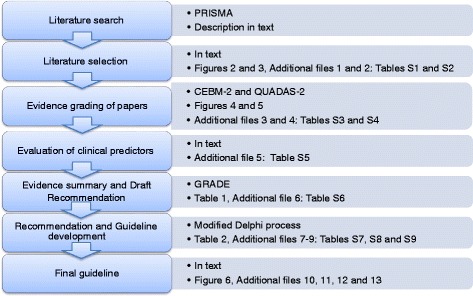
Diagram of the overall work process and methodology

The fundamental policy for developing the guidelines was to follow the Appraisal of Guidelines for Research and Evaluation (AGREE)-II guideline development framework [24]. Standardised and recommended assessment tools, such as the QUADAS (Quality Assessment of Diagnostic Accuracy studies)-2 tool [25] and CEBM (Centre of Evidence Based Medicine)-2 [26], were used for the assessment of the quality of evidence for the different studies, as well as the GRADE (Grading of Recommendations Assessment, Development and Evaluation) system for development and assessment of proposed recommendations [27, 28]. As evidence in some areas was absent and/or inadequate, a modified Delphi process was used for certain issues and for agreement on the recommendations and guideline. The methodological process and work flow is shown in Fig. 1.

### Task force, working group and stakeholders

The SNC consists of neurosurgeons, anaesthesiologists, neuroanaesthesiologists, neurologists and radiologists with special expertise in neurotrauma. A task force was formed within the SNC, consisting of the three authors (RÅ, CR, and JU), tasked to initiate the research by retrieving and assessing the evidence, determining the quality and drafting the recommendations as well as the guideline proposal. The working group, consisting of all the SNC members, were updated on the process twice yearly.

Stakeholders consisting of paediatricians, neuropaediatricians, paediatric anaesthesiologists and paediatric surgeons from the Scandinavian countries were invited to comment upon and evaluate the proposed guidelines following the AGREE II method during the Delphi process. A modified Delphi process, including the task force, the working group and stakeholders, was held for the consensus part of the process. Since the SNC working group mainly consists of neurosurgeons, anaesthesiologists and neurologists, we chose to invite stakeholders within the field of paediatrics in order to get the expert opinion from care-givers who manage these children in the paediatric ED, paediatric wards, paediatric intensive care units and rehabilitation departments. Stakeholders known to have a paediatric trauma interest were asked to participate.

### Definitions

Head trauma is defined as any physical hit or blow towards the head, which may or may not lead to an injury of the underlying brain. We consider a traumatic brain injury (TBI) to be a possible consequence of the traumatic event towards the head. The severity of head trauma was defined according to a modification of the HISS classification [5], similar to the definitions in the revised Scandinavian adult head injury guidelines [23]. In this modified classification, moderate head trauma was defined as GCS scores of 9 to13 on admission [29], mild head trauma represented patients with an initial GCS score of 14 to 15, with or without neurological deficits, and minimal head trauma with GCS score of 15 and no other risk factors. Risk factors are considered to be any symptom or condition specified in the guidelines as a predictive factor of intracranial complication after the head trauma. Severe head trauma (GCS score ≤ 8) [30, 31] was not included in the guidelines, since these patients are managed using a different protocol and always receive both immediate head CT and in-hospital admission due to a high risk of intracranial injury. Neurological deficit was defined as any focal deficit or pathological finding in the clinical neurological examination, e.g. paresis of the extremities, cranial nerve affection, anisocoria, ataxia or aphasia.

The definition of children was predefined as any person below the age of 18 years. The search criteria included synonyms for “children” and were not limited by any specific age range.

“CT findings” were defined as any traumatic finding on head CT, including linear skull fractures. The CT findings group was added since it was not always possible to separate linear non-depressed skull fractures from the statistical data given in the study.

Intracranial injury (ICI) was pre-defined as any intracranial pathology on head CT, such as intracerebral haematomas, epidural and subdural haematomas, traumatic subarachnoid haemorrhage, pneumocephalus, depressed skull fracture and presence of skull base fracture, except isolated linear non-depressed skull fractures. Neurosurgical intervention was defined as any neurosurgical procedure for cranial or intracranial injury within the first week following trauma, but also included neurointensive care measures as not all ICIs are subjected to neurosurgery.

Patient-important outcomes (neurosurgery, ICI and “CT findings”) were rated according to GRADE methodology as having a “critical” or an “important” level of outcome importance [32]. Both ICI and neurosurgical intervention were assigned a level of “critical patient-important outcome”. The group “CT findings” was assigned a non-critical but still important level of outcome importance, thus weighted slightly less in the assessment of relevant risk factors and recommendations.

### A priori assumptions and decisions

The task force decided that the initial head CT would be considered the method of choice for acute diagnosis of intracranial complications following head injury. The use of magnetic resonance imaging (MRI) was not considered to be useful in initial management, mainly due to the lack of availability, as well as current practical issues, of acute MRI in the Scandinavian countries in present clinical care. Although MRI is generally better at detecting intracranial injuries, the duration of MRI is much longer and requires full co-operation from the child. An acute MRI is less time consuming, but the quality is presently similar to a CT in detecting intracranial pathology, however, with a higher risk of missing a skull fracture [33].

The use of skull radiographs as an initial method of diagnosing skull fractures before considering a head CT or observation was discussed within the task force and working group but unanimously rejected to be included in the guidelines. Former studies have recommended skull radiography in otherwise asymptomatic infants with a head trauma and scalp haematoma in order to find a skull fracture [34, 35]. In these patients, a CT would be indicated, as the risk of intracranial injury is higher when a skull fracture is present. However, skull radiographs are no longer used in the Scandinavian countries as the primary radiologic investigation as they do not reflect intracranial injury with sufficient sensitivity or specificity [36]. Also, recent data show that isolated skull fractures, in children who are otherwise neurologically intact following head trauma, have little impact on patient outcome [37].

We do not consider later aspects of management of e.g. post-concussion syndrome, rehabilitation or any surgical specifics concerning surgical or medical management of intracranial complications. We also agreed that any pathological traumatic CT finding should lead to a period of in-hospital admission and observation.

Not all patients, especially children, can be subjected to an initial head CT, and absence of ICI was considered for all those who were not diagnosed with neurological deficits, ICI or death, determined after clinical follow-up. In this sense, absence of ICI is more correctly describing absence of clinically important brain injury, rather than ICI. However, for practical purposes, the term ICI was used. A few studies used a reference standard of “clinically important traumatic brain injury” (ciTBI), defined as death from traumatic brain injury, neurosurgery, intubation >24 h or hospital admission ≥2 nights. With similar reasoning as above, ciTBI lies between ICI and neurosurgery in terms of outcome importance but was for practical reasons classified as ICI. During the GRADE assessment, these data were weighted higher with reference to critical patient-important outcomes [32].

The subject of non-accidental injury (NAI) or child abuse is complex. The proposed guidelines are mainly based on data relying on proper history and assessable symptoms, both of which can be difficult to assess, especially due to weak or potential bias information about the historical event in NAI children. The task force, therefore, chose not to include studies focussing on NAI in the guidelines, but to rather raise increased awareness of the problem. In the Scandinavian countries, suspicion of child abuse should immediately be reported to the social services and, due to legal aspects, be thoroughly investigated and injuries extensively documented both clinically and radiologically [38].

The Scandinavian health care system is somewhat different from the US and the UK systems. Clinical observation can be done by admission to the hospital children’s ward and in some hospitals there is a short-term observation ward in close vicinity of the ED. These short-term observation wards are not exactly similar to the paediatric observation units in the USA [39] since the Scandinavian short-term observation wards may have their paediatric resources reallocated to the children’s wards during evenings and nights. Direct admission for in-hospital observation to the children’s ward was recommended in the guidelines when the required observation time exceeded 24 hours. Children requiring observation for a shorter time can be admitted for observation in the short-term observation ward or children’s ward, depending on the resources of the hospital. This is defined in the guidelines as short-term observation.

### Search strategy

Two clinical search questions were assessed within the task force group: (Q1) *“Which paediatric patients with head trauma need a head CT and which may be directly discharged?”* and (Q2) “*Which paediatric patients with head trauma need in-hospital observation and/or repeat head CT?”*

Original studies were found by searching Medline (PubMed), EMBASE, and the Cochrane library. Since the Cochrane library includes review articles only, the reference lists of potentially interesting reviews were checked for original papers missed in Medline search, but still fulfilling the search criteria for inclusion. The reference lists of all included studies were also hand-searched for additional investigations. Publication dates between 1 January 1985 and 18 November 2013 were used as a time frame in all searches. Before 1985, CT was not used widely in this patient group.

The pre-specified key words used for Q1 were: (“head trauma” OR “brain injury” OR “head injury” OR “traumatic head injury” OR “traumatic brain injury”) AND (management OR prediction OR predictor OR decision rule) AND (children OR infant OR neonate OR pediatric OR paediatric).

The key words “concussion” and “commotio cerebri” are common denominates for mild head trauma, but are most commonly affiliated with “post-concussion syndrome” and sports concussion. A separate search for (“concussion” OR “commotio cerebri”) AND (management OR prediction OR predictor OR decision rule) AND (children OR infant OR neonate OR pediatric OR paediatric) was made without finding any additional studies suitable for inclusion in the final review.

The key words for the Q2 search were: (“head trauma” OR “brain injury” OR “head injury” OR “traumatic head injury” OR “traumatic brain injury”) AND (management OR prediction OR predictor OR decision rule) AND (children OR infant OR neonate OR pediatric OR paediatric) AND (hospitalization OR hospitalisation OR observation OR admission OR discharge OR delayed) OR (normal OR negative OR repeat OR multiple OR serial OR follow-up) AND (CT OR CCT OR computed tomography).

### Selection criteria and study eligibility

The searches were independently performed by two authors (RÅ and CR). Study titles were examined independently (RÅ, CR) and studies were chosen very liberally. Titles that were clearly irrelevant were excluded. Abstracts were examined independently by RÅ and CR and any discrepancies were solved by discussion and consultation with the third author (JU). Full text papers were retrieved by accessing different institutional libraries and, as the last attempt, by trying to contact the authors of the studies. All retrieved full-text papers were independently examined by two authors (RÅ, CR) and any discrepancies were resolved and discussed with the third author (JU). Additional papers from references were contributed by all three authors (RÅ, CR, and JU) and examined independently by the whole task force group. The retrieved full-text papers in languages other than English were translated and reviewed by RÅ and CR.

Only original studies were selected for inclusion in the final review. Systematic reviews, reviews or editorial letters were excluded, though the bibliographies were first examined for potentially interesting articles. Studies were included for further analysis if more than 50 % of the patients had a GCS score of 9 to15 on arrival to ED. Any study including children with severe head trauma only was excluded. Studies were also excluded if the patient material included fewer than 30 children or if it was not possible to separate children’s data from adult’s data. We did not set any further specifications to the definition of “a child”. Two studies included “children” up to 21 years of age, although with a mean age below 9 years.

Regarding the clinical question, Q1, studies were included if they reported at least one predictive risk factor related to either positive CT findings, ICI or need for neurosurgery. Studies were included in the final review if information regarding true positives (TP), true negatives (TN), false positives (FP), and false negatives (FN) could be extracted. This was important for further data analysis in order to be able to compare and evaluate the clinical relevance of the different risk factors reported.

For the second clinical question, Q2, studies including paediatric patients with an initial CT scan (normal or abnormal) after minimal to moderate head injury were initially included. Studies containing information about the clinical relevance of repeat or routine CT scan and/or the necessity of in-hospital observation after head injury were included.

### Data extraction and quality assessment

Data were mainly extracted by one author (RÅ) and checked by random sampling by a second author (CR). All data were entered into a predefined protocol containing information regarding the number of patients included, inclusion and exclusion criteria, number of CTs, ICIs and neurosurgeries related to specific risk factors. Evidentiary tables were constructed to summarise the Q1 and Q2 studies.

Quality assessment was independently performed by two authors (RÅ, CR) for all studies included in the final review. Quality of studies was assessed according to the CEBM-2 diagnostic criteria [26] and the QUADAS-2 tool [25]. Quality ratings for CEBM-2 range from 1 to 5, where 1 is the highest rating, given to systematic reviews or cross sectional studies with a consistently applied reference standard and blinding and 5 the lowest rating for papers with mechanism-based reasoning. No papers were given the ratings 1 or 5, since there were no systematic reviews included or any without acceptable statistical reasoning.

The QUADAS-2 includes four key domains regarding: 1) patient selection, 2) index tests, 3) reference standard, and 4) flow and timing. All domains are rated with regard to risk of bias, and the first three items are also rated in terms of concerns regarding applicability to the research question. The domains are rated as high, low or unclear risk/concern. Discrepancies were first discussed between the two authors RÅ and CR and if uncertainties still remained, a third author (JU) was consulted. Discussions were made until full agreement was achieved in the task force.

### Data analysis

In accordance with the previous methodology for the Scandinavian head injury guidelines for adults, we did not perform a meta-analysis on the data prior to the development of the guidelines. Such an analysis, especially in the presence of heterogeneous data, may be misleading. Instead the task force group presented the non-combined data and quality assessment for the working group and stakeholders prior to the consensus process. This data gives the process more transparency and avoids misleading interpretations. Individual positive likelihood ratios (PLR) and negative likelihood ratios (NLR) were calculated for each risk factor related to the corresponding reference test (CT findings, ICI or neurosurgery). The prevalence of the risk factors and the positive reference test for the given risk factor were also calculated. These values are important in judging the impact on a risk factor on patient flow and for considerations included in the GRADE process. For the clinical question, Q2, we present only descriptive analysis.

### Evidence summary and recommendation draft

The GRADE system for diagnostic accuracy studies was used for grading of the important risk factors in relation to the pre-specified critical and important outcomes (neurosurgery, ICI or CT findings) [32]. The GRADE system is widely used in development of recommendations and allows consideration of aspects other than the level of evidence when determining the strength of the recommendations (Table 1) [23, 40, 41]. The evidence for the clinical predictor was initially considered high if derived from cohort studies reporting patients with diagnostic uncertainty and appropriate reference standards.

**Table 1:** GRADE system for rating quality of evidence and strength of recommendation [41]

Factor	Description
Evidence	
High quality	Considerate confidence of the estimate effect. Further research is very unlikely to change our confidence in the estimated effect.
Moderate quality	Confidence that the estimate is close to the truth. Further research is likely to have an important impact on our confidence in the estimate effect and may change the estimate.
Low quality	Limited confidence in the effect. Further research is likely to have an important impact on our confidence in the estimate effect and is likely to change the estimate.
Very low quality	Little confidence in the effect estimate. Any change of effect is uncertain.
Recommendation	
Strong: “We recommend…”	A strong recommendation indicates that most well-informed people will make the same choice.
Weak: “We suggest…”	A weak recommendation indicates that the majority of well-informed people will make the same choice but a substantial minority will not
Uncertain: “We cannot recommend…”	No specific recommendation for or against

Factors influencing the strength of the recommendation include quality of evidence, risk/benefit aspects of presumed patient-important outcomes, costs and uncertainty concerning values and preferences

*GRADE* Grading of Recommendations Assessment, Development and Evaluation

Evidence could be downgraded or upgraded based on six different *parameters*: 1) risk of bias (bias of selection, verification, observer, or reporting), 2) outcome indirectness (the balance between the presumed influence on patient outcome of the test result in relation to the complications and resource use of the test), 3) inconsistency (large differences in prevalence of reference tests, prevalence of risk factors, PLR or NLR; or differing general results between studies), 4) impreciseness (studies with small number of patients and few positive CT, ICI or neurosurgery events), 5) suspicion of publication bias (small number of studies, industry funding), and 6) large effect (exceptional study with presumed large influence on patient-important outcome) [42].

All three authors in the task force graded the clinical predictors and formed the recommendation draft and flow-chart of the guidelines.

### Recommendations and guideline development – the modified Delphi process

Based upon the recommendations, a draft of the guidelines was constructed by the task force. Following this, we used a modified Delphi process including a nominal group technique for consensus measure and development [43, 44], involving the working group and stakeholders as previously described. The Delphi process typically involves three e-mail rounds in which a group of experts give their opinions and rate the research questions. The results are summarised and re-distributed for re-rating where the participants have the opportunity of changing their score in view of the group’s response. The nominal group technique is a structured meeting gathering relevant experts to discuss and reach consensus about a given issue. Each participant, in turn, contributes and comments on the issues, a method which facilitates equal participation of all group members [45, 46]. According to an a priori decision, at least two rounds of consensus would be performed, irrespective of the results from the first round. The ratings of the recommendations and guideline drafts were done according to a 7-point scale of the AGREE-II instrument [47]. The a priori criteria for consensus (acceptance or rejection) or the lack of consensus are shown in Table 2*.*

**Table 2:** A priori established seven-point response scale and criteria to determine acceptance, rejection or lack of consensus for recommendations and guidelines

	Level of agreement						
	Strongly disagree	Disagree	Moderately disagree	Neither agree or disagree	Moderately agree	Agree	Strongly agree
Score	1	2	3	4	5	6	7
Criteria	75 % of respondents score ≤ 3 on the 7-point scale			All other situations	75 % of respondents score ≥ 5 on the 7-point scale		
Result	Consensus against			No consensus	Consensus in favour		
Action	Reject recommendation			No consensus has been reached	Accept recommendation		

In the first round, the recommendations, data from included studies (including data for CEBM-2, QUADAS-2, and GRADE evaluations), together with the guideline draft, were sent by e-mail to the working group and stakeholders. Ratings, which includes rating of the: 1) scope and purpose, 2) stakeholder involvement, 3) rigour development, 4) clarity of presentation, 5) applicability, 6) editorial independence, and 7) an overall guideline assessment [47], including feedback, were collected and the task force summarised the ratings and opinions and adjusted the guideline draft based upon the response. The adjusted guideline draft was sent out by e-mail a week before the following consensus meeting. The ratings and comments on the recommendation and guideline draft were presented at the consensus meeting (one day), which was held in conjunction with a two-day SNC-meeting in January 2015 in Copenhagen, Denmark. Results were discussed and additional suggestions for improvements were made. During the consensus meeting the task force revised the recommendations and guidelines accordingly and the second Delphi round was performed. The results were summarised after the consensus meeting and it was agreed upon that in the event of the working group and stakeholders not reaching consensus on some of the issues, a third Delphi process would be performed by e-mail in April 2015.

## Results

**Fig. 2:**
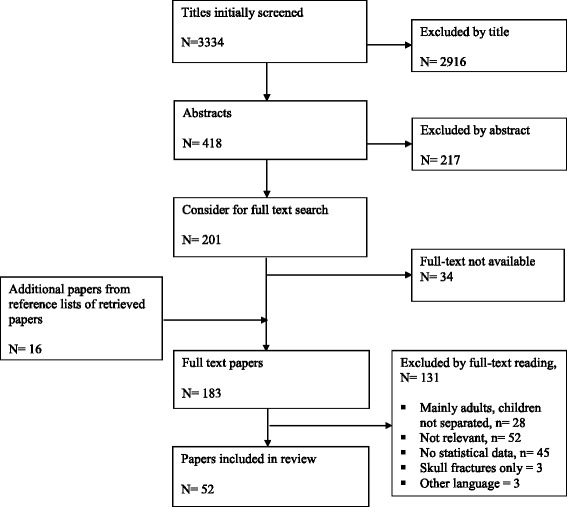
PRISMA (adapted preferred reporting items for systematic reviews and meta-analyses) diagram showing the review process for clinical question 1: *Which paediatric patients with (non-severe) head trauma need a head CT and which patients may be directly discharged?*

**Fig. 3:**
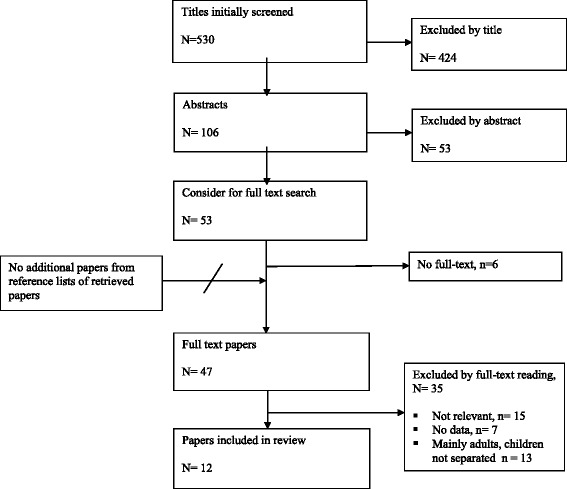
PRISMA (adapted preferred reporting items for systematic reviews and meta-analyses) diagram showing the review process for clinical question 2: *Which paediatric patients with (non-severe) head trauma need in-hospital observation and/or repeat head CT?*

A flow diagram of the working process is given in Fig. 1. The search and selection process for the two clinical questions is shown in Figs. 2 and 3*.* For the first clinical question, 52 papers satisfied the inclusion criteria (Fig. 2). These studies included 118,265 individual children, 25,794 below 2 years of age. Head CT was reported for 46,218 children (39 %). Of these, 4486 (9.7 %) had a trauma related CT finding, mainly skull fracture. ICI’s were reported for 2569 children (2.2 %; 2184 ICI and 385 ciTBI), and neurosurgery for 702 children (0.6 % of the whole study population).

For clinical question 2, we included 12 papers (Fig. 3), which included a total of 16,181 individual children. Descriptive data for the included studies of both clinical questions are shown in the evidentiary tables (Additional files 1 and 2: Tables S1 and S2).

**Fig. 4:**
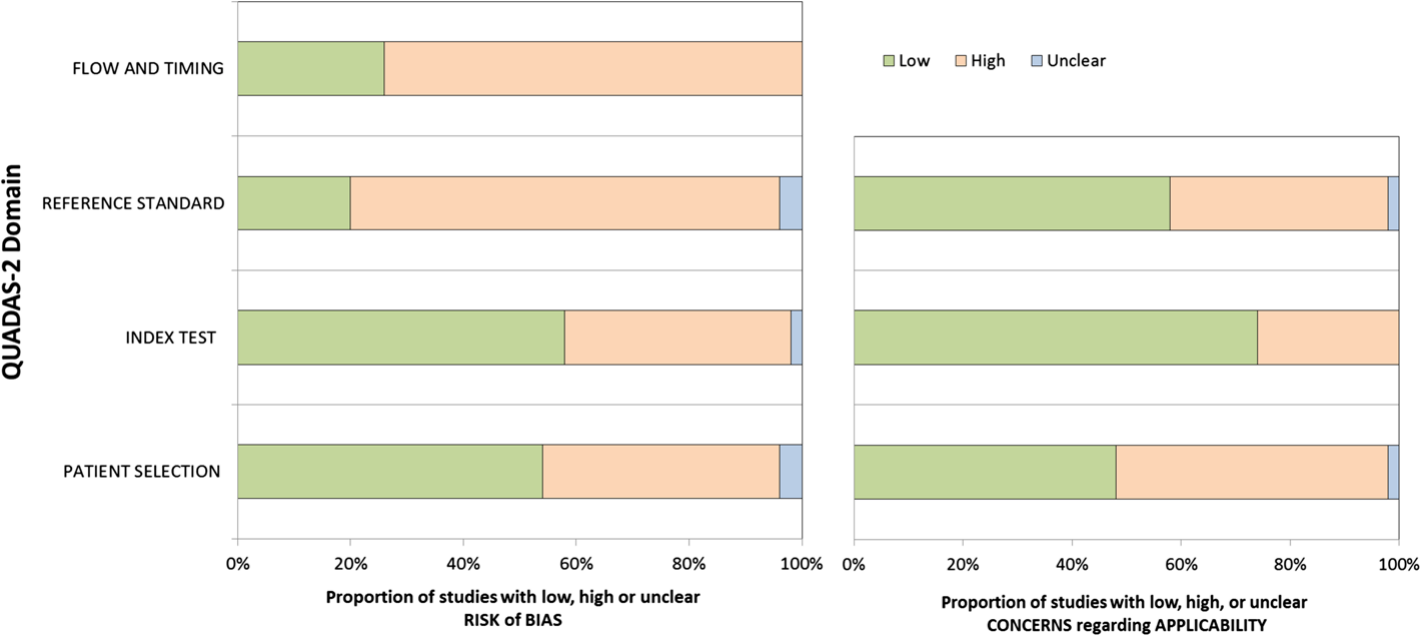
Summary of QUADAS-2 for clinical question 1: *Which paediatric patients with (non-severe) head trauma need a head CT and which patients may be directly discharged?*

**Fig. 5:**
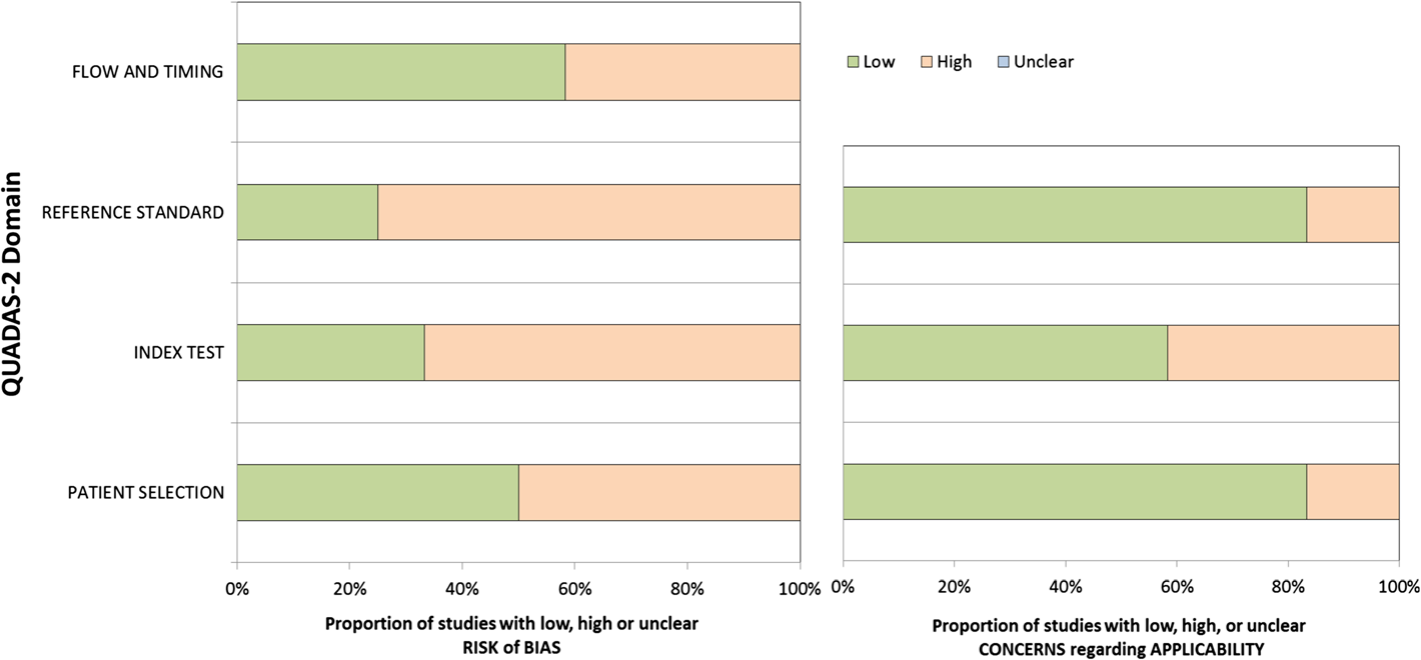
Summary of QUADAS-2 for clinical question 2: *Which paediatric patients with (non-severe) head trauma need in-hospital observation and/or repeat head CT?*

For clinical question 1, CEBM-2 varied between 2 and 4, with a median of 3. For question 2 the studies reached a CEBM-2 score of 3 to 4, with a median of 3.5. The QUADAS-2 evaluation of question 1 showed substantial bias regarding the reference standard and also flow and timing. The reference standards were of varying quality in the studies, some lacking adequate follow-up for those not receiving a CT scan. Thus, not all patients received the same reference standard, and some did not receive a reference standard at all. QUADAS-2 evaluation of the clinical question 2 studies showed substantial bias, especially regarding the index test (not blinded to reference standard) and the reference standard (lack of follow-up and results not blinded to the index tests) (Figs. 4 and 5, Additional files 3 and 4: Tables S3 and S4).

Clinical predictors with the according source study, PLR, NLR, prevalence of risk factors, and prevalence of the reference tests (CT finding, ICI or NS) are shown in Additional file 5: Table S5.

### Recommendations

Based upon the evidence and the evaluation using the GRADE system, drafts of recommendations were made (Additional file 6: Table S6). Proposed guidelines based on the recommendations, including a flowchart, written discharge advice and observation schedule, were constructed by the task force. The recommendation draft and proposed guidelines, accompanied by all tables and figures, were reviewed by the working group and stakeholders in the Delphi process previously described.

Following round 1, the discussion mainly concerned points 4 and 5, but minor adjustments were also made to points 3 and 10, the latter being the guideline flowchart. The changes all complied with the evidence summarised in Additional file 7: Table S7. During the consensus meeting it was decided to split “LOC” into “LOC ≥ 1 min” and “suspected/brief LOC”, due to slight differences in their predictive risk, and the wording “altered mental status” was changed to GCS 14, as discussed below. Point 7 was vividly discussed as the majority did not agree on recommending early discharge of a child e.g. with clinical evidence of skull base fracture or following a seizure, despite a normal initial head CT.

Following round 2, consensus was not reached for points 7, 8, and 9. The task force revised the recommendation for point 7 and it was made more specific in regard to which patients could be discharged after a normal head CT. Improvements of the written discharge information and observation schedule were also made according to earlier discussions at the consensus meeting (Additional file 8: Table S8).

A third Delphi round was therefore performed by e-mail in early spring 2015. Ratings were made for points 7-9 and consensus was reached for all three points (Additional file 9: Table S9).

The final evidence-based recommendations are presented below. For clinical question 1: *“Which paediatric patients with head trauma need a head CT and which may be directly discharged?”*

1. We recommend that all children with an ED admission GCS score 13 or below after head trauma should have a head CT scan. (Evidence grade: very low, Recommendation: strong).

The evidence was initially of moderate quality but downgraded due to limitations in study design (mainly selection bias towards a more severely injured patient group), indirectness and inconsistency (large differences between the prevalence of risk factors and likelihood ratios). The evidence level was upgraded due to a large effect of one of the studies for the important outcome of ICI. The strength of the recommendation was, however, by the task force perceived as strong, when considering the seriousness of the potential intracranial complication and the health economic impact of missing a patient with a neurosurgical lesion [22, 48–60].

2. We recommend that children with (a) neurological deficit related to the trauma, (b) post traumatic seizure, or (c) clinical signs of skull base or depressed skull fracture should have a head CT scan. (Evidence grade: very low, Recommendation: strong).

The evidence was initially of high quality, but downgraded due to limitations in study design (selection bias), indirectness (lack of description of outcome measures and follow-up) and inconsistency (large differences in prevalence of risk factors and likelihood ratios). There was no upgrading of the evidence level. The recommendation was perceived as strong when considering the relatively low prevalence of the predictive factors compared to the severe influence on patient outcome if the patients with ICI or a neurosurgical lesion were missed; (a) [8, 22, 48, 51–53, 61–73], (b) [22, 48, 49, 51–53, 55, 63–65, 67–78].

Suspicion or evidence of skull fracture was found to be a strong predictor for ICI, and especially high risk was found for evidence of depressed skull fractures and clinical signs of skull base fractures [7, 8, 22, 49, 51, 53, 56, 62–64, 70, 71, 73, 79, 80]. A palpable fracture will automatically give a suspicion of depressed skull fracture. Linear skull fractures are generally not palpable, but might give rise to a scalp haematoma. The prevalence of linear fractures was relatively high and linear fractures are less predictive of intracranial injury compared to depressed or skull base fractures. The task force therefore chose to separate these according to their risks, such that patients with clinical evidence of depressed or skull base fractures are recommended for a CT and those with temporal scalp hematomas alone are recommended for observation, according to recommendation 5.

3. We recommend that children with (a) GCS score 14, (b) loss of consciousness for > 1 min after head trauma or (c) children with coagulation disorders or with anticoagulation therapy should be either admitted for in-hospital observation or have a head CT. (Evidence grade: very low, Recommendation: strong).

The initial recommendation draft included “altered mental status”, defined according to Kupperman et al as a predictor [7]. Since irritability, somnolence and confusion are all included in the definition of a GCS score lower than 15, the “altered mental status” was changed to “GCS score 14” after the first Delphi round [7, 22, 51, 53, 55–58, 62–65, 67, 68, 75, 77–82].

The evidence for prolonged LOC (≥1 min) [7, 22, 48, 49, 52–54, 67, 71, 83] as a predictive factor for intracranial complications was slightly higher (low evidence) than for unspecified LOC (very low evidence) [6–8, 48, 49, 51, 53, 55, 60–65, 68, 70–75, 78–80, 83–86]. This is mainly due to imprecision and indirectness, and with a very high prevalence of the risk factor in some studies. An obvious bias was also that some studies also had LOC as inclusion criteria, increasing the prevalence and severity level of the study population. There was also a slight increased risk of ICI for LOC of 1 min or longer.

Most studies excluded patients with coagulopathy, as this has been considered to be associated with high risk for developing intracranial bleeding after trauma. Two studies investigated coagulopathy as a potential risk factor [53, 63] and especially one study found coagulopathy to be a strong predictive factor for intracranial injury [53]. The evidence for this predictor was very low, as there was no further description of the risk factor or potential confounding factors. Selection, imprecision, and publication bias were the main parameters that lowered the evidence level. The prevalence of children having coagulopathy or in anticoagulant treatment is very low and the number of children with coagulopathy and head injury can be considered to be even lower. The task force therefore concluded that due to the potentially increased risk, these children should not be sent home immediately from the ER and thus instead are recommended in-hospital observation to follow development of eventual symptoms or a CT scan. Admission, rather than CT only, was chosen as children with coagulation issues are often subjected to numerous radiological procedures, most of them following trauma. We therefore allowed for the treating physician to choose one of these management options.

4. We recommend that children after head trauma with (a) posttraumatic amnesia or (b) vomiting of two or more times [7, 22, 49, 52, 53, 60, 63], should be admitted for clinical observation in the hospital (Evidence grade: very low, Recommendation: strong).

The evidence regarding posttraumatic amnesia [8, 22, 52] was initially high but downgraded due to selection bias and publication bias (very few studies) and indirectness (no specified reference standard for outcome measures). We decided to include prolonged amnesia (> 5 min) [22] in the evaluation of posttraumatic amnesia, since it was considered a strong predictor with moderate level of evidence. Duration of amnesia, especially in a child, is very difficult to determine and impractical, and cannot be properly evaluated in a preverbal child. Following the consensus meeting we therefore decided not to include any time limit for amnesia in the recommendations.

The prevalence of both these predictive factors was relatively high in the investigated studies and when considering the health economic consequences compared to the risk of missing an important intracranial complication, the task force did not find the evidence strong enough for recommending an immediate CT scan. Therefore, we instead recommend in-hospital observation.

5. We suggest that children displaying a GCS score 15 with (a) severe or progressive headache, (b) abnormal behaviour according to guardian [7, 53, 62, 63, 65], (c) brief LOC or (d) if age < 2 years and with irritability or a large or temporal/parietal scalp haematoma, should be observed in the hospital. (Evidence grade: very low, Recommendation: weak).

The quality of the evidence was initially high, but down rated due to selection bias (some studies only included infants), impreciseness, inconsistency, and indirectness.

Severe progressive headache was considered a moderate to weak predictor of intracranial complications and the evidence level was very low mainly due to the high and variable prevalence of the predictor (2-60 %) in the included studies [7, 52, 53, 67].

Irritability was not included in the definition of “GCS score 14 or lower”, since the task force and the working group found irritability more similar to abnormal behaviour than to decreased level of consciousness or confusion. Irritability can be misinterpreted in many ways, and should be understood as an abnormal behaviour to a normal stimulus, not only an angry child. Only two studies investigated irritability as a risk factor for ICI among children [55, 75]. It is a weaker predictor of ICI compared to GCS score 14 or drowsiness, and the level of evidence was very low.

Three studies investigated the occurrence of scalp haematoma based on size or location. All but one study only included children younger than two years old. Size of haematoma was divided into “small, barely palpable”, “moderate and easily palpable” and “large, boggy consistency”. Only children with large boggy haematomas had a clearly increased risk of intracranial complication [35, 49, 75]. Temporal haematoma was found to be a moderate predictive risk factor for ICI, whereas occurrence of parietal haematoma was considered to be a weak risk factor for ICI [35]. The prevalence of scalp haematomas after head trauma in this patient population is large and the occurrence of ICI low. Recommending a CT would lead to an enormous increase in unnecessary radiation to the child. We therefore recommend in-hospital observation for these children.

For the second clinical question *“Which children with non-severe head trauma need a repeat CT and/or in-hospital admission?”* the following recommendations were made:

6. We recommend that repeat CT should be performed in patients with clinical or neurological deterioration. (Evidence grade: very low, Recommendation: strong).

Evidence was initially considered of moderate quality (studies 1-7 in Evidentiary table Q2), but was downgraded due to serious selection bias, inconsistency and impreciseness [87–93]. Routine repeat CT is not recommended for all admitted children after head trauma. Patients with mild head trauma and a normal initial head CT have a very low risk of radiological progression on a routine repeat CT if symptoms are unchanged or improved [87–89]. The evidence did not give any conclusive results for children whose initial head CT showed an intracranial injury. One study showed that repeat CT only had a clinical consequence in the case of clinical deterioration or if the patient was suspected to be a victim of NAI, despite ICI on initial CT [90]. Another study concluded that children with moderate or severe head trauma and improvements of GCS score after the initial CT do not require routine repeat CT within 48 hours [91]. Three other studies have concluded that patients with high-risk intracranial lesions on the initial CT should have a repeat CT within 24-48 hours, due to the risk of radiological progression and change in management. The definition of high-risk and low-risk lesions varies in the different studies [89, 92, 93], thus any specific recommendations were not possible to be based upon these studies.

7. We suggest that those patients with mild head injury and a normal neurological examination and with an initial head CT without any pathological findings related to the head trauma, can be discharged (Evidence grade: low evidence, Recommendation: weak).

Evidence based on Q2 studies 8-12 was initially considered of high quality, but downgraded due to impreciseness and indirectness [39, 83, 94–96]. All five studies included a total of 14,486 children with MHT, primarily GCS score 14 to 15, although two studies also included some with GCS score 9 to 13. Although not consequently specified, we could assume that none of the patients included in these studies had focal neurological deficits. We therefore conclude and suggest that patients with GCS scores of 14 to 15 and no neurological deficits and without any pathological findings on CT related to the trauma can be discharged. Three out of five studies included patients with a normal CT only [94–96], one study concluded that the finding of a skull base fracture on CT might imply a high risk of the child requiring a longer observation time (>24 h) than provided in their observation unit [39] and in the last study, although it included patients with basilar skull fractures [83], there were not enough data to draw conclusions about early discharge when there is no intracranial injury in the presence of a basilar skull fracture. This latter issue was discussed in the consensus part before finalisation of the guidelines (see Guidelines).

### Guidelines

**Fig. 6:**
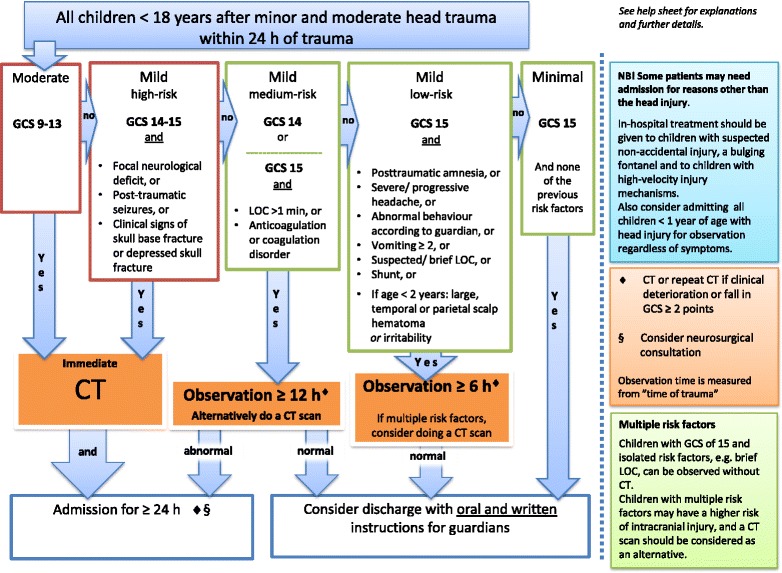
Scandinavian guidelines for initial management of minor and moderate head trauma in children

Based upon the recommendations, the guidelines and the guideline flow-chart were constructed (Fig. 6, Additional file 10: Help sheet). Similar to the Scandinavian adult head injury guidelines, the recommendations were divided into moderate, mild and minimal head injury groups and the mild head injury group was further sub-divided into high-risk, medium-risk and low-risk depending on the GCS score and predictive risk factors.

During consensus discussion, the working group agreed to add the occurrence of shunts into the guidelines. Thus, we suggest that children with ventricular shunts and no specific symptoms should be observed for at least six hours. According to available evidence, patients with shunts do not have an increased risk of intracranial injury or neurosurgery compared to those without. Most studies, however, excluded children with shunts or other known neurological disorders in their analysis. Only one study by Nigrovic et al. focusses specifically on 98 children with shunts admitted to the ED after minor head injury [97]. They found that the risk of having a clinically important traumatic brain injury was very small; only one child had a chronic subdural haematoma which was larger than seen on the previous CT. In the event of an intracranial haematoma, there is a theoretical risk of a more rapid expansion of the haematoma among patients with a ventricular shunt, due to the increased drainage of cerebrospinal fluid resulting in less counter-pressure against the hematoma. For shunted children, there is also the constant question of possible malfunction of the shunt after head trauma and the study by Nigrovic et al. also showed that these children, in comparison to children without shunts, are more subjected to CT scanning after head trauma [97].

Additional factors, such as age < 1 year, bulging fontanel and high-energy trauma, were discussed but not included in the recommendations or guidelines.

Our analysis did not show an increased risk of intracranial injury or neurosurgery in younger children or infants. Infants are in general more difficult to diagnose. They may present with fewer and more unspecific symptoms late in the process. For this reason, it was discussed whether to admit all infants for observation regardless of symptoms (or lack of symptoms) after head trauma, but there was no clinical consensus regarding this. Instead, the task force decided to raise awareness regarding infants by adding this in the NB-box in the help-sheet.

A bulging fontanel as a sign of increased intracranial pressure may be a predictor of intracranial injury after head trauma in infants [75]. A bulging fontanel is normally seen in a crying and tense child (< 2 years), which can be difficult to differentiate from more serious causes, especially for the inexperienced doctors usually on call in the ED. Children with this finding may need a CT scan, although a child with intracranial pathology severe enough to cause bulging of the fontanel should also present with other symptoms, such as irritability, decreased level of consciousness or focal neurological deficits. Therefore, bulging fontanel was for practical reasons omitted in the recommendations, but is mentioned in the guidelines as an extra precaution in the NB-box.

High-velocity road traffic accidents and fall from > 3 m were considered moderate predictors of intracranial injury. The evidence was very heterogeneous with most studies showing a relatively low predictive value, in contrast to some studies showing extremely high predictive values, especially for road traffic accidents [22]. These studies were biased towards selecting any severity of head trauma, and there was no further description of the level of consciousness or severity of the patients admitted due to traffic accidents. High-speed traffic accidents and falls from > 3 m of height are considered high-energy trauma, and these patients will be treated in the Scandinavian EDs according to a trauma protocol; these children routinely receive an extensive full-body examination, often a whole body trauma CT, and are always admitted for in-hospital observation. The task force decided therefore not to specifically include these predictors in the recommendations, but point out that these children should not be immediately discharged from the ED.

During the consensus meeting, the length of in-hospital observation was discussed. The studies from the Q2 search mainly used an observation time of 12-24 hours, even after a normal head CT, but no evidence-based conclusions regarding the duration of in-hospital observation could be drawn from the studies. Consensus regarding a 24 h, 12 h and 6 h observation time, depending on the risk factors, was reached and added in the guidelines. There was a unanimous consensus that children with moderate head trauma and those in the mild, high-risk group, should be observed for at least 24 hours post trauma, regardless of a normal initial head CT, and that children with mild, medium-risk should be observed for at least 12 hours post trauma. The majority of children entering an ED after head injury will be in the mild, low-risk or minimal head trauma groups. After discussion among the working group and stakeholders, there was an agreement to lower the recommended observation time to at least 6 hours for the mild, low-risk group, taking into consideration the impact a longer observation time would have on the paediatric wards, as well as an increased economic impact.

There were divergent opinions regarding the early discharge of MHT patients with normal neurology if initial head CT is normal or without intracranial haemorrhage. The main concern was that this suggests early discharge for children who have had a posttraumatic seizure or have clinical signs of skull base fracture. Children presenting with posttraumatic seizures or skull base fractures are relatively rare, and are more often related to more severe head traumas [98]. So far the evidence for early discharge of these children who also have a normal CT scan is still very weak. Some larger studies exclude patients with basilar skull base fractures [94, 99], although there are some former studies suggesting discharge of these children from the ED [100, 101]. During the consensus discussion it was stated that according to good clinical practice, these children should not be discharged without proper in-hospital observation, as these clinical risk factors are relatively worrying, both to health care professionals and to patients/guardians. There was therefore a consensus that children with a mild, high-risk head trauma should be admitted for observation regardless of a normal head CT. The length of observation was kept to more than 24 h after the trauma, this still being a consensus decision in lack of better evidence.

### In-hospital observation and discharge information to patient and guardian

There were no information regarding the quality of the in-hospital observation routine, nor concerning the discharge information given to parents and children in the included studies. The task force therefore searched the available information sheets from existing head injury guidelines, including the recently revised SNC head injury guidelines for adults [23, 102–105]. A draft for discharge information and in-hospital observation was made and sent to all involved stakeholders and the working group before the consensus meeting. During the consensus meeting, the working group and stakeholders agreed upon the basic information of a refined draft, also including recommendations for “return to play” after concussion. The finalized versions were revised by the task force, and sent out by e-mail for the 3^rd^ modified Delphi round (Additional files 11, 12 and 13). The observation sheet is intended as guidance for physicians and nurses in the paediatric wards managing children with mild to moderate head injuries with or without a verified intracranial injury. It includes the minimum requirements for observing a child with head trauma. The advice regarding stepwise return to play is mainly based on the consensus statement on concussion in sports by McCrory et al [106]. It was agreed upon that children (< 18 years of age) should have a more conservative approach than adults before return to play, as the brain is more vulnerable to second impact syndrome or increased risk of brain swelling in children and adolescents [107].

### Implementation, monitoring and future aspects

The value of these guidelines lies in widespread use and implementation. Before widespread implementation of the guidelines, they first need to be externally validated. The proposed guidelines are therefore planned for clinical validation in the Scandinavian paediatric population in both a retrospective and in a prospective cohort study, primarily to determine the safety of the proposed guidelines, but also to compare the performance of our guidelines to other decision rules. The validation process is similar to the validation of adult head trauma guidelines, which is currently ongoing.

The guidelines will be translated into the different Scandinavian languages and published in the national medical journals, which are routinely read by the members of the national medical societies. Once the guidelines have been validated, further implementation will be led by the SNC members in their respective countries. Educational meetings, pocket cards and guideline apps are known to be useful tools and will be used in the implementation process.

Follow-up on the implementation process will be made one year after commencement by a questionnaire similar to the one developed for follow-up of the adult head injury guidelines. There is rapid development in this area, especially regarding radiologic diagnostic procedures and concerning biomarkers for brain damage following MHT. We estimate that a revision of the guidelines should be performed within four years of publication.

## Discussion

The new Scandinavian head trauma guidelines are primarily aimed as guidance to detect intracranial complications after head trauma in patients needing neurosurgery or medical intervention. They are complementary to the newly revised adult head injury guidelines [23] by using the same severity classification, and apply to all children and adolescents below the age of 18 years. It is a requirement that the physicians have a basic knowledge of the GCS and, although not specified in the guidelines, the paediatric GCS is also applicable for children younger than five years [108]. The guidelines are primarily intended as guidance for physicians who meet this patient category and who are not experts in this field. Physicians who have considerable experience with these patients should naturally be allowed to deviate from these guidelines according to best clinical judgement.

In developing the SNC guidelines, we have taken into account the potentially harmful long-term effect of ionizing radiation derived from diagnostic CT [17] and therefore only recommend referral to CT when clinically justified. The guidelines separate mild, high-risk and mild, low-risk head trauma patients, favouring short-term observation for mild, low-risk patients as an attempt to reduce unnecessary CT scans in children. In comparison, international guidelines, such as the AAP guidelines, the CATCH, the CHALICE guidelines and, to some extent, the PECARN decision rule, seem to advocate a more liberal view on CT scanning of children [7, 22, 49, 109], where our guidelines recommend observation. In the study by af Geijerstam et al, similar patient satisfaction and outcome with either immediate CT and early discharge or in-hospital observation was found [10]. The use of acute MRI would omit the radiation issue, but there are still major issues regarding this technique at the present time, including availability, risks of missing skull fractures [33], and need for sedatives or anaesthesia during the procedure [110]. Eventually, this modality will become more practical, faster, cheaper, and more widely available and could then potentially replace CT scanning for this patient group. However, until this is a clinical reality, CT scanning, with the associated risk, is the diagnostic method of choice. Due to these risks, clinical observation can be used in the intermediate risk group of children with MHT. Children from these groups who display clinical deterioration or fail to improve should have a CT scan. Children with higher risks of brain injury should naturally receive a CT scan as the primary management.

The economic impact of TBI has so far been poorly investigated, especially with regards to minor and moderate head injuries [111]. A recent epidemiological study from New Zealand has investigated the incidence of TBI across different age-groups and TBI severities, including both non-hospitalised and hospitalised patients, and found that the incidence of mild TBI was far greater than estimated in the previous studies from other high-income countries (749 vs 200-550 per 100,000 per year) [112]. In a follow-up study based on these results, the authors also made an estimate of the cost of TBI, according to severity, during the first year and included an estimated life-time cost, where the latter cost-estimate varied from USD 4.636 for mild cases to USD 36.648 for moderate - severe cases [113]. Proper and early diagnosis and avoiding unnecessary hospitalisations and investigations, as well as adequate discharge information for patients and guardians, could help decrease the overall costs.

Morbidity rates are high for moderately and severely brain injured patients [114]. Rehabilitation of both motor and cognitive skills is required and, even if some patients fully recover with respect to their neurological functions, many still suffer from memory, psychological, and social problems [115]. Children have a higher percentage of good outcomes and lower mortality rates than adults [116]. Post-concussive symptoms have been described in 15-50 % of the adult population [117], but exist even in the paediatric population causing memory problems and impaired school performance [118, 119]. The risks and long-term effects of post-concussion syndrome and the socio-economic impacts are not handled in the present guidelines, and are yet to be determined.

The present guidelines do not include the biomarker S100B, since the evidence was considered too low and the number of studies too few. Additionally, the studies included had different cut-off values and various commercially available methods for S100B analysis were used. Serum S100B has been extensively studied among adults and has recently been introduced in the SNC adult head injury guidelines. With proper use of the adult guidelines including S100B, the number of unnecessary CT scans can be reduced up to 30 %, which would naturally be desirable in a paediatric setting. However, the reference levels for children are highly age dependent [120, 121], and a large study confirming the positive results from Bouvier et al is needed before S100B can be included in paediatric guidelines [122]. We intend to follow-up on this important issue in future clinical studies.

There are limitations to this study. The poor quality of the evidence is of major concern mainly due to selection as well as verification bias. Some studies excluded patients with LOC and others included patients with pre-specified symptoms only. The majority of studies excluded patients with bleeding disorders and penetrating injuries. Some older studies performed skull radiography on subsets of patients with suspected skull fractures. CT was only performed if the x-ray showed a fracture. The largest study in our material, by Kupperman et al [7] with a primary endpoint of clinically important TBI, has naturally had a large impact on these guidelines as it includes more than 42,000 children and the study quality is exceptionally good. Since the commencement of the guideline process in 2013, there have been several subanalyses from the same PECARN cohort. These studies analyse the different symptoms (headache, presence of scalp haematoma, and vomiting) as possible predictive risk factor for ciTBI [123–125], and confirm the results of these guidelines. Isolated headache, isolated vomiting, and isolated LOC in children with MHT were considered to indicate a considerably lower risk of ciTBI, and the authors suggest that these children could be observed in the ED without an initial CT scan [123, 124, 126]. These studies will be included for consideration in the next update of these guidelines.

Although the recommendations are based on evidence, there are elements based on consensus in the final guidelines. The invited stakeholders have the largest expertise and interest in paediatric head trauma in Scandinavia and were therefore essential to the consensus process. We chose not to perform a meta-analysis due to obvious heterogeneous data and the questionable value of the summarised values in these cases [127]. We followed the GRADE methodology for guideline development as this also gave us the possibility of considering other aspects, such as health economic issues and the Scandinavian setting, other than the level of evidence. This methodology was judged as the most feasible considering the Scandinavian target population.

## Conclusion

We present the first evidence and consensus based Scandinavian guidelines for initial management of children with minor and moderate head trauma. They address aspects such as selection to CT or admission, repeat CT, monitoring routines and discharge. These guidelines should be validated before extensive clinical use and updated within 4 years due to rapid development of new diagnostic tools within paediatric neurotrauma.

## Additional files

Additional file 1: Table S1. Evidentiary table of studies with reference to clinical question 1: *“Which paediatric patients with (non-severe) head trauma need a head CT and which may be directly discharged?”* (DOCX 50 kb) Additional file 2: Table S2. Evidentiary table of studies referring to clinical question 2: “*Which paediatric patients with (non-severe) head trauma need in-hospital observation and/or repeat head CT?”* (DOCX 32 kb) Additional file 3: Table S3. QUADAS-2 and CEBM-2 evaluation for papers regarding clinical question 1: *“Which children with (non-severe) head trauma should be CT scanned, and which can be discharged?”* (DOCX 30 kb) Additional file 4: Table S4. QUADAS-2 and CEBM-2 evaluation for papers regarding clinical question 2: *“Which children with (non-severe) head trauma need a repeat CT and/or in-hospital admission?”* (DOCX 21 kb) Additional file 5: Table S5. Clinical predictors for pathology on head CT, intracranial injury and neurosurgery after minor and moderate head trauma in children. (DOCX 285 kb) Additional file 6: Table S6. GRADE for predictive factors of interest for CT findings, intracranial injury, and neurosurgery. (DOCX 26 kb) Additional file 7: Table S7. Results of the modified Delphi process, round 1. Delphi point 1 = strongly disagree, Delphi point 7 = strongly agree. Two members did not reply. Delphi points 1-5 refer to recommendations concerning clinical question 1. Delphi points 6-7 refer to the recommendations regarding clinical question 2. Point 8 refers to the written discharge instructions, point 9 to the observation schedule for in-hospital observation, and point 10 refers to the guideline draft including the guideline flow-chart. Result refers to percentage in favour of the recommendations. Cf = consensus for, nC = no consensus, Ca = consensus against. (DOCX 20 kb) Additional file 8: Table S8. Results of the modified Delphi process, round 2. Delphi point 1 = strongly disagree, Delphi point 7 = strongly agree. Two members different from the ones in Delphi round 1 did not reply. Ratings were performed anonymously. Delphi points 1-5 refer to the revised recommendations concerning clinical question 1. Delphi points 6-7 refer to the revised recommendations regarding clinical question 2. Point 8 refers to the written discharge advice, point 9 to the in-hospital monitoring routines, and point 10 refers to the revised guideline draft including the guideline flow-chart. Result refers to percentage in favour of the recommendations. Cf = consensus for, nC = no consensus, Ca = consensus against. (DOCX 21 kb) Additional file 9: Table S9. Results of the modified Delphi process, round 3. Delphi point 1 = strongly disagree, Delphi point 7 = strongly agree. Delphi point 7 refers to the revised recommendation regarding discharge after a normal CT, clinical question 2. Delphi points 8 and 9 refer to the finalized written discharge advice and in-hospital monitoring instructions. Result refers to percentage in favour of the recommendations. Cf = consensus for, nC = no consensus, Ca = consensus against. (DOCX 19 kb) Additional file 10:
**Help sheet for the Scandinavian guidelines for initial management of minor and moderate head trauma in children.** (DOCX 24 kb) Additional file 11:
**Early discharge advice for the guardians of a child who has sustained a minor head trauma.** (DOCX 26 kb) Additional file 12:
**Discharge advice for the guardians of a child who has sustained a mild head trauma or concussion.** (DOCX 23 kb) Additional file 13:
**Observation schedule on admission for children (<18 years) after mild and moderate head trauma.** (DOCX 22 kb)
