# Supplementary material for: Scandinavian guidelines for initial management of minor and moderate head trauma in children
Source: BMC Med. 2016 Feb 18;14:33. doi: 10.1186/s12916-016-0574-x (PMC4758024; doi:10.1186/s12916-016-0574-x)
Supplement: Additional file 1: Table S1. — Evidentiary table of studies with reference to clinical question 1: “Which paediatric patients with (non-severe) head trauma need a head CT and which may be directly discharged?” (DOCX 50 kb) [file 12916_2016_574_MOESM1_ESM.docx]

Table S1. Evidentiary table of studies with reference to the clinical question 1 *“Which paediatric patients with head trauma need a head CT and which may be directly discharged?”*

| No | Study | Year | Design | | N | n < 2y | Age (y) | GCS score | Other inclusion criteria | Exclusion criteria | Follow-up | CT rate % | CEBM-2 | Limitations and comments |
| --- | --- | --- | --- | --- | --- | --- | --- | --- | --- | --- | --- | --- | --- | --- |
| 1 | Nigrovic et al | 2013 | P | Cohort | 98 |  | <18 | 14-15 | Blunt HI <24h and ventricular shunts | Medical record missing information about presence or absence of ventricular shunt | Telephone interview at 1 week and 3 months, if CT was not performed (Blinded to results) | 46 | 2 | comparison to study nr 12 |
| 2 | Xiao et al | 2013 | R | Cohort | 1897 |  | 3-18 | 15 | CT ≤ 2h after trauma and either headache, dizziness or vomiting | Incomplete records, signs of open SF/skull base fracture, no repeated CT when worsening symptoms during observation, or if unimproved symptoms >24h, discharged before improvement, no follow-up | Medical records | 100 | 3 | SB: selected towards specific symptoms |
| 3 | Bouvier et al | 2012 | P | Cohort | 424 |  | <17 | 13-15 | Closed HI and admission < 3 hours | Pregnancy, TBI more than 3h ago, multiple injuries | Telephone interview 24-48h after ED for non-hospitalized children | 15 | 3 | VB: S100B cut-off according to age |
| 4 | Bressan et al | 2012 | P | Cohort pre-PECARN | 288 | 130 | <15 | 14-15 | Blunt HI < 24 h | Trivial injury mechanism AND no signs of HI other than abrasions and lacerations; penetrating trauma, neurodisorder, bleeding disorder, CT elsewhere | Monitored return visits within 2 weeks from discharge. | 7 | 3 | Small SB, VB |
| 4 |  |  |  | Cohort post-PECARN | 356 | 162 |  |  |  | Trivial injury mechanism and no signs of head trauma other than abrasions and lacerations; penetrating trauma, bleeding disorder, CT elsewhere | Monitored return visits within 2 weeks from discharge. Telephone 10-90 days from ED discharge | 8 |  |  |
| 5 | Ng et al | 2002 | R | Cohort | 311 |  | <15 | Any | Acute head injury admitted within 24 h of injury (according to ICD-codes) | None | None | 38% |  | VB: no follow-up, no blinding |
| 6 | Fabbri et al | 2011 | P | Cohort | 2391 |  | 0-10 | “Any” | HI < 24 h | Sedation/intubation before ED (= GCS 3-8), SBP <90mmHg, penetrating injuries | Medical databases and telephone GOS at 6months for patients with ICI | 12 | 3 | SB, VB |
| 7 | Crowe et al | 2010 | R | Cohort | 1065 |  | <16 | Any | ICD-10: S02.0-9, S03.5, S06.0-9, S07.0-9, S08.0-9, S09.0-0 | If CT elsewhere | Medical records for future ED visit, admission or head CT | 19 | 3 | SB: to severe HT; VB |
| 8 | Hallén et al | 2010 | P | Cohort | 111 |  | <18 | Any | HI and informed consent from parent and child | None | None | 20 | 3 | VB |
| 9 | Osmond et al | 2010 | P | Cohort | 3866 | 277 | 0-16 | 13-15 | Blunt HI < 24h and any symptoms: LOC, amnesia, disorientation, persistent vomiting, or persistent irritability if < 2y of age. | Penetrating skull injury, obvious depressed SF, focal neurology, developmental delay or suspected child abuse, return from previously treated HI, pregnant patient. | Structured telephone interview14 days after discharge, if not CT scanned before | 53 | 3 | SB: symptomatic; VB: not blinded |
| 10 | Bechtel et al. | 2009 | P | Cohort | 152 |  | <18 | Any | Closed HI and CT + S100B < 6 hrs post trauma | seizure before, penetrating HI, developmental delay, CP, encephalopathy, Downs syndrome | No | 100 | 3 | SB |
| 11 | Klemetti et al | 2009 | R | Cohort | 485 |  | <17 | Any | ICD-10: S06-S06.9 | Age less than 2 days | Medical records | 50 | 4 | VB, stats not 100% |
| 12a | Kupperman et al. | 2009 | P | Cohort | 42412 | 10718 | <18 | 14-15 | HI < 24 h | Trivial injury mechanism and no signs of head trauma other than abrasions and lacerations, penetrating trauma, neurodisorder, bleeding disorder, CT elsewhere, shunts | Telephone surveys 7-90days and CT if symptomatic, medical records, morgue records | 35 | 2 | small VB, ciTBI |
| 12b | Nigrovic et al | 2011 | P | Cohort | *40113 |  | <18 | 14-15 | See Kupperman + note if stated in the form if observation or no observation before CT decision | see Kupperman + excluding if not mentioned if patient was observed or not before CT decision | See Kupperman et al. |  | 3 |  |
| 13 | Guzel et al | 2009 | R | Cohort | 916 |  | <17 | 13-15 | Head trauma | No clear history of trauma, unstable vital signs associated with major trauma, seizure before ED, bleeding disorder or use of anticoagulation medicine, penetrating skull injury, return for reassessment | No | 37 | 4 | small SB: exclusion if reassessed; VB: no follow-up |

| No | Study | Year | Design | | N | n < 2y | Age (y) | GCS score | Other inclusion criteria | Exclusion criteria | Follow-up | CT rate % | CEBM-2 | Limitations and comments |
| --- | --- | --- | --- | --- | --- | --- | --- | --- | --- | --- | --- | --- | --- | --- |
| 14 | Castellani et al | 2009 | P | Cohort | 109 |  | < 18 | 13-15 | HI and clinical symptoms (vomiting or LOC) and/or persisting HA, retrograde amnesia and vertigo, if > 4yrs | No CT or if S100B taken > 6hrs from trauma | No | 100 | 3 | SB: towards specific symptoms |
| 15 | Atabaki et al | 2008 | P | Observational cohort | 1000 | 188 | <21 | 13-15 | Closed HI and CT according to local guidelines | CT prior to referral | No | 100 | 3 | SB: towards high-risk MHT |
| 16 | Da Dalt et al | 2007 | P | Cohort | 1248 |  | <15 | Any | Blunt HI < 24h, normal mental status, no physical evidence of basilar, depressed or open fracture | Signs of depressed, basilar or open SF, no follow-up (14 triplets) | No | no data | 4 | SB: ICI only patients w. GCS<13 |
| 16 |  |  |  | Case-control 1:2 | 444* |  |  | 13-15 | As above and observed for < 2hrs and discharged from ED, vomiting before or at ED (cases) vs non-vomiting (controls) |  | Telephone interview within 10 days to 1-6 months after ED |  |  | VB: n of CTs not given |
| 17 | Da Dalt et al. | 2006 | P | Cohort | 3806 | 1396 | <16 | Any | Blunt HI < 24h | Admission >24h post injury, open injury, previous neurologic disorder, bleeding diathesis | Discharged from ED got follow-up telephone interview 10 days after trauma; hospital registry for re-admissions at end of study | 2.1 | 3 | VB: low CT-rate |
| 18 | Dunning et al | 2006 | P | Cohort | 22772 | 6235 | <16 | Any | Any patient with history or sign of injury to the head | Refusal to consent | Medical records and cross-checking with wider area for XR and CTs performed | 3.4 | 3 | SB: any GCS |
| 19 | Oman et al | 2006 | P | Cohort | 1666 |  | <19 | Any | Blunt HI, all with CT (=high-risk suspicion) based on physician's discretion | Imaging for other than head injury, eg. Penetrating trauma, infections, cerebellar injury | None stated | 5.0 | 2 | Subset of Nexus-II |
| 20 | Boran et al | 2006 | P | Cohort | 421 |  | <17 | 15 | No neurological deficits | Penetrating skull injury/gunshot, multiple trauma | All with linear SF and negative CT were observed for 24h and repeat-CT before discharge. | 100 | 3 | possible SB; VB not blinded |
| 21 | Muñoz-Sánchez et al | 2005 | R | Cross-sectional | 60 |  | <14 | 14-15 | Medium and high-risk, all SXR | Low risk patients | No | 100 | 4 | SB, VB: Focus on SXR and high-risk MHT |
| 22a | Palchak et al | 2003 | P | Cohort observational | 2043 |  | < 18 | Any | Nontrivial blunt HI w. history or symptoms consistent to with head trauma | Falls from ground level or run into stationary objects if only symptom was scalp laceration or abrasion, if CT performed before transfer from other hospital. | Telephone 1 week after ED or postal questionnaire. County morgue records and hospital trauma registry for non-responders (12%) | 62.2 | 3 | VB: not blinded |
| 22b |  | 2004 |  |  |  |  |  |  |  |  |  |  |  |  |
| 23 | Haydel et al. | 2003 | P | Cohort | 175 |  | 5-17y | 15 | HI with non-trivial mechanism < 24hr AND LOC (witnessed, reported or amnesia) and CT | GCS 12-13, irritability/agitation, if without LOC or refused CT | No | 100 | 3 | SB: towards high-risk MHT |
| 24 | Simon et al | 2001 | R | Cohort | 429 |  | <16 | 14-15 | Received CT due to "high-risk" mechanism. Known LOC. (High-risk mechanism: fall>20ft or 3x height of child, MVC or unrestrained infant in MVC, assault w blunt object, struck by car as pedestrian or biker, fall from vehicle struck by car. | None | Medical records | 100 | 4 | SB: towards high-risk MHT |
| 25 | Ratan et al | 2001 | R | Cohort | 400 |  | children | Any | Documented duration of unconsciousness (after HI) | None | No | no data | 4 | SB: many moderate-severe; VB: CT in 60%, no follow-up |
| 26 | Greenes and Schutzman | 2001 | P | Cohort | 422* |  | <2 | 15 | Only asymptomatic patients | Palpable depressions of skull | Medical records and telephone calls to all 2 weeks after ED visit | 18 | 3 | SB: asymptomatic |
| 27 | Klassen et al. | 2000 | R | Cohort | 1164 |  | <16 | 13-15 | Blunt HI < 24h, and definite LOC, amnesia or witnessed disorientation | No clear history of trauma, penetrating skull injury, unexplainable focal neurologic deficit, seizure prior to assessment, VP-shunt, pregnancy | Medical charts | 15 | 3 | SB: selected risk factors  VB: CT rate |
| 28 | Wang et al | 2000 | P | Cohort | 157 |  | <15 | 13-14* | Blunt trauma. Patients with paramedic transportation to trauma centre according to EMS criteria. GCS = field scores | Stab and gunshot wounds | No | no data | 4 | SB; VB: *pre-hospital symptoms |

| No | Study | Year | Design | | N | n < 2y | Age (y) | GCS score | Other inclusion criteria | Exclusion criteria | Follow-up | CT rate % | CEBM-2 | Limitations and comments |
| --- | --- | --- | --- | --- | --- | --- | --- | --- | --- | --- | --- | --- | --- | --- |
| 29 | Greenes et al | 1999 | P | Cohort | 608 | 608 | <2 | Any | Head trauma | None | Medical records and telephone calls to all patients 2weeks after initial ED visit | 31 | 3 | SB: any severity; VB: CT rate and non-blinding |
| 30 | Gruskin et al | 1999 | R | Cohort | 278 | 278 | <2 | Any | Discharge diagnoses from ED or hospital of either head injury, SF (linear, depressed,basilar), ICH (hematoma, cerebral contusion) or cerebral oedema | If only symptom is scalp or facial laceration, bleeding disorder, seizure disorder, neurological abnormality, VP-shunts | Medical records | 40 | 4 | SB towards severe HI; VB: SXR or CT |
| 31 | Lloyd et al | 1997 | P | Cohort | 883 | 193 | 0-16 | Any | HT and SXR and admission to hospital; and all with SF on SXR but discharged from ED | Referral from elsewhere and isolated facial injury | Telephone call if missed SF on SXR at 1st view (within 62h after injury or recall for review) | 18 | 3 | SB; VB. Focus on SF on SXR |
| 32 | Quayle et al | 1997 | P | Cohort | 321 | 135 | <18 | Any | Non-trivial head trauma | Penetrating injury to head, scalp laceration if pt >12 months old, scalp hematoma if patient > 24 months old. | Telephone 3-7 days after discharge from ED. | 98 | 3 | 7 without CT were well at follow-up |
| 33 | Shane et al | 1997 | R | Cohort | 102 | 102 | <13 months | 14-15 | Acute SF < 12h (either on SXR or CT) | SF from birth trauma | Medical records: clinical follow-up in 14/15 with ICI, 12 "by chance" | 31 | 4 | SB: only if CT or SXR show SF; VB - few with follow-up and CT |
| 34 | Loroni et al | 1996 | P | Cohort | 942 | 129 | <15 | Any | Head trauma | None | 6 months neurological evaluation for complicated cases | 4.6 | 4 | VB - possible |
| 35 | Schunk et al | 1996 | R | Cohort | 313 | 97 | <19 | 15 | Head trauma and CT | Altered level of consciousness (GCS <15), known depressed SF, bleeding diathesis, developmental delay | No | 100 | 3 | SB: only those who had a CT = high-risk MHT |
| 36 | Ramundo et al | 1995 | P | Cohort | 300 | 37 | 2-18 | Any | Closed head trauma and CT (at physicians discretion) | None | No | 100 | 3 | SB: only those who had a CT = high-risk MHT |
| 37 | Davis et al | 1994 | R | Cohort | 185 |  | 2-17 | 15 | Closed HT with observed LOC, initial GCS 15 in ED. If > 6y rs non-observed HT and amnesia were included | None | No | 100 | 3 | SB: moderate risk of MHT |
| 38 | Mitchell et al | 1994 | R | Cohort | 401 |  | <17 | 13-15 | Isolated minor head injury | No other major injuries necessitating hospital admission | Telephone interview (34%) 3-6 months for PCS | 54 | 3 | 2 readmissions (leptomeningeal cyst after linear SF and brachial plexus injury) |
| 39 | Hahn and McLone | 1993 | P | Cohort | 791 |  | <16 | 13-15 | HT and admission to hospital (admission if either LOC, neurological deficits, HA, vomiting or nausea, SF on SXR or abnormal CT, suspected child abuse, no reliable caretaker) | None | Out-patient clinics after discharged. No time frame given. | 100 | 3 | SB: towards more severe injury and specific risk factors |
| 40 | Dietrich et al | 1993 | P | Cohort | 322 | 71 | <21 | Any | All with CT after HT (according to physicians discretion) | None | No | 100 | 3 | SB: towards more severe injury |
| 41 | Chan et al | 1990 | R | Case-series | 12072 |  | <16 | Any | HT < 48h admitted to surgical ward, all with SXR | None | Medical records | no data | 3 | SB: towards more severe HT – admission to surgical ward |
| 42 | Chan et al | 1990 | P | Cohort validation | 418 |  | 11-15 | Any | HT and admitted to neurosurgical ward (many different admission criteria) | Referred patients from other hospitals for management of HT | 3 months GOS/post mortem analysis | no data | 3 | SB: towards more severe injury; VB CT performed when clinically indicated. |
| 43 | Munivenkatappa  et al | 2013 | R | Cohort | 133 |  | < 13 | 13-15 | HT, MTBI, admitted to tertiary level neuro/trauma centre | None | No | 100 | 3 | Possible SB to high-risk. All had CT. |
| 44 | Levi et al | 1991 | P | Cohort | 653 |  | <15 | Any | Acute head trauma, within 48h post injury | CT normal and discharged or no CT or neurological evaluation | Medical records - GOS (time unknown) | 100 | 3 | Selected high-risk patients, uncertain follow-up |

| No | Study | Year | Design | | N | n < 2y | Age (year) | GCS score | Other inclusion criteria | Exclusion criteria | Follow-up | CT rate % | CEBM-2 | Limitations and comments |
| --- | --- | --- | --- | --- | --- | --- | --- | --- | --- | --- | --- | --- | --- | --- |
| 45 | Mandera et al | 2000 | R | Cohort | 166 |  | 0-17 | 13-15 | MHT, children. Admitted to Neurosurgery department in 1985-95. | Not stated, although 375 cases available, 166 included. | GOS at discharge | 100 | 4 | SB: towards very high risk patients. All chosen to CT. 6 patients normal CT, delayed hematoma |
| 46 | Melo et al | 2008 | P | Cohort | 1888 | 437 | 0-19 | 13-15 | MHT. Admitted to ED trauma centre. | None | None | 38.8 | 3 | VB – no follow-up. |
| 47 | Murgio et al | 2001 | P | Cohort | 4690 | 3616 | 0-15 | Any | HT. | None | 2 months (clinic or phone) according to GOS. | 14.3 | 3 | SB – all GCS included. |
| 48 | Schonfeld et al | 2013 | P | Cohort observational | 1381 | 509 | <18 | 14-15 | Blunt HT, admission within 24h of injury | Trivial injury mechanism and no signs of trauma, neurologic comorbidities (shunt, tumour), bleeding disorders, if CT was done before admission to ED. | No | 19.7 | 3 | PECARN evaluation and “observation before CT”  VB: no follow-up |
| 49 | Maier et al | 2003 | R | Cohort | 437 |  | 3 mo -15y | Any | Craniocerebral trauma admitted to ED | None | All MHI with positive CT had a telephone interview 30-76 months post trauma | 56 | 3 | SB: 22 patients <GCS 8 (5%); VB: varying follow-up time |
| 50 | Garcia Garcia et al | 2009 | P | Observational cohort | 1070 | 476 | < 18 | 13-15 | All HT < 72h, treated in paediatric ER. | Direct ICU, poly trauma, coagulopathy | If discharged from ER – telephone contact between 48-72h | 9 | 3 | SB: HT up to 72h post-injury |

R=retrospective, P = prospective study. MHT = minor head trauma, HT = head trauma, MTBI = mild traumatic brain injury, ED = emergency department, ICU = intensive care unit, GCS = Glasgow coma scale, GOS = Glasgow outcome score, HA=headache, LOC = loss of consciousness, CP = cerebral palsy, SBP = systolic blood pressure, ICD = international classification of diseases, ICH = intracerebral haematoma, ICI = intracranial injury, ICU = intensive care unit, mo = months, PCS = postconcussion syndrome, SF = skull fracture, XR= X-ray, SXR= skull X-ray, CT = computed tomography, SB = selection bias, VB = verification bias.
