# Supplementary material for: Scandinavian guidelines for initial management of minor and moderate head trauma in children
Source: BMC Med. 2016 Feb 18;14:33. doi: 10.1186/s12916-016-0574-x (PMC4758024; doi:10.1186/s12916-016-0574-x)
Supplement: Additional file 2: Table S2. — Evidentiary table of studies referring to clinical question 2: “Which paediatric patients with (non-severe) head trauma need in-hospital observation and/or repeat head CT?” (DOCX 32 kb) [file 12916_2016_574_MOESM2_ESM.docx]

Table S2. Evidentiary table of studies referring to the clinical question 2: “*Which paediatric patients with head trauma need in-hospital observation and/or repeat head CT?”*

| No | Study | Year | Design | | n | Age | GCS score | Other inclusion criteria | Exclusion criteria | Follow-up | Relevant findings | Limitations and comments | CEBM-2 |
| --- | --- | --- | --- | --- | --- | --- | --- | --- | --- | --- | --- | --- | --- |
| 1 | Aziz et al. | 2013 | R | Cohort | 191 | 2 – 18 | any | Blunt TBI, abnormal initial CT | Transfers from other hospital or emergent craniotomy before 2^nd^ CT or no 2^nd^ CT and discharged home same day | No | 184 routine 2nd CTs, 32 of these progressed, 3 to NS (sTBI initially). 7 acute 2nd CTs, 6 progressed, 3 to NS (2 mTBI, 1 sTBI).  No change in management in patients with mild and moderate TBI, on the basis of routine CT and normal neurological exam. There is no substitute to neurological examination in paediatric head trauma patients. | No routine CT if mild or moderate HI and normal neurology | 3 |
| 2 | Dawson et al. | 2012 | R | Cohort | 507 | <19 | 13-15 | At least 1 CT <12h after injury and normal neuro exam throughout the hospitalization | 1st CT > 12h after injury, 1st CT not read by institution, require immediate NS, EDH or depressed SF on CT. | No | No change in management despite routine CT for MHI children, when neurology is the same or improved.  Impact on length of stay >23h: abnormal initial CT (OR 2.81), initial CT is abnormal AND repeat CT is ordered (OR 5.21) | No routine repeat CT in MHI, unless worsening symptoms | 4 |
| 3 | Schnellinger et al. | 2010 | R | Case-series | 47 | <18 | Any | CT verified ICI (according to ICD-9), 2 CTs performed at institution | Transfer from other hospital after ICI confirmed, not a 2nd CT, bleeding or metabolic disorder, if intervention against ICP before 2nd CT (NS, intubation, mannitol) | No | 5 of 47 underwent NS following 2nd CT, 1 of these was unplanned after initial CT due to worsening headache (EDH), the other 4 were infants with NAI and mixed ICIs (no symptoms of increased ICP). The rest (42) did not have any change in management after 2nd routine CT, and no symptoms of increased ICP.  Conclusion: Serial brain imaging may not be required for all children with ICI. | Serial CT after TBI, not for all children.  Repeat CT had a consequence if worsening symptoms or NAI with ICI | 4 |
| 4 | Hollingworth et al. | 2007 | R | Cohort | 521 | <15 | Any | Acute blunt HT, 2 CTs (2^nd^ within 24 h after ED) | Transfer from other hospital, 1st CT after 6 h of ED, NS before 2nd CT, dead in ED, penetrating injury | Medical records | Mild 50%, Moderate 10%, Severe 40%.  Mild: 3 to NS due to worse 2nd CT, all with clinical deterioration before 2nd CT (1 EDH new, 1 EDH progress, 1 oedema). Moderate-severe: NS on 4 despite stable repeat CT. 7 died all severe head injury initially.  Normal initial CT in MHT lower the risk of progression on repeat-CT.  Moderate or severe HI, presence of any intraparenchymal finding on first CT and coagulation disorder (measured by PTT, INR and prothrombin) increase the risk of progression on repeat CT or NS. | Low-risk progression on routine repeat CT:  -normal initial CT in MHT.    Higher risk of progression if:  -coagulopathy  -ICH/contusion  -Moderate to severe HT | 3 |
| 5 | Durham et al. | 2006 | R | Cohort observational | 268 | <18 | - | Initial CT for suspected intracranial injury on admission. Repeat CT within 24h. | Transfers for tertiary care, NS operation after initial CT scan (not ICP monitors or EVD) | No | None with normal initial CT scan progressed on 2nd CT. Signs of increased likelihood of delayed NS intervention in the high risk lesion group (EDH, SDH, oedema, IPH). 8 died (whole population).  High-risk group is recommended repeat-CT, while low-risk lesions (SAH, IVH, DAI) and isolated SF without sign of clinical deterioration, repeat CT will be less likely to alter management. | Repeat CT for high-risk lesions (EDH, SDH, oedema, ICH) | 3 |

| No | Study | Year | Design | | n | Age | GCS score | Other inclusion criteria | Exclusion criteria | Follow-up | Relevant findings | Limitations and comments | CEBM-2 |
| --- | --- | --- | --- | --- | --- | --- | --- | --- | --- | --- | --- | --- | --- |
| 6 | Givner et al. | 2002 | R | Case-series | 104 | <18 | Any | Blunt HT or penetrating head injury. TBI on initial CT. 2nd CT within 24-48h. | Surgery before 2nd CT, negative 2nd CT and suspicious 1st, no medical records access. | Medical records | 23 of 50 (46%) with progression of 2nd CT were treated more intensely (7 NS, 2 ICP), while 4 of 54 (7.4%) without progression on 2nd CT had change in management (2 NS). About 11% had 2nd CT due to clinical deterioration.  Children with ICI identified on initial CT should undergo a repeat CT scan 24h after injury, especially if 3 or more ICI, mass effect, IVH or EDH. | Repeat CT after 24h for high-risk lesions:  -multiple ICI  -mass effect  -IVH  -EDH | 4 |
| 7 | da Silva et al. | 2008 | R | Cohort observational | 63 | <15 | 3-12 | Moderate to severe TBI surviving the first 24hs after admission. At least 1 repeat CT in the first 48h after initial CT | Penetrating HT, if CT performed >12h after admission | No | 22 patients SHI, 4 of these died subsequently.  22% repeat CTs due to clinical worsening, 78% for routine follow-up. 10% of the routine CT showed progression, none needed NS.  Conclusion: Improvement of GCS after initial CT does not require routine CT within 48h. | moderate-severe TBI  No repeat CT if clinical improvement within 48h. | 3 |
|  |  |  |  |  |  |  |  |  |  |  |  |  |  |
| 8 | Holmes et al. | 2011 | P | Cohort observational | 13543 | <18 | 14-15 | Blunt HT, nontrivial. Initial CT in ED is normal. | GCS <14 in ED, initial CT+ findings, coagulopathy, shunt | Phone <1 week , mail survey, medical records, county morgue | NPV for NS of a normal initial CT in patient w GCS 15 = 100% (95% CI: 99.97-100%). *197 of those discharged from ED with normal initial CT, had a repeat CT/MRI. 5 of these were abnormal, none needed NS. Children with blunt head trauma and initial GCS scores of 14-15 and normal initial CT are at very low risk for subsequent traumatic findings on neuroimaging and extremely low risk of needing NS.  Hospitalization of these children for neurologic observation is generally unnecessary. | Minor blunt head trauma and ED GCS of 14-15  can  generally be discharged after normal head CT | 3 |
| 9 | Roddy et al. | 1998 | R | Case series | 62 | <16 | 15 | MHT (LOC or amnesia), GCS 15, normal neurology and normal head CT | Associated injuries requiring admission | no | 9 with prolonged stay in hospital due to nausea/delay in CT reading, 5 due to other reasons not related to HT symptoms.  Mandatory admission for children with head trauma having a normal initial CT and normal neurology is not warranted. | Minor closed head injury  No routine hospitalization | 4 |
| 10 | Davis et al. | 1995 | R | Case-series | 400 | <18 | 13-15 | Minor HT with or without LOC AND all with no intracranial bleeding on initial CT | Hypoxia, penetrating injury, depressed fractures requiring NS or initial CT >24h after trauma | 1. medical records  2. CHARS system  3. death index | 4 of 110 (3.6%) returned after direct discharge to home.  3 were re-scanned, 1 needed NS due to SDH on the 5th day = Coumadin patient, 1 ICH on return on day 3 but conservatively treated. The rest concussion.  In otherwise stable patients, a normal cranial CT scan can identify patients to be safely discharged from the emergency department, and would be more cost-effective than 1-2 days of hospital observation. | Coumadin patients should not be early discharged from ED, despite normal CT. | 4 |
| 11 | Spencer et al. | 2003 | R | Cohort | 197 | <14 | 15 | Blunt HT, non-focal neurology exam, and normal head CT. | None | No | 123 discharged after 1 day, 71had a longer stay due to concomitant injuries or abuse. None had change in management due to their head injury/concussion. 5 patients with persistent symptoms. No delayed complications.  If normal CT, normal focal neurology, GCS 15, there is no need for mandatory in-hospital observation, unless other injuries require this. | Negative CT after mild HT = no admission | 4 |
| 12 | Holsti et al. | 2005 | R | Cohort | 284 | <19 | 9-15 | Closed HT observed in the OU in ED. | Need for immediate in-hospitalization. | No | Inclusion: only 1 patient with GCS 9-11, about 84% GCS 15.  13 patients from OU needed unplanned in-hospital admission (cerebral venous thrombosis, persistent CSF leakage, decreased level of consciousness.)  Conclusion: the presence of basilar skull fracture, head laceration and need for ED iv fluids were associated with increased risk of UIA. OU admission is an efficient management setting for children with stable intracranial pathology, skull fractures and concussion. | Observation unit after head CT, lower hospital admission  OU = 24h observation  Skull base fractures may need admission | 4 |

R = retrospective, P = prospective study. GCS = Glasgow coma scale, LOC = loss of consciousness. CT = computed tomography, MRI = magnetic resonance imaging, ICI = intracranial injury, NS = neurosurgery. HT= head trauma, MHT = minor head trauma, SHI= severe head injury, TBI = traumatic brain injury. SF = skull fracture, ICH = intracerebral haematoma, ICI = intracranial injury, IVH = intraventricular haemorrhage, EDH = epidural haematoma, SDH = subdural haematoma, ICP = intracranial pressure, EVD = external ventricular drainage. ED = emergency department, OU = observation unit, UIA = unintended admission.
