# Supplementary material for: Scandinavian guidelines for initial management of minor and moderate head trauma in children
Source: BMC Med. 2016 Feb 18;14:33. doi: 10.1186/s12916-016-0574-x (PMC4758024; doi:10.1186/s12916-016-0574-x)
Supplement: Additional file 3: Table S3. — QUADAS-2 and CEBM-2 evaluation for papers regarding clinical question 1: “Which children with (non-severe) head trauma should be CT scanned, and which can be discharged?” (DOCX 30 kb) [file 12916_2016_574_MOESM3_ESM.docx]

|  |  |  | Risk of bias | | | | Applicability | | |
| --- | --- | --- | --- | --- | --- | --- | --- | --- | --- |
| Study No | Author | CEBM-2 | 1A. Patient selection | 2A. Index test | 3A. Reference standard | 4. Flow and timing | 1B. Patient selection | 2B. Index test | 3B. Reference standard |
| 1 | Nigrovic | 2 | ☺ | ☺ | ☹ | ☹ | ☺ | ☺ | ☺ |
| 2 | Xiao | 3 | ☹ | ☹ | ☹ | ☺ | ☹ | ☺ | ☺ |
| 3 | Bouvier | 3 | ☺ | ? | ? | ☹ | ☺ | ☺ | ☺ |
| 4 | Bressan | 3 | ☹ | ☹ | ☹ | ☹ | ☺ | ☺ | ☺ |
| 5 | Ng | 4 | ☺ | ☺ | ☹ | ☹ | ☺ | ☺ | ☹ |
| 6 | Fabbri | 3 | ☹ | ☺ | ☹ | ☹ | ☺ | ☺ | ☹ |
| 7 | Crowe | 3 | ☺ | ☹ | ☺ | ☹ | ? | ☺ | ? |
| 8 | Hallén | 3 | ☺ | ☺ | ☹ | ☹ | ☺ | ☺ | ☹ |
| 9 | Osmond | 3 | ☹ | ☹ | ☹ | ☹ | ☹ | ☺ | ☺ |
| 10 | Bechtel | 3 | ? | ☹ | ☺ | ☺ | ☹ | ☺ | ☺ |
| 11 | Klemetti | 4 | ☺ | ☹ | ☹ | ☹ | ☺ | ☹ | ☺ |
| 12 a/b | Kupperman/Nigrovic | 2 / 3 | ☺ | ☺ | ☺ | ☹ | ☺ | ☺ | ☺ |
| 13 | Guzel | 4 | ? | ☹ | ☹ | ☹ | ☺ | ☺ | ☹ |
| 14 | Castellani | 3 | ☹ | ☺ | ☺ | ☺ | ☹ | ☺ | ☺ |
| 15 | Atabaki | 3 | ☹ | ☺ | ☺ | ☺ | ☹ | ☺ | ☺ |
| 16 | Da Dalt | 4 | ☹ | ☺ | ☹ | ☹ | ☹ | ☺ | ☹ |
| 17 | Da Dalt | 3 | ☺ | ☺ | ☹ | ☹ | ☺ | ☺ | ☺ |
| 18 | Dunning | 3 | ☺ | ☺ | ☹ | ☹ | ☺ | ☺ | ☹ |
| 19 | Oman | 2 | ☺ | ☺ | ☺ | ☺ | ☺ | ☺ | ☺ |
| 20 | Boran | 3 | ☹ | ☺ | ☹ | ☺ | ☺ | ☺ | ☺ |
| 21 | Munoz-Sanchez | 4 | ☹ | ☺ | ☹ | ☹ | ☹ | ☹ | ☹ |
| 22 a/b | Palchak | 3 | ☺ | ☺ | ☹ | ☹ | ☺ | ☺ | ☺ |
| 23 | Haydel | 3 | ☹ | ☺ | ☺ | ☺ | ☹ | ☺ | ☺ |
| 24 | Simon | 4 | ☹ | ☹ | ☹ | ☺ | ☹ | ☹ | ☺ |
| 25 | Ratan | 4 | ☹ | ☺ | ☹ | ☹ | ☹ | ☹ | ☹ |
| 26 | Greenes | 3 | ☺ | ☺ | ☹ | ☹ | ☺ | ☺ | ☹ |

Additional file 3, Table S3. QUADAS-2 and CEBM-2 evaluation for papers regarding the clinical question 1: *“Which paediatric patients with head trauma need a head CT and which may be directly discharged?*

| 27 | Klassen | 3 | ☺ | ☹ | ☹ | ☹ | ☺ | ☺ | ☹ |
| --- | --- | --- | --- | --- | --- | --- | --- | --- | --- |
| 28 | Wang | 4 | ☺ | ☹ | ☹ | ☹ | ☹ | ☹ | ☹ |
| 29 | Greenes | 3 | ☺ | ☺ | ☹ | ☹ | ☹ | ☺ | ☺ |
| 30 | Gruskin | 4 | ☺ | ☹ | ☹ | ☹ | ☺ | ☺ | ☺ |
| 31 | Lloyd | 3 | ☹ | ☺ | ☹ | ☹ | ☺ | ☹ | ☹ |
| 32 | Quale | 3 | ☺ | ☺ | ☹ | ☹ | ☹ | ☺ | ☺ |
| 33 | Shane | 4 | ☹ | ☹ | ☹ | ☹ | ☹ | ☺ | ☹ |
| 34 | Loroni | 4 | ☺ | ☺ | ☹ | ☹ | ☹ | ☹ | ☺ |
| 35 | Schunk | 3 | ☹ | ☹ | ☹ | ☹ | ☺ | ☺ | ☺ |
| 36 | Ramundo | 3 | ☹ | ☹ | ☹ | ☹ | ☹ | ☺ | ☺ |
| 37 | Davis | 3 | ☹ | ☺ | ☺ | ☺ | ☹ | ☺ | ☺ |
| 38 | Mitchell | 3 | ☺ | ☹ | ☹ | ☹ | ☺ | ☹ | ☹ |
| 39 | Hahn | 3 | ☹ | ☹ | ☹ | ☹ | ☺ | ☺ | ☹ |
| 40 | Dietrich | 3 | ☹ | ☹ | ☺ | ☺ | ☹ | ☹ | ☺ |
| 41 | Chan | 3 | ☺ | ☹ | ☹ | ☹ | ☹ | ☺ | ☹ |
| 42 | Chan | 3 | ☺ | ☹ | ☹ | ☹ | ☹ | ☹ | ☺ |
| 43 | Munivenkatappa | 3 | ☺ | ☺ | ☹ | ☺ | ☹ | ☺ | ☺ |
| 44 | Levi | 3 | ☹ | ☹ | ☺ | ☺ | ☹ | ☹ | ☺ |
| 45 | Mandera | 4 | ☹ | ☺ | ☹ | ☺ | ☹ | ☹ | ☺ |
| 46 | Melo | 3 | ☺ | ☺ | ? | ☹ | ☺ | ☺ | ☹ |
| 47 | Murgio | 3 | ☺ | ☺ | ☹ | ☹ | ☹ | ☺ | ☹ |
| 48 | Schonfeld | 3 | ☺ | ☺ | ☹ | ☹ | ☺ | ☹ | ☺ |
| 49 | Maier | 3 | ☺ | ☺ | ☹ | ☹ | ☺ | ☹ | ☹ |
| 50 | Garcia | 3 | ☺ | ☺ | ☹ | ☹ | ☺ | ☺ | ☹ |

☺ low concern ☹ high concern ? unknown concern
