# Supplementary material for: Scandinavian guidelines for initial management of minor and moderate head trauma in children
Source: BMC Med. 2016 Feb 18;14:33. doi: 10.1186/s12916-016-0574-x (PMC4758024; doi:10.1186/s12916-016-0574-x)
Supplement: Additional file 4: Table S4. — QUADAS-2 and CEBM-2 evaluation for papers regarding clinical question 2: “Which children with (non-severe) head trauma need a repeat CT and/or in-hospital admission?” (DOCX 21 kb) [file 12916_2016_574_MOESM4_ESM.docx]

|  |  |  | Risk of bias | | | | Applicability | | |
| --- | --- | --- | --- | --- | --- | --- | --- | --- | --- |
| Study No | Author | CEBM-2 | 1A. Patient selection | 2A. Index test | 3A. Reference standard | 4. Flow and timing | 1B. Patient selection | 2B. Index test | 3B. Reference standard |
| 1 | Aziz | 3 | ☹ | ☹ | ☺ | ☺ | ☺ | ☺ | ☺ |
| 2 | Dawson | 4 | ☹ | ☹ | ☹ | ☹ | ☺ | ☺ | ☹ |
| 3 | Schnellinger | 4 | ☹ | ☺ | ☹ | ☺ | ☹ | ☹ | ☺ |
| 4 | Hollingworth | 3 | ☹ | ☹ | ☺ | ☺ | ☺ | ☺ | ☺ |
| 5 | Durham | 3 | ☺ | ☺ | ☹ | ☺ | ☺ | ☺ | ☺ |
| 6 | Givner | 3 | ☹ | ☺ | ☹ | ☺ | ☺ | ☺ | ☺ |
| 7 | Da Silva | 3 | ☺ | ☹ | ☹ | ☺ | ☺ | ☺ | ☺ |
| 8 | Holmes | 3 | ☺ | ☹ | ☺ | ☹ | ☺ | ☹ | ☺ |
| 9 | Roddy | 4 | ☺ | ☹ | ☹ | ☹ | ☺ | ☹ | ☹ |
| 10 | Davis | 4 | ☺ | ☹ | ☹ | ☹ | ☹ | ☹ | ☺ |
| 11 | Spencer | 4 | ☺ | ☹ | ☹ | ☺ | ☺ | ☹ | ☺ |
| 12 | Holsti | 4 | ☹ | ☺ | ☹ | ☹ | ☺ | ☺ | ☺ |

Additional file 4, Table S4: QUADAS-2 and CEBM-2 evaluation for papers regarding the clinical question 2: “*Which paediatric patients with head trauma need in-hospital observation and/or repeat head CT?”*

☺ low concern ☹ high concern ? unknown concern
