# Supplementary material for: Scandinavian guidelines for initial management of minor and moderate head trauma in children
Source: BMC Med. 2016 Feb 18;14:33. doi: 10.1186/s12916-016-0574-x (PMC4758024; doi:10.1186/s12916-016-0574-x)
Supplement: Additional file 5: Table S5. — Clinical predictors for pathology on head CT, intracranial injury and neurosurgery after minor and moderate head trauma in children. (DOCX 285 kb) [file 12916_2016_574_MOESM5_ESM.docx]

**Additional file 5, Table S5: Clinical predictors for pathology on head CT, intracranial injury and neurosurgery after minor and moderate head trauma in children**

Predictive factors are derived from studies referring to the clinical question “*“Which paediatric patients with head trauma need a head CT and which may be directly discharged?”*

ICI = intracranial injury, NS = neurosurgery, ciTBI = clinically important traumatic brain injury (for definition see manuscript text) PLR=positive Likelihood Ratio, NLR=Negative Likelihood Ratio, Prev = prevalence of risk factor for positive CT, ICI or NS Calc = calculated from the data given in the study. Assumpt = Assumption made based on the given data.

The different colours in the tables below refer to the studies directly influencing on the final categories in the guidelines (see flow chart).

1. Age (years unless otherwise stated, mo = months)
2. Gender
3. GCS score < 15
4. Severity of head injury
5. Altered mental status (composite definition) incl. irritability
6. Abnormal behaviour (according to guardian)
7. Drowsiness/lethargy/irritability/confusion
8. Amnesia
9. Vomiting
10. Headache
11. Loss of consciousness (LOC)
12. Focal neurology
13. Seizures
14. Skull fractures
15. Scalp hematoma and location
16. Scalp laceration
17. Deterioration
18. Signs of increased intracranial pressure (ICP)
19. Helmet use
20. Intoxication
21. Extracranial injury
22. Vertigo
23. Other symptoms
24. Falls
25. Traffic accidents (MVA=motor vehicle accident)
26. Assault
27. Struck by object/Sports trauma
28. Coagulopathy
29. Shunts
30. S100B

| **1. Age** | | | |  |  |  |  |  |  |  |  |  |  |
| --- | --- | --- | --- | --- | --- | --- | --- | --- | --- | --- | --- | --- | --- |
| **Risk factor** | **Study** | **Year** | **Age** | **Prev risk factor** | **PLR CT** | **NLR CT** | **Prev CT** | **PLR ICI** | **NLR ICI** | **Prev ICI** | **PLR NS** | **NLR NS** | **Prev NS** |
| Age 0-2 mo | Greenes | 1999 | <2 | 15.1% |  |  |  | 2.9 | 0.7 | 2.0% |  |  |  |
| Age 3-11 mo | Greenes | 1999 | <2 | 36.8% |  |  |  | 1.2 | 0.9 | 2.1% |  |  |  |
| Age 12-23 mo | Greenes | 1999 | <2 | 48.0% |  |  |  | 0.3 | 1.7 | 0.8% |  |  |  |
| Age 0-11 mo vs 12-23 mo (calc) | Greenes | 1999 | <2 | 52.0% |  |  |  | 1.7 | 0.3 | 4.1% |  |  |  |
| Age <1 (vs 1-16) | Boran | 2006 | <17 | 7.4% |  |  |  | 0.7 | 1.0 | 0.5% |  |  |  |
| Age <2 vs 2-14 | Bressan S (a) | 2012 | < 15 | 45.1% |  |  |  | 2.2 | 0.0 | 0.7% |  |  | 0.0% |
| Age <2 vs 2-14 | Bressan S (b) | 2012 | < 15 | 45.5% |  |  |  | 0.4 | 1.5 | 0.3% |  |  | 0.0% |
| Age <2 | Atabaki | 2008 | <21 | 18.8% |  |  |  | 2.4 | 0.7 | 2.7% |  |  |  |
| Age 0-2 (GCS any) | Murgio | 2001 | 0-15 | 15.9% | 0.6 | 1.1 | 3.7% |  |  |  |  |  |  |
| Age 0-2 (mild+moderate) | Murgio | 2001 | 0-15 | 17.2% | 0.8 | 1.1 | 4.0% |  |  |  |  |  |  |
| Age <2 vs 2-21 (calc) | Dietrich | 1993 | 2-21 | 21.9% | 0.7 | 1.1 | 3.7% |  |  |  |  |  |  |
| Age 0-3 vs 4-16 (calc) | Boran | 2006 | <17 | 64.1% |  |  |  | 0.7 | 1.7 | 3.8% |  |  |  |
| Age 1-3 | Boran | 2006 | <17 | 42.0% |  |  |  | 0.9 | 1.1 | 3.3% |  |  |  |
| Age 3-9 (GCS any) | Murgio | 2001 | 0-15 | 41.0% | 1.7 | 0.6 | 19.8% |  |  |  |  |  |  |
| Age 3-9 (mild+moderate) | Murgio | 2001 | 0-15 | 40% | 1.6 | 0.7 | 15.8% |  |  |  |  |  |  |
| Age 4-6 | Boran | 2006 | <17 | 32.8% |  |  |  | 1.5 | 0.8 | 4.0% |  |  |  |
| Age 2-7 vs 8-17 (calc) | Davis | 1994 | 2 - 17 | 23.8% |  |  |  | 0.9 | 1.0 | 1.8% |  |  |  |
| Age 5-10 vs age 11-17 | Haydel | 2003 | 5-17 | 30.3% |  |  |  | 1.5 | 0.8 | 3.4% |  |  |  |
| Age 7-9 | Boran | 2006 | <17 | 13.3% |  |  |  | 0.6 | 1.1 | 0.7% |  |  |  |
| Age 8-12 | Davis | 1994 | 2 - 17 | 31.0% |  |  |  | 0.9 | 1.0 | 2.4% |  |  |  |
| Age 10-15 (GCS any) | Murgio | 2001 | 0-15 | 43.1% | 0.7 | 1.3 | 11.6% |  |  |  |  |  |  |
| Age 10-15 (mild+moderate) | Murgio | 2001 | 0-15 | 41.0% | 0.6 | 1.3 | 8.8% |  |  |  |  |  |  |
| Age 10-16 | Boran | 2006 | <17 | 4.5% |  |  |  | 0.6 | 1.0 | 0.2% |  |  |  |
| Age 13-17 | Davis | 1994 | 2 -17 | 45.2% |  |  |  | 0.8 | 1.2 | 3.0% |  |  |  |
| Age <14 vs >14 | Munoz-Sanchez | 2005 | <14 | 38.5% |  |  |  | 0.2 | 2.1 | 3.2% |  |  |  |

| **2. Gender** | | | |  |  |  |  |  |  |  |  |  |  |
| --- | --- | --- | --- | --- | --- | --- | --- | --- | --- | --- | --- | --- | --- |
| **Risk factor** | **Study** | **Year** | **Age** | **Prev risk factor** | **PLR CT** | **NLR CT** | **Prev CT** | **PLR ICI** | **NLR ICI** | **Prev ICI** | **PLR NS** | **NLR NS** | **Prev NS** |
| Gender (male) | Guzel | 2009 | <17 | 24.3% | 3.0 | 0.5 | 4.7% |  |  |  |  |  |  |
| Gender (male) | Munivenkatappa | 2013 | <13 | 66.2% | 1.0 | 1.0 | 34.6% |  |  |  |  |  |  |
| Sex (males) | Fabbri | 2011 | < 11 | 63.0% |  |  |  | 1.4 | 0.3 | 0.7% |  |  |  |
| Gender (male) | Atabaki | 2008 | <21 | 64.1% |  |  |  | 1.0 | 1.1 | 4.0% |  |  |  |
| Male | Oman | 2006 | 0-18 | 64.4% |  |  |  | 1.0 | 1.0 | 5.2% |  |  |  |
| Male | Boran | 2006 | <17 | 56.8% |  |  |  | 1.1 | 0.9 | 5.5% |  |  |  |
| Male | Klassen | 2000 | <16 | 64.1% |  |  |  | 0.9 | 1.3 | 2.8% |  |  |  |

| **3. GCS score < 15 (in ED) vs GCS 15 unless otherwise stated** | | | |  |  |  |  |  |  |  |  |  |  |
| --- | --- | --- | --- | --- | --- | --- | --- | --- | --- | --- | --- | --- | --- |
| **Risk factor** | **Study** | **Year** | **Age (y)** | **Prev risk factor** | **PLR CT** | **NLR CT** | **Prev CT** | **PLR ICI** | **NLR ICI** | **Prev ICI** | **PLR NS** | **NLR NS** | **Prev NS** |
| **If AGE < 4y** |  |  |  |  |  |  |  |  |  |  |  |  |  |
| GCS <15 if age <1y | Dunning | 2006 | <16 | 0.4% |  |  |  | 9.0 | 1.0 | 0.0% |  |  |  |
| GCS <15 | Oman | 2006 | 0-3 | 44.7% |  |  |  | 0.4 | 1.8 | 6.1% |  |  |  |
| GCS ≤ 14 | Dietrich | 1993 | < 2 | 19.7% | 0.8 | 1.0 | 2.8% | 1.7 | 0.8 | 1.4% |  |  |  |
| PCS <15 | Ramundo | 1995 | < 2 | 24.3% | 2.9 | 0.6 | 10.8% |  |  |  |  |  |  |
| Depressed level of consciousness | Gruskin | 1999 | < 2 | 5.0% | 3.3 | 0.9 | 2.2% |  |  |  |  |  |  |
| **If AGE = any** |  |  |  |  |  |  |  |  |  |  |  |  |  |
| GCS <15 | Hallen | 2010 | < 18 | 11.7% | 0.7 | 3.8 | 3.6% |  |  |  |  |  |  |
| PCS <15 | Ramundo | 1995 | 2-18 | 37.1% | 2.6 | 0.3 | 12.7% |  |  |  |  |  |  |
| PCS <15 | Ramundo | 1995 | < 2 | 24.3% | 2.9 | 0.6 | 10.8% |  |  |  |  |  |  |
| GCS ≤ 14 | Dietrich | 1993 | < 2 | 19.7% | 0.8 | 1.0 | 2.8% | 1.7 | 0.8 | 1.4% |  |  |  |
| GCS ≤ 14 | Dietrich | 1993 | 2-21 | 30.4% | 0.8 | 1.6 | 13.8% | 1.4 | 0.6 | 10.7% |  |  |  |
| GCS <15 if age <1y | Dunning | 2006 | <16 | 0.4% |  |  |  | 9.0 | 1.0 | 0.0% |  |  |  |
| Abnormal GCS | Fabbri | 2011 | < 11 | 13.7% |  |  |  | 38.5 | 0.5 | 0.4% |  |  |  |
| GCS < 15 | Oman | 2006 | 0-18 | 22.2% |  |  |  | 3.8 | 0.4 | 5.7% |  |  |  |
| GCS<15 | Chan | 1990 | 11-15 | 5.0% |  |  |  | 5.2 | 0.8 | 0.7% | 0.1 | 0.8 | 0.72% |
| *GCS 9-14 (calc)* | Chan | 1990 | 11-15 | 3.6% |  |  |  | 0.0 | 1.0 | 0.0% |  |  |  |
| Impaired consciousness | Chan | 1990 | Any | 2.9% |  |  |  | 43.9 | 0.2 | 1.1% |  |  |  |
| **GCS 14 only** |  |  |  |  |  |  |  |  |  |  |  |  |  |
| GCS 14 (ciTBI as ICI) | Kupperman | 2009 | 2-17 | 2.8% |  |  |  | 10.3 | 0.8 | 0.2% |  |  |  |
| GCS 14 (incl LOC) | Simon | 2001 | <16 | 12.3% |  |  |  | 0.5 | 1.3 | 2.6% |  |  |  |
| GCS 14 | Munoz-Sanchez | 2005 | <14 | 6.7% |  |  |  | 3.7 | 0.8 | 1.7% |  |  | 3.3% |
| GCS 14 (calc) | Hahn | 1993 | <16 | 18.4% |  |  |  | 1.3 | 0.9 | 1.8% |  |  |  |
| GCS 14 (calc) | Mandera | 2000 | 0-17 | 30.7% |  |  |  | 2.9 | 0.6 | 19.7% |  |  |  |
| GCS 14 (calc) | Osmond | 2010 | < 17 | 8.5% |  |  |  | 3.0 | 0.9 | 0.8% |  |  |  |
| GCS 14 (calc) | Munivenkatappa | 2013 | <13 | 44.6% | 1.3 | 0.8 | 19.5% |  |  |  |  |  |  |
| GCS 14 (calc) | Melo | 2008 | 0-19 | 4.5% | 10.9 | 0.8 | 2.5% |  |  |  |  |  |  |
| **GCS 13-14 only** |  |  |  |  |  |  |  |  |  |  |  |  |  |
| GCS 13-14 | Osmond | 2010 | <17 | 9.8% |  |  |  | 3.2 | 0.8 | 1.2% |  |  |  |
| *GCS 13-14 at 2 h vs GCS 13-14* | Osmond | 2010 | < 17 | 3.8% |  |  |  | 3.4 | 0.7 | 1.6% |  |  |  |
| *GCS 13-14 at 4 h vs GCS 13-14* | Osmond | 2010 | < 17 | 6.3% |  |  |  | 2.1 | 0.9 | 0.6% |  |  |  |
| *GCS 13-14 at 6 h vs GCS 13-14* | Osmond | 2010 | < 17 | 4.5% |  |  |  | 2.8 | 0.9 | 0.7% |  |  |  |
| GCS 13-14 (calc) | Guzel | 2009 | <17 | 4.8% | 11.6 | 0.7 | 5.0% |  |  |  |  |  |  |
| GCS 13-14 (calc) | Munivenkatappa | 2013 | <13 | 57.9% | 1.2 | 0.8 | 32.3% |  |  |  |  |  |  |
| GCS 13-14 (calc) | Melo | 2008 | 0-19 | 6.3% | 8.9 | 0.7 | 3.3% |  |  |  |  |  |  |
| GCS 13-14 (calc) +sympt. | Castellani | 2009 | <18 | 21.1% | 1.6 | 0.9 | 9.2% | 1.0 | 1.0 | 2.8% |  |  |  |
| GCS 13-14 (calc) | Mandera | 2000 | 0-17 | 42.8% |  |  |  | 2.1 | 0.5 | 25.3% |  |  |  |
| GCS 13-14 (calc) | Atabaki | 2008 | <21 | 14.8% |  |  |  | 2.1 | 0.8 | 1.9% |  |  |  |
| GCS 13-14 (calc) | Klassen | 2000 | <16 | 12.5% |  |  |  | 3.6 | 0.7 | 2.1% |  |  |  |
| GCS 13-14 (calc) | Hahn | 1993 | <16 | 30.6% |  |  |  | 1.4 | 0.8 | 3.5% |  |  |  |

| **4. Severity of head injury** | | | | |  |  |  |  |  |  |  |  |  |  |
| --- | --- | --- | --- | --- | --- | --- | --- | --- | --- | --- | --- | --- | --- | --- |
| **Risk factor** | **GCS compared to** | **Study** | **Year** | **Age** | **Prev risk factor** | **PLR CT** | **NLR CT** | **Prev CT** | **PLR ICI** | **NLR ICI** | **Prev ICI** | **PLR NS** | **NLR NS** | **Prev NS** |
| MILD HEAD INJURY |  |  |  |  |  |  |  |  |  |  |  |  |  |  |
| GCS 14 | 13+15 | Osmond | 2010 | < 17 | 7.3% |  |  |  | 2.8 | 0.9 | 0.8% |  |  |  |
| GCS 15 | 13+14 | Osmond | 2010 | < 17 | 90.3% |  |  |  | 0.8 | 3.2 | 2.9% |  |  |  |
| GCS 15 (+inclusion symptoms) | 13-14 | Castellani | 2009 | < 18 | 78.9% | 0.9 | 1.6 | 23.9% | 1.0 | 1.0 | 10.1% |  |  |  |
| GCS 14 (+inclusion symptoms) | 13+15 | Castellani | 2009 | < 18 | 11.9% | 1.7 | 0.9 | 5.5% | 1.2 | 1.0 | 1.8% |  |  |  |
| GCS 14-15 or normal for age | 3-13 | Da Dalt | 2006 | <16 | 98.7% |  |  |  | 0.5 | 46.1 | 0.3% |  |  |  |
| GCS 14-15 or normal for age (calc.) | 11-13 | Da Dalt | 2006 | <16 | 99.0% |  |  |  | 0.7 | 59.1 | 0.3% |  |  |  |
| GCS 15 | 13-15 | Atabaki | 2008 | <21 | 85.2% |  |  |  | 0.8 | 2.1 | 4.6% |  |  |  |
| GCS 14 | 13+15 | Atabaki | 2008 | <21 | 11.7% |  |  |  | 1.8 | 0.9 | 1.3% |  |  |  |
| GCS 15 | 13-14 | Hahn | 1993 | <16 | 69.4% |  |  |  | 0.8 | 1.4 | 4.9% |  |  |  |
| GCS 14 | 13+15 | Hahn | 1993 | <16 | 15.7% |  |  |  | 1.2 | 1.0 | 1.5% |  |  |  |
| Mild | Mod-severe | Murgio | 2001 | 0-15 | 43.7% | 0.4 | 1.7 | 8.2% |  |  |  |  |  |  |
| GCS 13-15 | Mod-severe | Ratan | 2005 | children | 64.0% |  |  |  | 0.6 | 2.3 | 13.8% |  |  |  |
| MODERATE HEAD INJURY |  |  |  |  |  |  |  |  |  |  |  |  |  |  |
| PCS <13 (any) | 13-15 | Ramundo | 1995 | <2y | 16.2% | 3.6 | 0.7 | 8.1% |  |  |  |  |  |  |
| PCS <13 (any) | 13-15 | Ramundo | 1995 | 2-18 | 13.5% | 7.5 | 0.5 | 8.1% |  |  |  |  |  |  |
| GCS < 13 (any) | 13-15 | Ng | 2002 | < 15 | 9.0% | 11.4 | 0.7 | 6.8% |  |  |  |  |  |  |
| Moderate (calc.) | Mild | Murgio | 2001 | 0-15 | 46.2% | 1.7 | 0.6 | 18.5% |  |  |  |  |  |  |
| Moderate | Mild+Sev | Murgio | 2001 | 0-15 | 37.4% | 1.2 | 0.9 | 15.0% |  |  |  |  |  |  |
| GCS 13 | 14-15 | Garcia | 2009 | 0-18 | 1.1% |  |  |  | 14.1 | 0.9 | 0.2% |  |  |  |
| GCS 13 | 14-15 | Melo | 2008 | 0-19 | 1.9% | 6.6 | 0.9 | 0.8% |  |  |  |  |  |  |
| GCS 13 (+inclusion spt) | 14-15 | Castellani | 2009 | < 18 | 9.2% | 1.4 | 1.0 | 3.7% | 0.8 | 1.0 | 0.9% |  |  |  |
| GCS <14 (any) | 14-15 | Dunning | 2006 | <16 | 1.2% |  |  |  | 75.4 | 0.5 | 0.6% |  |  |  |
| GCS <12 (any) | 12-15 | Bechtel | 2009 | < 18 | 10.5% |  |  |  | 4.1 | 0.8 | 4.6% |  |  |  |
| GCS 13 | 14-15 | Osmond | 2010 | < 17 | 2.5% |  |  |  | 4.4 | 0.9 | 0.4% |  |  |  |
| GCS 13 | 14-15 | Atabaki | 2008 | <21 | 3.1% |  |  |  | 3.5 | 0.9 | 0.6% |  |  |  |
| GCS 13 | 14-15 | Hahn | 1993 | <16 | 14.9% |  |  |  | 1.7 | 0.9 | 2.0% |  |  |  |
| GCS 13 | 14-15 | Mandera | 2000 | 0-17 | 17.5% |  |  |  | 0.9 | 1.5 | 9.0% |  |  |  |
| GCS 9-12 (moderate) calc. | 13-15 | Ratan | 2006 | children | 25.6% |  |  |  | 2.2 | 0.7 | 11.6% |  |  |  |
| GCS 11-13 (calc.) | GCS 14-15/  normal for age | Da Dalt | 2006 | <16 | 0.5% |  |  |  | 7.4 | 0.9 | 0.03% |  |  |  |
| SEVERE HEAD INJURY |  |  |  |  |  |  |  |  |  |  |  |  |  |  |
| GCS ≤ 8 vs GCS 9-15 | 9-15 | Chan | 1990 | 11-15 | 1.4% |  |  |  | 31.2 | 0.8 | 0.7% | 31.2 | 0.8 | 0.72% |
| GCS ≤ 8 (severe) | 9-15 | Ratan | 2007 | children | 14.0% |  |  |  | 3.9 | 0.8 | 9.3% |  |  |  |
| Severe | Mild-mod | Murgio | 2001 | 0-15 | 18.9% | 3.2 | 0.7 | 11.9% |  |  |  |  |  |  |

| **5. Altered mental status** | | | |  |  |  |  |  |  |  |  |  |  |
| --- | --- | --- | --- | --- | --- | --- | --- | --- | --- | --- | --- | --- | --- |
| **Risk factor** | **Study** | **Year** | **Age** | **Prev risk factor** | **PLR CT** | **NLR CT** | **Prev CT** | **PLR ICI** | **NLR ICI** | **Prev ICI** | **PLR NS** | **NLR NS** | **Prev NS** |
| **If AGE < 4y only** |  |  |  |  |  |  |  |  |  |  |  |  |  |
| Altered mental status | Kupperman | 2009 | < 2 | 11.4% |  |  |  | 4.7 | 0.5 | 0.5% |  |  |  |
| Depressed mental status (any) | Greenes | 1999 | < 2 | 7.9% |  |  |  | 0.7 | 1.2 | 1.3% |  |  |  |
| depressed mental status (severe) | Greenes | 1999 | < 2 | 0.5% |  |  |  | 37.8 | 0.9 | 0.3% |  |  |  |
| depressed mental status (moderate/worse) | Greenes | 1999 | < 2 | 3.5% |  |  |  | 7.7 | 0.8 | 1.0% |  |  |  |
| Irritability (history) | Greenes | 1999 | <2 | 15.8% |  |  |  | 2.0 | 0.8 | 1.5% |  |  |  |
| Irritability | Greenes | 1999 | <2 | 12.6% |  |  |  | 2.3 | 0.8 | 1.3% |  |  |  |
| Altered consciousness (GCS 14 vs GCS 15) | Shane | 1997 | < 13 mo | 5.9% |  |  |  | 29.0 | 0.7 | 4.9% |  |  |  |
| Altered level of alertness (any) | Oman | 2006 | 0-3 | 34.0% |  |  |  | 2.2 | 0.5 | 5.50% |  |  |  |
| No spontaneous eye opening (assumpt) vs spontaneous | Oman | 2006 | 0-3 | 90.9% |  |  |  | 7.4 | 0.5 | 4.20% |  |  |  |
| Not able to follow commands (assumpt) vs able | Oman | 2006 | 0-3 | 93.2% |  |  |  | 2.5 | 0.4 | 5.50% |  |  |  |
| Depressed level of consciousness | Gruskin | 1999 | < 2 | 5.0% | 3.3 | 0.9 | 2.2% |  |  |  |  |  |  |
| **If AGE = any** |  |  |  |  |  |  |  |  |  |  |  |  |  |
| Altered mental status | Kupperman | 2009 | < 2 | 11.4% |  |  |  | 4.7 | 0.5 | 0.5% |  |  |  |
| Depressed mental status (any) | Greenes | 1999 | < 2 | 7.9% |  |  |  | 0.7 | 1.2 | 1.3% |  |  |  |
| Depressed mental status (severe) | Greenes | 1999 | < 2 | 0.5% |  |  |  | 37.8 | 0.9 | 0.3% |  |  |  |
| Depressed mental status (moderate/worse) | Greenes | 1999 | < 2 | 3.5% |  |  |  | 7.7 | 0.8 | 1.0% |  |  |  |
| Altered consciousness (GCS 14 vs GCS 15) | Shane | 1997 | <13 mo | 5.9% |  |  |  | 29.0 | 0.7 | 4.9% |  |  |  |
| No spontaneous eye opening (assumpt) vs spontaneous | Oman | 2006 | 0-3 | 90.9% |  |  |  | 7.4 | 0.5 | 4.20% |  |  |  |
| Not able to follow commands (assumpt) vs able | Oman | 2006 | 0-3 | 93.2% |  |  |  | 2.5 | 0.4 | 5.50% |  |  |  |
|  |  |  |  |  |  |  |  |  |  |  |  |  |  |
| Altered mental status | Kupperman | 2009 | 2-17 | 13.6% |  |  |  | 4.8 | 0.4 | 0.6% |  |  |  |
| Altered level of alertness | Fabbri | 2011 | < 11 | 10.7% |  |  |  | 8.8 | 0.1 | 0.7% |  |  |  |
| Mental status change | Atabaki | 2008 | < 21 | 15.7% |  |  |  | 1.9 | 0.8 | 1.8% |  |  |  |
| Abnormal mental status | Palchak | 2003 | < 18 | 42.9% |  |  |  | 2.1 | 0.3 | 6.5% |  |  |  |
| Altered mental status | Quayle | 1997 | <18 | 13.7% |  |  |  | 3.0 | 0.7 | 3.0% | 1.6 | 0.8 | 1.25% |
| Altered level of alertness | Oman | 2006 | 0-18 | 30.2% |  |  |  | 2.4 | 0.5 | 5.5% |  |  |  |
| Not able to follow commands vs able (assumpt) | Oman | 2006 | 0-18 | 12.9% |  |  |  | 6.2 | 0.5 | 4.6% |  |  |  |
| Not oriented vs. oriented (assumpt) | Oman | 2006 | 0-18 | 16.3% |  |  |  | 5.3 | 0.4 | 5.3% |  |  |  |
| No spontaneous eye opening vs spontaneous (assumpt) | Oman | 2006 | 0-18 | 9.8% |  |  |  | 8.7 | 0.5 | 4.3% |  |  |  |
|  |  |  |  |  |  |  |  |  |  |  |  |  |  |
| GCS 14 vs GCS 15 | Kupperman | 2009 | 2-17 | 2.8% |  |  |  | 10.3 | 0.8 | 0.2% |  |  |  |
| GCS 14 vs GCS 15 | Munoz-Sanchez | 2005 | <14 | 6.7% |  |  |  | 3.7 | 0.8 | 1.7% |  |  |  |
| GCS 14* vs GCS 15* (*incl. LOC) | Simon | 2001 | <16 | 12.3% |  |  |  | 0.5 | 1.3 | 2.6% |  |  |  |
| GCS 14 vs GCS 15 (calc) | Mandera | 2000 | 0-17 | 30.7% |  |  |  | 2.9 | 0.6 | 19.7% |  |  |  |
| GCS 14 vs GCS 15 (calc) | Hahn | 1993 | <16 | 18.4% |  |  |  | 1.3 | 0.9 | 1.8% |  |  |  |
| GCS 14 (calc) | Osmond | 2010 | < 17 | 8.5% |  |  |  | 3.0 | 0.9 | 0.8% |  |  |  |
| Drowsiness or amnesia | Fabbri | 2011 | < 11 | 14.3% |  |  |  | 6.5 | 0.1 | 0.7% |  |  |  |
| Persistent drowsiness | Da Dalt | 2006 | <16 | 3.7% |  |  |  | 5.1 | 0.8 | 0.1% |  |  |  |
| Drowsiness | Dunning | 2006 | < 16 | 4.2% |  |  |  | 5.4 | 0.8 | 0.3% |  |  |  |
| Drowsiness | Quayle | 1997 | <18 | 45.2% |  |  |  | 1.2 | 0.8 | 4.5% | 0.8 | 1.0 | 0.3% |
| Sleepiness | Schunk | 1996 | <18 | 38.3% |  |  |  | 1.5 | 0.9 | 2.2% | 0.2 | 1.5 | 0.3% |
| Impaired consciousness | Chan | 1990 | <16 | 2.9% |  |  |  | 43.9 | 0.2 | 1.1% |  |  |  |
| Depressed level of consciousness | Gruskin | 1999 | < 2 | 5.0% | 3.3 | 0.9 | 2.2% |  |  |  |  |  |  |
| Abnormal mental status | Klemetti | 2009 | < 17 | 39.4% | 2.0 | 0.5 | 11.5% |  |  |  |  |  |  |
| Abnormal mental status (NexusII) | Klemetti | 2009 | < 17 | 39.4% | 2.1 | 0.5 | 12.0% |  |  |  |  |  |  |
| GCS 14 vs GCS 15 (calc) | Munivenkatappa | 2013 | <13 | 44.6% | 1.3 | 0.8 | 19.5% |  |  |  |  |  |  |
| GCS 14 vs GCS 15 (calc) | Melo | 2008 | 0-19 | 4.5% | 10.9 | 0.8 | 2.5% |  |  |  |  |  |  |
|  |  |  |  |  |  |  |  |  |  |  |  |  |  |
| Irritability (history) | Greenes | 1999 | <2 | 15.8% |  |  |  | 2.0 | 0.8 | 1.5% |  |  |  |
| Irritability | Greenes | 1999 | <2 | 12.6% |  |  |  | 2.3 | 0.8 | 1.3% |  |  |  |
| Irritability | Schunk | 1996 | <18 | 22.0% |  |  |  | 1.6 | 0.7 | 1.3% | 0.0 | 1.2 | 0.00% |

| **6. Abnormal behaviour (according to guardian)** | | | |  |  |  |  |  |  |  |  |  |  |
| --- | --- | --- | --- | --- | --- | --- | --- | --- | --- | --- | --- | --- | --- |
| **Risk factor** | **Study** | **Year** | **Age** | **Prev risk factor** | **PLR CT** | **NLR CT** | **Prev CT** | **PLR ICI** | **NLR ICI** | **Prev ICI** | **PLR NS** | **NLR NS** | **Prev NS** |
| **If age < 4y** |  |  |  |  |  |  |  |  |  |  |  |  |  |
| Abnormal patient | Kupperman | 2009 | < 2 | 14.0% |  |  |  | 3.4 | 0.6 | 0.4% |  |  |  |
| Abnormal behaviour | Oman | 2006 | 0-3 | 37.5% |  |  |  | 1.5 | 0.7 | 4.5% |  |  |  |
| History of behavioural change | Gruskin | 1999 | <2 | 29.5% | 1.1 | 1.0 | 4.9% |  |  |  |  |  |  |
| **If age = any** |  |  |  |  |  |  |  |  |  |  |  |  |  |
| Abnormal behaviours | Fabbri | 2011 | < 11 | 10.3% |  |  |  | 7.4 | 0.3 | 54.4% |  |  |  |
| Abnormal behaviour (NexusII) | Klemetti | 2009 | <17 | 38.6% |  |  |  | 1.6 | 0.7 | 9.5% |  |  |  |
| Abnormal behaviour | Oman | 2006 | 0-18 | 25.1% |  |  |  | 2.1 | 0.7 | 4.0% |  |  |  |
| Abnormal patient | Kupperman | 2009 | < 2 | 14.0% |  |  |  | 3.4 | 0.6 | 0.4% |  |  |  |
| History of behavioural change | Gruskin | 1999 | <2 | 29.5% | 1.1 | 1.0 | 4.9% |  |  |  |  |  |  |

| **7. Drowsiness/lethargy/irritability/confusion** | | | |  |  |  |  |  |  |  |  |  |  |
| --- | --- | --- | --- | --- | --- | --- | --- | --- | --- | --- | --- | --- | --- |
| **Risk factor** | **Study** | **Year** | **Age** | **Prev risk factor** | **PLR CT** | **NLR CT** | **Prev CT** | **PLR ICI** | **NLR ICI** | **Prev ICI** | **PLR NS** | **NLR NS** | **Prev NS** |
| **If Age < 4y** |  |  |  |  |  |  |  |  |  |  |  |  |  |
| irritability (history) | Greenes | 1999 | <2 | 15.8% |  |  |  | 2.0 | 0.8 | 1.5% |  |  |  |
| Irritability | Greenes | 1999 | <2 | 12.6% |  |  |  | 2.3 | 0.8 | 1.3% |  |  |  |
| Lethargy (history) | Greenes | 1999 | <2 | 4.9% |  |  |  | 7.0 | 0.8 | 1.3% |  |  |  |
| Lethargy* (only if positive CT) | Shane | 1997 | <13 mo | 28.1% |  |  |  | 4.0 | 0.6 | 21.9% |  |  |  |
| Not oriented (assumption) vs oriented | Oman | 2006 | 0-3 | 65.37% |  |  |  | 3.0 | 0.4 | 5.5% |  |  |  |
| **If Age = any** |  |  |  |  |  |  |  |  |  |  |  |  |  |
| Drowsy | Ng | 2002 | <15 | 28.9% | 1.4 | 0.9 | 7.7% |  |  |  |  |  |  |
| Persistent drowsiness | Da Dalt | 2006 | <16 | 3.7% |  |  |  | 5.1 | 0.8 | 0.1% |  |  |  |
| Drowsiness | Dunning | 2006 | < 16 | 4.2% |  |  |  | 5.4 | 0.8 | 0.3% |  |  |  |
| Drowsiness | Quayle | 1997 | <18 | 45.2% |  |  |  | 1.2 | 0.8 | 4.52% | 0.8 | 1.0 | 0.31% |
| Drowsiness or amnesia | Fabbri | 2011 | < 11 | 14.3% |  |  |  | 6.5 | 0.1 | 0.7% |  |  |  |
| Sleepiness | Schunk | 1996 | <18 | 38.3% |  |  |  | 1.5 | 0.9 | 2.2% | 0.2 | 1.5 | 0.32% |
| Lethargy | Atabaki | 2008 | <21 | 28.7% |  |  |  | 1.3 | 0.9 | 2.4% |  |  |  |
| Lethargy* (only if positive CT) | Shane | 1997 | <13 mo | 28.1% |  |  |  | 4.0 | 0.6 | 21.9% |  |  |  |
| Lethargy (history) | Greenes | 1999 | <2 | 4.9% |  |  |  | 7.0 | 0.8 | 1.3% |  |  |  |
| Lethargy | Mitchell | 1994 | <17 | 23.7% | 0.8 | 1.1 | 3.2% |  |  |  |  |  |  |
| irritability (history) | Greenes | 1999 | <2 | 15.8% |  |  |  | 2.0 | 0.8 | 1.5% |  |  |  |
| Irritability | Greenes | 1999 | <2 | 12.6% |  |  |  | 2.3 | 0.8 | 1.3% |  |  |  |
| irritability | Schunk | 1996 | <18 | 22.0% |  |  |  | 1.6 | 0.7 | 1.3% | 0.0 | 1.2 | 0.00% |
| Confusion | Osmond | 2010 | < 17 | 59.3% |  |  |  | 1.1 | 0.8 | 2.4% |  |  |  |
| Witnessed disorientation | Klassen | 2000 | <16 | 20.5% |  |  |  | 1.0 | 1.0 | 1.0% |  |  |  |
| Not oriented (assumpt) vs oriented | Oman | 2006 | 0-18 | 16.33% |  |  |  | 5.3 | 0.4 | 5.3% |  |  |  |
| Confusion | Schunk | 1996 | <18 | 20.1% |  |  |  | 1.4 | 0.9 | 1.0% | 0.9 | 1.0 | 0.64% |
| Confusion | Quayle | 1997 | <18 | 27.8% |  |  |  | 1.2 | 0.9 | 2.8% | 0.0 | 1.1 | 0% |

| **8. Amnesia** | | | |  |  |  |  |  |  |  |  |  |  |
| --- | --- | --- | --- | --- | --- | --- | --- | --- | --- | --- | --- | --- | --- |
| **Risk factor** | **Study** | **Year** | **Age** | **Prev risk factor** | **PLR CT** | **NLR CT** | **Prev CT** | **PLR ICI** | **NLR ICI** | **Prev ICI** | **PLR NS** | **NLR NS** | **Prev NS** |
| **If Age = any** |  |  |  |  |  |  |  |  |  |  |  |  |  |
| Amnesia | Bechtel | 2009 | < 18 | 26.3% |  |  |  | 0.6 | 1.2 | 2.6% |  |  |  |
| Amnesia | Osmond | 2010 | < 17 | 58.5% |  |  |  | 2.4 | 0.4 | 2.4% |  |  |  |
| Amnesia | Atabaki | 2008 | <21 | 31.6% |  |  |  | 0.7 | 1.1 | 1.5% |  |  |  |
| Amnesia | Palchak | 2003 | <18 | 56.4% |  |  |  | 1.3 | 0.6 | 3.7% |  |  |  |
| Short term memory deficits | Haydel | 2003 | 5-17 | 10.3% |  |  |  | 1.4 | 1.0 | 1.1% |  |  |  |
| Amnesia | Klassen | 2000 | <16 | 46.4% |  |  |  | 0.7 | 1.3 | 1.7% |  |  |  |
| Amnesia | Davis | 1994 | 2-17 | 36.3% |  |  |  | 0.6 | 1.3 | 1.8% |  |  | 0.0% |
| Amnesia | Quayle | 1997 | <18 | 6.6% |  |  |  | 1.4 | 1.0 | 0.8% | 0.0 | 1.2 | 0.0% |
| Amnesia | Schunk | 1996 | <18 | 19.8% |  |  |  | 1.3 | 0.9 | 1.0% | 0.0 | 1.2 | 0.0% |
| Amnesia for event | Dietrich | 1993 | 2-21 | 38.3% | 0.8 | 2.1 | 15.0% | 1.2 | 0.7 | 10.7% |  |  |  |
| Amnesia | Hallen | 2010 | < 18 | 58.6% | 1.3 | 0.5 | 3.6% |  |  |  |  |  |  |
| Amnesia | Ramundo | 1995 | 2-18 | 51.1% | 1.1 | 0.9 | 5.1% |  |  |  |  |  |  |
| Amnesia | Mitchell | 1994 | <17 | 4.5% | 5.9 | 0.4 | 0.5% |  |  |  |  |  |  |
| Antero/retrograd amnesia | Ng | 2002 | <15 | 9.6% | 1.9 | 0.9 | 3.2% |  |  |  |  |  |  |
| Postraumatic amnesia | Guzel | 2009 | <17 | 3.6% | 9.3 | 0.8 | 1.5% |  |  |  |  |  |  |
| Posttraumatic amnesia | DaDalt | 2006 | < 16 | 2.1% |  |  |  | 4.5 | 0.9 | 0.1% |  |  |  |
| Amnesia > 5min | Dunning | 2006 | <16 | 1.3% |  |  |  | 22.0 | 0.8 | 0.3% |  |  |  |

| **9. Vomiting** | | | |  |  |  |  |  |  |  |  |  |  |
| --- | --- | --- | --- | --- | --- | --- | --- | --- | --- | --- | --- | --- | --- |
| **Risk factor** | **Study** | **Year** | **Age** | **Prev risk factor** | **PLR CT** | **NLR CT** | **Prev CT** | **PLR ICI** | **NLR ICI** | **Prev ICI** | **PLR NS** | **NLR NS** | **Prev NS** |
| **If age <4y** |  |  |  |  |  |  |  |  |  |  |  |  |  |
| Vomiting | Ramundo | 1995 | <2 | 18.9% | 0.6 | 1.1 | 2.7% |  |  |  |  |  |  |
| Vomiting | Dietrich | 1993 | <2 | 19.7% | 0.8 | 1.0 | 2.8% | 0.0 | 1.3 | 0.0% |  |  |  |
| Persistent vomiting | Oman | 2006 | 0-3 | 13.0% |  |  |  | 1.0 | 1.0 | 1.1% |  |  |  |
| Vomiting (history) calc | Greenes | 1999 | <2 | 4.6% |  |  |  | 1.5 | 1.0 | 0.3% |  |  |  |
| Emesis* (if CT performed) | Shane | 1997 | <13 mo | 4.9% |  |  |  | 11.6 | 0.9 | 2.0% |  |  |  |
| Emesis | Gruskin | 1999 | < 2 | 11.0% | 0.9 | 1.0 | 1.5% |  |  |  |  |  |  |
| **If Age = any** |  |  |  |  |  |  |  |  |  |  |  |  |  |
| vomiting at 6-8h since injury | Xiao | 2013 | 3 -18 | 1.7% | 8.0 | 0.9 | 0.4% |  |  |  |  |  |  |
| Vomiting at 8-10h since injury | Xiao | 2013 | 3 -18 | 1.4% | 7.1 | 0.9 | 0.3% |  |  |  |  |  |  |
| Vomiting at 10-12h since injury | Xiao | 2013 | 3 -18 | 1.0% | 8.9 | 0.9 | 0.3% |  |  |  |  |  |  |
| Vomiting at 6-12h since injury | Xiao | 2013 | 3 -18 | 4.2% | 7.9 | 0.8 | 1.0% |  |  |  |  |  |  |
| Vomiting | Hallen | 2010 | < 18 | 31.5% | 0.0 | 1.6 | 0.0% |  |  |  |  |  |  |
| Vomiting (NexusII) | Klemetti | 1999 | <17 | 48.0% | 1.0 | 1.0 | 8.2% |  |  |  |  |  |  |
| Vomiting | Guzel | 2009 | <17 | 15.2% | 1.8 | 0.9 | 1.9% |  |  |  |  |  |  |
| Vomiting | Ramundo | 1995 | 2-18 | 19.3% | 0.7 | 1.1 | 2.3% |  |  |  |  |  |  |
| Vomiting | Ramundo | 1995 | <2 | 18.9% | 0.6 | 1.1 | 2.7% |  |  |  |  |  |  |
| Vomiting | Mitchell | 1994 | <17 | 34.2% | 0.6 | 1.3 | 6.2% |  |  |  |  |  |  |
| Vomiting | Munivenkatappa | 2013 | <13 | 57.1% | 1.1 | 0.9 | 30.8% |  |  |  |  |  |  |
| Vomiting Y/N | Ng | 2002 | <15 | 68.2% | 1.1 | 0.9 | 8.7 |  |  |  |  |  |  |
| Emesis | Gruskin | 1999 | < 2 | 11.0% | 0.9 | 1.0 | 1.5% |  |  |  |  |  |  |
| Vomiting | Dietrich | 1993 | 2-21 | 38.7% | 0.4 | 6.8 | 8.7% | 0.4 | 2.6 | 4.0% |  |  |  |
| Vomiting | Dietrich | 1993 | <2 | 19.7% | 0.8 | 1.0 | 2.8% | 0.0 | 1.3 | 0.0% |  |  |  |
| Vomiting | Bechtel | 2009 | < 18 | 20.4% |  |  |  | 2.2 | 0.8 | 5.9% |  |  |  |
| Vomiting, any (history) | Kupperman | 2009 | 2-17 | 12.7% |  |  |  | 2.8 | 0.7 | 0.3% |  |  |  |
| Vomiting | Davis | 1994 | 2-17 | 6.5% |  |  |  | 1.1 | 1.0 | 0.6% |  |  | 0.0% |
| Vomiting | Atabaki | 2008 | <21 | 33.2% |  |  |  | 0.8 | 1.1 | 1.7% |  |  |  |
| History of vomiting | Palchak | 2003 | <18 | 20.1% |  |  |  | 1.9 | 0.8 | 2.3% |  |  |  |
| Emesis (any) | Haydel | 2003 | 5-17 | 19.4% |  |  |  | 2.5 | 0.7 | 3.4% |  |  |  |
| Vomiting (history) calc. | Greenes | 1999 | <2 | 4.6% |  |  |  | 1.5 | 1.0 | 0.3% |  |  |  |
| Vomiting | Da Dalt | 2007 | <15 | 13.6% |  |  |  | 1.8 | 0.9 | 0.2% |  |  |  |
| Vomiting | Quayle | 1997 | <18 | 26.8% |  |  |  | 1.3 | 0.9 | 2.9% | 0.7 | 1.0 | 0.3% |
| Vomiting | Schunk | 1996 | <18 | 34.8% |  |  |  | 1.4 | 0.8 | 1.9% | 0.7 | 1.1 | 0.96% |
| Vomiting | Chan | 1990 | 11-15 | 26.6% |  |  |  | 1.5 | 0.8 | 1.2% |  |  |  |
| Vomiting | Chan | 1990 | <16 | 39.3% |  |  |  | 0.9 | 1.0 | 0.5% |  |  |  |
| Emesis * (if CT performed) | Shane | 1997 | <13 mo | 4.9% |  |  |  | 11.6 | 0.9 | 2.0% |  |  |  |
| Vomiting (at least 1 time) | Da Dalt | 2006 | <16 | 12.9% |  |  |  | 1.9 | 0.9 | 0.1% |  |  |  |
| vomit once | Da Dalt | 2006 | <16 | 7.2% |  |  |  | 1.3 | 1.0 | 0.1% |  |  |  |
| Vomiting, once | Garcia | 2009 | 0-18 | 8.8% |  |  |  | 1.5 | 0.9 | 0.2% |  |  |  |
| **Vomiting > once** |  |  |  |  |  |  |  |  |  |  |  |  |  |
| Vomiting ≥ 3 times | Dunning | 2006 | <16 | 3.8% |  |  |  | 5.6 | 0.8 | 0.2% |  |  |  |
| Vomit >1 time (calc) | Kupperman | 2009 | 2-17 | 5.1% |  |  |  | 2.5 | 0.9 | 0.2% |  |  |  |
| Vomit ≥ 2 times | Osmond | 2010 | < 17 | 40.9% |  |  |  | 1.2 | 0.9 | 2.0% |  |  |  |
| Vomiting a/o persistent vomiting(fused) | Fabbri | 2011 | < 11 | 11.6% |  |  |  | 2.4 | 0.8 | 0.2% |  |  |  |
| Persistent vomiting | Oman | 2006 | 0-18 | 12.1% |  |  |  | 2.2 | 0.9 | 2.0% |  |  |  |
| Persistent vomiting | Oman | 2006 | 0-3 | 13.0% |  |  |  | 1.0 | 1.0 | 1.1% |  |  |  |
| Repeated vomiting | Da Dalt | 2006 | <16 | 5.7% |  |  |  | 2.5 | 0.9 | 0.1% |  |  |  |
| Vomiting repetitive | Garcia | 2009 | 0-18 | 13.1% |  |  |  | 1.5 | 0.9 | 0.3% |  |  |  |

| **10. Headache** | | | |  |  |  |  |  |  |  |  |  |  |
| --- | --- | --- | --- | --- | --- | --- | --- | --- | --- | --- | --- | --- | --- |
| **Risk factor** | **Study** | **Year** | **Age** | **Prev risk factor** | **PLR CT** | **NLR CT** | **Prev CT** | **PLR ICI** | **NLR ICI** | **Prev ICI** | **PLR NS** | **NLR NS** | **Prev NS** |
| **If age = any** |  |  |  |  |  |  |  |  |  |  |  |  |  |
| Moderate-severe progressive HA |  |  |  |  |  |  |  |  |  |  |  |  |  |
| Moderate-severe HA vs no-light HA (calc) | Kupperman | 2009 | 2-17 | 21.3% |  |  |  | 2.0 | 0.7 | 0.3% |  |  |  |
| Moderate Headache | Kupperman | 2009 | 2-17 | 21.2% |  |  |  | 2.0 | 0.7 | 0.3% |  |  |  |
| Severe Headache | Kupperman | 2009 | 2-17 | 3.0% |  |  |  | 4.3 | 0.9 | 0.1% |  |  |  |
| *Prolonged/worsening HA* | Da Dalt | 2006 | <16 | 2.0% |  |  |  | 13.1 | 0.8 | 0.1% |  |  |  |
| Severe or progressive headache | Oman | 2006 | 0-18 | 15.4% |  |  |  | 1.3 | 0.9 | 1.7% |  |  |  |
| Severe or progressive headache | Oman | 2006 | 0-3 | 4.6% |  |  |  | 0.0 | 1.1 | 0.0% |  |  |  |
| Progressive headache | Quayle | 1997 | < 18 | 62.9% |  |  |  | 1.0 | 1.0 | 5.2% | 0.9 | 1.0 | 0.3% |
| Headache | Bechtel | 2009 | < 18 | 44.1% |  |  |  | 0.5 | 1.4 | 3.9% |  |  |  |
| Headache | Kupperman | 2009 | 2-17 | 41.9% |  |  |  | 1.6 | 0.4 | 0.5% |  |  |  |
| Headache | Osmond | 2010 | < 17 | 18.6% |  |  |  | 1.8 | 0.8 | 1.2% |  |  |  |
| Headache | Fabbri | 2011 | < 11 | 2.9% |  |  |  | 19.2 | 0.5 | 0.4% |  |  |  |
| Headache | Atabaki | 2008 | <21 | 37.5% |  |  |  | 0.7 | 1.2 | 1.7% |  |  |  |
| Headache (any vs none) (calc.) | Da Dalt | 2006 | <16 | 9.0% |  |  |  | 2.8 | 0.8 | 0.1% |  |  |  |
| brief HA | Da Dalt | 2006 | <16 | 7.0% |  |  |  | 0.0 | 1.1 | 0.0% |  |  |  |
| Headache | Palchak | 2003 | < 18 | 56.6% |  |  |  | 1.2 | 0.8 | 3.0% |  |  |  |
| Headache | Haydel | 2003 | 5-17 | 34.9% |  |  |  | 1.5 | 0.8 | 4.0% |  |  |  |
| Headache | Quayle | 1997 | <18 | 53.5% |  |  |  | 0.9 | 1.1 | 4.2% | 0.0 | 1.1 | 0% |
| Headache | Schunk | 1996 | <18 | 30.0% |  |  |  | 1.4 | 0.9 | 1.6% | 0.3 | 1.3 | 0.3% |
| Headache | Davis | 1994 | 2 - 17 | 23.2% |  |  |  | 1.3 | 0.9 | 2.4% |  |  | 0.0% |
| Headache | Chan | 1990 | 11-15 | 24.2% |  |  |  | 0.6 | 1.1 | 0.5% |  |  |  |
| Headache | Dietrich | 1993 | 2-21 | 37.2% | 0.3 | 7.5 | 7.1% | 0.3 | 2.7 | 3.2% |  |  |  |
| Headache | Hallen | 2010 | < 18 | 59.5% | 1.2 | 0.6 | 2.7% |  |  |  |  |  |  |
| Headache | Guzel | 2009 | <17 | 7.2% | 6.3 | 0.7 | 2.4% |  |  |  |  |  |  |
| Headache | Ramundo | 1995 | 2-18 | 67.0% | 1.0 | 1.0 | 5.4% |  |  |  |  |  |  |
| Headache | Mitchell | 1994 | <17 | 20.9% | 0.9 | 1.0 | 3.2% |  |  |  |  |  |  |
| Headache | Ng | 2002 | <15 | 15.1% | 0.8 | 1.0 | 2.6% |  |  |  |  |  |  |
| Commencement of headache <2h  since injury | Xiao | 2013 | 3 -18 | 77.5% | 1.2 | 0.5 | 2.4% |  |  |  |  |  |  |
| Commencement of headache <6h  since injury calc | Xiao | 2013 | 3 -18 | 78.0% | 1.2 | 0.3 | 3.6% |  |  |  |  |  |  |
| Commencement of headache <10h  since injury calc | Xiao | 2013 | 3 -18 | 78.7% | 1.3 | 0.1 | 3.8% |  |  |  |  |  |  |
| Commencement of HA 4-6h  since injury | Xiao | 2013 | 3 -18 | 0.5% | 12.5 | 1.0 | 0.2% |  |  |  |  |  |  |
| Commencement of HA 6-8h  since injury | Xiao | 2013 | 3 -18 | 0.4% | 8.3 | 1.0 | 0.1% |  |  |  |  |  |  |
| Commencement of HA 8-10h  since injury | Xiao | 2013 | 3 -18 | 0.3% | 16.7 | 1.0 | 0.1% |  |  |  |  |  |  |
| Worsening HA 2-4h since injury | Xiao | 2013 | 3 -18 | 0.3% | 99.9 | 0.9 | 0.2% |  |  |  |  |  |  |
| Worsening HA 4-6h since injury | Xiao | 2013 | 3 -18 | 0.4% | 33.3 | 0.9 | 0.2% |  |  |  |  |  |  |
| Worsening headache "<6h" (calc) | Xiao | 2013 | 3 -18 | 0.6% | 50.0 | 0.9 | 0.4% |  |  |  |  |  |  |
| Worsening HA 6-8h since injury | Xiao | 2013 | 3 -18 | 1.8% | 13.6 | 0.8 | 0.6% |  |  |  |  |  |  |
| Worsening HA 8-10h since injury | Xiao | 2013 | 3 -18 | 1.7% | 10.9 | 0.9 | 0.5% |  |  |  |  |  |  |
| Worsening HA 10-12h since injury | Xiao | 2013 | 3 -18 | 0.7% | 18.7 | 0.9 | 0.3% |  |  |  |  |  |  |
| Worsening headache "<12h" | Xiao | 2013 | 3 -18 | 4.9% | 15.8 | 0.5 | 1.9% |  |  |  |  |  |  |
| Persistent headache >/= 2h | Xiao | 2013 | 3 -18 | 77.5% | 1.2 | 0.5 | 3.4% |  |  |  |  |  |  |
| Persistent headache >/= 8h | Xiao | 2013 | 3 -18 | 56.6% | 1.2 | 0.7 | 2.6% |  |  |  |  |  |  |
| Persistent headache >/= 12h | Xiao | 2013 | 3 -18 | 25.1% | 2.0 | 0.7 | 1.9% |  |  |  |  |  |  |
| Persistent headache >/= 24h | Xiao | 2013 | 3 -18 | 12.9% | 2.7 | 0.8 | 1.3% |  |  |  |  |  |  |
|  |  |  |  |  |  |  |  |  |  |  |  |  |  |

| **11. Loss of consciousness (LOC)** | | | |  |  |  |  |  |  |  |  |  |  |
| --- | --- | --- | --- | --- | --- | --- | --- | --- | --- | --- | --- | --- | --- |
| **Risk factor** | **Study** | **Year** | **Age** | **Prev risk factor** | **PLR CT** | **NLR CT** | **Prev CT** | **PLR ICI** | **NLR ICI** | **Prev ICI** | **PLR NS** | **NLR NS** | **Prev NS** |
| **If age < 4y** |  |  |  |  |  |  |  |  |  |  |  |  |  |
| LOC | Gruskin | 1999 | <2 | 5.7% | 0.9 | 1.0 | 0.8% |  |  |  |  |  |  |
| LOC | Ramundo | 1995 | <2 | 32.3% | 0.5 | 1.3 | 3.2% |  |  |  |  |  |  |
| LOC | Dietrich | 1993 | <2 | 14.1% | 1.2 | 1.0 | 2.8% | 2.5 | 0.8 | 1.4% |  |  |  |
| History of LOC (susp/known) | Kupperman | 2009 | < 2 | 5.3% |  |  |  | 4.9 | 0.8 | 0.2% |  |  |  |
| LOC | Oman | 2006 | 0-3 | 34.6% |  |  |  | 2.0 | 0.5 | 5.2% |  |  |  |
| LOC | Shane | 1997 | <13 mo | 3.9% |  |  |  | 11.6 | 0.9 | 2.0% |  |  |  |
| LOC | Greenes | 1999 | <2 | 2.8% |  |  |  | 1.2 | 1.0 | 0.2% |  |  |  |
| **If age = any** |  |  |  |  |  |  |  |  |  |  |  |  |  |
| LOC | Hallen | 2010 | < 18 | 33.3% | 1.5 | 0.8 | 2.7% |  |  |  |  |  |  |
| LOC (observed) | Klemetti | 2009 | <17 | 35.1% | 1.7 | 0.7 | 9.1% |  |  |  |  |  |  |
| LOC | Guzel | 2009 | <17 | 2.8% | 6.7 | 0.9 | 1.0% |  |  |  |  |  |  |
| LOC | Ramundo | 1995 | 2-18 | 45.8% | 1.7 | 0.5 | 11.3% |  |  |  |  |  |  |
| LOC | Ramundo | 1995 | <2 | 32.3% | 0.5 | 1.3 | 3.2% |  |  |  |  |  |  |
| LOC | Mitchell | 1994 | <17 | 38.4% | 0.6 | 1.3 | 5.5% |  |  |  |  |  |  |
| LOC | Munivenkatappa | 2013 | <13 | 38.3% | 1.0 | 1.0 | 19.5% |  |  |  |  |  |  |
| LOC y/n | Ng | 2002 | <15 | 28.0% | 2.1 | 0.7 | 10.0% |  |  |  |  |  |  |
| LOC | Gruskin | 1999 | <2 | 5.7% | 0.9 | 1.0 | 0.8% |  |  |  |  |  |  |
| LOC | Dietrich | 1993 | <2 | 14.1% | 1.2 | 1.0 | 2.8% | 2.5 | 0.8 | 1.4% |  |  |  |
| LOC | Dietrich | 1993 | 2-21 | 31.2% | 0.6 | 2.3 | 11.5% | 0.4 | 0.9 | 7.5% |  |  |  |
| LOC | Bechtel | 2009 | < 18 | 54.0% |  |  |  | 0.9 | 1.1 | 7.9% |  |  |  |
| History of LOC (susp/known) | Kupperman | 2009 | 2-17 | 18.9% |  |  |  | 3.1 | 0.5 | 0.5% |  |  |  |
| History of LOC (susp/known) | Kupperman | 2009 | < 2 | 5.3% |  |  |  | 4.9 | 0.8 | 0.2% |  |  |  |
| LOC (witnessed) | Osmond | 2010 | < 17 | 32.8% |  |  |  | 0.9 | 1.1 | 1.2% |  |  |  |
| LOC | Fabbri | 2011 | < 11 | 9.7% |  |  |  | 6.6 | 0.4 | 0.5% |  |  |  |
| LOC | Atabaki | 2008 | <21 | 32.6% |  |  |  | 0.7 | 1.2 | 1.5% |  |  |  |
| LOC (verified Y/N) calc | Da Dalt | 2006 | <16 | 2.8% |  |  |  | 14.5 | 0.6 | 0.2% |  |  |  |
| temporary (LOC) | Da Dalt | 2006 | <16 | 2.1% |  |  |  | 0.0 | 1.0 | 0.0% |  |  |  |
| LOC | Oman | 2006 | 0-18 | 56.7% |  |  |  | 1.4 | 0.6 | 6.2% |  |  |  |
| LOC | Oman | 2006 | 0-3 | 34.6% |  |  |  | 2.0 | 0.5 | 5.2% |  |  |  |
| LOC | Boran | 2006 | <17 | 5.5% |  |  |  | 16.1 | 0.6 | 1.2% |  |  |  |
| LOC | Palchak | 2003 | <18 | 59.4% |  |  |  | 1.4 | 0.5 | 5.7% |  |  |  |
| LOC a/o amnesia | Palchak | 2004 | <18 | 41.8% |  |  |  | 2.2 | 0.3 | 3.7% |  |  |  |
| LOC “only” | Palchak | 2004 | < 18 | 36.2% |  |  |  | 2.3 | 0.3 | 3.0% |  |  |  |
| LOC “only” in isolation | Palchak | 2004 | < 18 |  |  |  |  | 0 | No data |  |  |  |  |
| LOC (+ GCS 15) | Simon | 2001 | <16 | 45.8% |  |  |  | 0.6 | 1.3 | 4.8% |  |  |  |
| LOC (+ GCS 14) | Simon | 2001 | <16 | 68.8% |  |  |  | 1.1 | 0.8 | 16.7% |  |  |  |
| *LOC (+ GCS 14-15)* ***calc*** | Simon | 2001 | <16 | 43.4% |  |  |  | 0.8 | 1.2 | 5.6% |  |  |  |
| LOC | Klassen | 2000 | <16 | 68.6% |  |  |  | 1.2 | 0.6 | 4.1% |  |  |  |
| LOC | Schunk | 1996 | <18 | 25.9% |  |  |  | 0.7 | 1.1 | 0.6% | 0.0 | 1.3 | 0.0% |
| LOC | Chan | 1990 | 11-15 | 6.2% |  |  |  | 1.9 | 0.6 | 1.9% |  |  |  |
| LOC | Greenes | 1999 | <2 | 2.8% |  |  |  | 1.2 | 1.0 | 0.2% |  |  |  |
| LOC | Shane | 1997 | <13 mo | 3.9% |  |  |  | 11.6 | 0.9 | 2.0% |  |  |  |
| LOC documented (?) | Wang | 2000 | <15 | 33.1% |  |  |  | 0.9 | 1.0 | 5.7% |  |  | 0.6% |
| LOC < 1 min | Davis | 1994 | 2 -17 | 18.5% |  |  |  | 0.4 | 1.2 | 0.6% |  |  | 0.0% |
| LOC | Garcia | 2009 | 0-19 | 6.5% |  |  |  | 3.1 | 0.9 | 0.3% |  |  |  |
| **LOC ≥ 1min** |  |  |  |  |  |  |  |  |  |  |  |  |  |
| LOC >5 min | Ramundo | 1995 | <2 | 12.9% | 1.4 | 0.9 | 3.2% |  |  |  |  |  |  |
| LOC >5 min | Ramundo | 1995 | 2-18 | 10.6% | 6.0 | 0.7 | 5.7% |  |  |  |  |  |  |
| LOC ≥ 5 min (vs < 5 min or absent) calc. | Ng | 2002 | <15 | 2.6% | 6.3 | 0.9 | 1.6% |  |  |  |  |  |  |
| LOC > 1min | Kupperman | 2009 | < 2 | 1.6% |  |  |  | 4.0 | 0.9 | 0.0% |  |  |  |
| LOC > 1min | Kupperman | 2009 | 2-17 | 4.0% |  |  |  | 5.0 | 0.8 | 0.1% |  |  |  |
| LOC ≥ 1min | Osmond | 2010 | < 17 | 15.7% |  |  |  | 1.1 | 1.0 | 0.7% |  |  |  |
| Prolonged LOC (witnessed > 5 min) | Oman | 2006 | 0-3 | 10.4% |  |  |  | 4.8 | 0.7 | 3.1% |  |  |  |
| Prolonged LOC (witnessed >5 min) | Oman | 2006 | 0-18 | 9.9% |  |  |  | 6.0 | 0.6 | 3.5% |  |  |  |
| Prolonged LOC | Da Dalt | 2006 | <16 | 0.7% |  |  |  | 75.6 | 0.6 | 0.2% |  |  |  |
| LOC > 5 min | Dunning | 2006 | <16 | 0.9% |  |  |  | 64.4 | 0.7 | 0.4% |  |  |  |
| LOC > 5 min | Quayle | 1997 | <18 | 6.2% |  |  |  | 3.1 | 0.9 | 1.4% | 0.0 | 1.3 | 0.0% |
| LOC 1-15 min | Davis | 1994 | 2 to 17 | 26.8% |  |  |  | 1.7 | 0.8 | 3.6% |  |  | 0.0% |
| LOC > 15 min | Davis | 1994 | 2 to 17 | 0.6% |  |  |  | 0.0 | 1.0 | 0.0% |  |  | 0.0% |
| LOC moderate vs no LOC (calc) | Ratan | 2001 | children | 56.5% |  |  |  | 1.0 | 1.1 | 12.2% |  |  |  |
| LOC mod-severe vs no LOC (calc) | Ratan | 2001 | children | 74.3% |  |  |  | 1.2 | 0.6 | 27.0% |  |  |  |
| *LOC ≤ 15 min (moderate) vs any* | Ratan | 2001 | children | 33.5% |  |  |  | 0.6 | 1.3 | 7.3% |  |  |  |
| *LOC >15 min (severe) vs none-moderate* | Ratan | 2001 | children | 40.8% |  |  |  | 1.9 | 0.6 | 19.8% |  |  |  |

| **12. Focal neurology** | | | |  |  |  |  |  |  |  |  |  |  |
| --- | --- | --- | --- | --- | --- | --- | --- | --- | --- | --- | --- | --- | --- |
| **Risk factor** | **Study** | **Year** | **Age** | **Prev risk factor** | **PLR CT** | **NLR CT** | **Prev CT** | **PLR ICI** | **NLR ICI** | **Prev ICI** | **PLR NS** | **NLR NS** | **Prev NS** |
| **If age = any** |  |  |  |  |  |  |  |  |  |  |  |  |  |
| Focal neurology | Hallen | 2010 | < 18 | 7.2% | 5.7 | 0.7 | 1.8% |  |  |  |  |  |  |
| Abnormal neurological findings | Guzel | 2009 | <17 | 0.2% | na | 1.0 | 0.2% |  |  |  |  |  |  |
| Neurologic abnormalities | Lloyd | 1997 | < 17 | 52.6% | 2.0 | 0.2 | 13.5% |  |  |  |  |  |  |
| Focal neurodeficits | Mitchell | 1994 | <17 | 2.7% | 6.6 | 0.9 | 0.3% |  |  |  |  |  |  |
| Neurological deficit (NexusII) | Klemetti | 2011 | <17 | 7.2% | 9.3 | 0.7 | 4.7% |  |  |  |  |  |  |
| Cranial nerve symptom | Klemetti | 2009 | <17 | 3.1% | 67.8 | 0.8 | 2.9% |  |  |  |  |  |  |
| Focal neurologic deficit | Ramundo | 1995 | <2 | 8.1% | 7.3 | 0.8 | 5.4% |  |  |  |  |  |  |
| Focal neurologic deficit | Ramundo | 1995 | 2-18 | 2.3% | 24.0 | 0.9 | 1.9% |  |  |  |  |  |  |
| Anisocoria | Ramundo | 1995 | <2 | 8.1% | 7.3 | 0.8 | 5.4% |  |  |  |  |  |  |
| Anisocoria | Ramundo | 1995 | 2-18 | 5.0% | 16.0 | 0.8 | 3.8% |  |  |  |  |  |  |
| Ataxia | Ramundo | 1995 | <2 | 3.7% | 0.0 | 1.1 | 0.0% |  |  |  |  |  |  |
| Ataxia | Ng | 2002 | <15 | 4.5% | 2.1 | 1.0 | 1.6% |  |  |  |  |  |  |
| Focal neurological deficit | Ng | 2002 | <15 | 2.9% | 30.3 | 0.9 | 2.6% |  |  |  |  |  |  |
| Abnormal neurologic examination | Gruskin | 1999 | <2 | 0.7% | na | 1.0 | 0.7% |  |  |  |  |  |  |
| Focal neurodeficits | Dietrich | 1993 | <2 | 4.2% | 2.5 | 0.9 | 1.4% | 11.3 | 0.7 | 1.4% |  |  |  |
| focal neurodeficits | Dietrich | 1993 | 2-21 | 8.7% | 0.3 | 1.9 | 4.4% | 0.7 | 1.2 | 4.0% |  |  |  |
| Abnormal neurologic exam (fused) | Fabbri | 2011 | < 11 | 0.8% |  |  |  | 105.5 | 0.6 | 0.3% |  |  |  |
| Positive focal neurology | Dunning | 2006 | <16 | 0.7% |  |  |  | 45.0 | 0.8 | 0.2% |  |  |  |
| Neurological exam (abnorm vs normal) | Da Dalt | 2006 | <16 | 0.3% |  |  |  | 118.0 | 0.8 | 0.1% |  |  |  |
| Focal neurologic deficit | Palchak | 2003 | <18 | 3.1% |  |  |  | 6.7 | 0.9 | 1.1% |  |  |  |
| Focal neurologic deficit | Quayle | 1997 | <18 | 2.5% |  |  |  | 7.3 | 0.9 | 1.0% | 0.0 | 1.8 | 0.00% |
| Abnormal neurologic ex | Davis | 1994 | 2-17 | 16.7% |  |  |  | 2.4 | 0.8 | 3.0% | 1.6 | 0.9 | 1.19% |
| Neurological signs | Chan | 1990 | 11-15 | 4.1% |  |  |  | 1.9 | 1.0 | 0.2% |  |  |  |
| Motor deficit | Oman | 2006 | 0-18 | 4.5% |  |  |  | 7.0 | 0.8 | 1.7% |  |  |  |
| Composite score, neurologic deficit | Oman | 2006 | 0-18 | 36.1% |  |  |  | 2.5 | 0.3 | 6.7% |  |  |  |
| Cranial nerve abnormality | Oman | 2006 | 0-18 | 13.9% |  |  |  | 0.1 | 1.2 | 0.2% |  |  |  |
| Gait abnormality | Oman | 2006 | 0-18 | 6.3% |  |  |  | 4.2 | 0.8 | 1.7% |  |  |  |
| Abnormal cerebellar | Oman | 2006 | 0-18 | 2.8% |  |  |  | 6.0 | 0.9 | 1.0% |  |  |  |
| Inability to read or write | Oman | 2006 | 0-18 | 14.5% |  |  |  | 4.8 | 0.5 | 4.4% |  |  |  |
| Sensory deficit | Atabaki | 2008 | <21 | 0.6% |  |  |  | 7.2 | 1.0 | 0.2% |  |  |  |
| Focal neurologic deficit | Shane | 1997 | <13 mo | 2.0% |  |  |  | na | 0.9 | 2.0% |  |  |  |
| **If age < 4 y** |  |  |  |  |  |  |  |  |  |  |  |  |  |
| Focal neurologic deficit | Shane | 1997 | <13 mo | 2.0% |  |  |  | na | 0.9 | 2.0% |  |  |  |
| Motor deficit | Oman | 2006 | 0-3 | 3.9% |  |  |  | 13.0 | 0.8 | 2.1% |  |  |  |
| Composite score, neurologic deficit | Oman | 2006 | 0-3 | 56.2% |  |  |  | 1.5 | 0.4 | 6.6% |  |  |  |
| Cranial nerve abnormality | Oman | 2006 | 0-3 | 2.5% |  |  |  | 20.0 | 0.8 | 1.6% |  |  |  |
| Gait abnormality | Oman | 2006 | 0-3 | 7.3% |  |  |  | 3.7 | 0.8 | 1.8% |  |  |  |
| Abnormal cerebellar | Oman | 2006 | 0-3 | 2.7% |  |  |  | 22.0 | 0.8 | 1.8% |  |  |  |
| Inability to read or write | Oman | 2006 | 0-3 | 38.3% |  |  |  | 1.8 | 0.6 | 5.2% |  |  |  |
| Focal neurodeficits | Dietrich | 1993 | <2 | 4.2% | 2.5 | 0.9 | 1.4% | 11.3 | 0.7 | 1.4% |  |  |  |
| Focal neurologic deficit | Ramundo | 1995 | <2 | 8.1% | 7.3 | 0.8 | 5.4% |  |  |  |  |  |  |
| Ataxia | Ramundo | 1995 | <2 | 3.7% | 0.0 | 1.1 | 0.0% |  |  |  |  |  |  |
| Anisocoria | Ramundo | 1995 | <2 | 8.1% | 7.3 | 0.8 | 5.4% |  |  |  |  |  |  |
| Abnormal neurologic examination | Gruskin | 1999 | <2 | 0.7% | na | 1.0 | 0.7% |  |  | 0.7% |  |  |  |

| **13. Seizures** | | | |  |  |  |  |  |  |  |  |  |  |
| --- | --- | --- | --- | --- | --- | --- | --- | --- | --- | --- | --- | --- | --- |
| **Risk factor** | **Study** | **Year** | **Age** | **Prev risk factor** | **PLR CT** | **NLR CT** | **Prev CT** | **PLR ICI** | **NLR ICI** | **Prev ICI** | **PLR NS** | **NLR NS** | **Prev NS** |
| **If age = any** |  |  |  |  |  |  |  |  |  |  |  |  |  |
| Seizures | Guzel | 2009 | <17 | 1.1% | 12.7 | 0.9 | 0.5% |  |  |  |  |  |  |
| Seizure | Ramundo | 1995 | 2-18 | 5.5% | 2.8 | 0.9 | 2.0% |  |  |  |  |  |  |
| Seizure | Ramundo | 1995 | <2 | 18.9% | 0.6 | 1.1 | 2.7% |  |  |  |  |  |  |
| Seizure | Mitchell | 1994 | <17 | 7.5% | 5.9 | 0.4 | 0.5% |  |  |  |  |  |  |
| Seizures | Munivenkatappa | 2013 | <13 | 8.3% | 1.1 | 1.0 | 4.5% |  |  |  |  |  |  |
| Seizure | Ng | 2002 | <15 | 3.2% | 2.5 | 1.0 | 1.3% |  |  |  |  |  |  |
| Seizure | Gruskin | 1999 | <2 | 3.4% | 0.7 | 1.0 | 0.4% |  |  |  |  |  |  |
| Seizure | Dietrich | 1993 | <2 | 0.0% | na | 1.0 | 0.0% | na | 1.0 | 0.0% |  |  |  |
| Seizure | Dietrich | 1993 | 2-21 | 6.3% | 0.2 | 2.0 | 2.2% | 0.3 | 2.7 | 1.6% |  |  |  |
| Seizure | Atabaki | 2008 | <21 | 5.7% |  |  |  | 2.0 | 0.9 | 0.7% |  |  |  |
| Seizures (any) | Osmond | 2010 | < 17 | 3.9% |  |  |  | 1.1 | 1.0 | 0.2% |  |  |  |
| Seizure | Palchak | 2003 | <18 | 5.0% |  |  |  | 1.0 | 2.5 | 0.8% |  |  |  |
| Seizure | Greenes | 1999 | <2 | 1.8% |  |  |  | 4.2 | 0.9 | 0.3% |  |  |  |
| Seizure | Quayle | 1997 | <18 | 5.0% |  |  |  | 2.7 | 0.9 | 1.0% | 0.0 | 1.3 | 0.00% |
| Seizure | Schunk | 1996 | <18 | 8.0% |  |  |  | 1.5 | 1.0 | 0.3% | 1.5 | 1.0 | 0.32% |
| Seizure | Davis | 1994 | 2-17 | 4.2% |  |  |  | 1.8 | 1.0 | 0.6% |  |  |  |
| Seizures (only if CT) | Shane | 1997 | <13 mo | 1.0% |  |  |  | na | 0.9 | 1.0% |  |  |  |
| Epilepsy | Chan | 1990 | <16 | 0.8% |  |  |  | 18.3 | 0.9 | 0.2% |  |  |  |
| Impact seizure (Y vs N) | Da Dalt | 2006 | <16 | 0.6% |  |  |  | 24.6 | 0.9 | 0.1% |  |  |  |
| Impact seizure | Fabbri | 2011 | < 11 | 0.5% |  |  |  | 111.0 | 0.7 | 0.3% |  |  |  |
| Seizure after head injury | Dunning | 2006 | <16 | 0.4% |  |  |  | 33.0 | 0.9 | 0.1% |  |  |  |
| Seizure after trauma | Oman | 2006 | 0-18 | 6.0% |  |  |  | 1.0 | 1.0 | 0.5% |  |  |  |
| Seizures posttrauma | Boran | 2006 | <17 | 1.4% |  |  |  | 51.9 | 0.9 | 1.2% |  |  |  |
| Postraumatic seizures | Haydel | 2003 | 5-17 | 3.4% |  |  |  | 0.0 | 1.0 | 0.0% |  |  |  |
| Posttraumatic epilepsy | Chan | 1990 | 11-15 | 2.4% |  |  |  | 0.0 | 1.0 | 0.0% |  |  |  |
|  |  |  |  |  |  |  |  |  |  |  |  |  |  |
| **If age < 4y** |  |  |  |  |  |  |  |  |  |  |  |  |  |
| Seizure | Greenes | 1999 | <2 | 1.8% |  |  |  | 4.2 | 0.9 | 0.3% |  |  |  |
| Seizure after trauma | Oman | 2006 | 0-3 | 8.9% |  |  |  | 0.9 | 1.0 | 0.6% |  |  |  |
| Seizures (only if CT) | Shane | 1997 | <13 mo | 1.0% |  |  |  | na | 0.9 | 1.0% |  |  |  |
| Seizure | Dietrich | 1993 | <2 | 0.0% | na | 1.0 | 0.0% | na | 1.0 | 0.0% |  |  |  |
| Seizure | Ramundo | 1995 | <2y | 18.9% | 0.6 | 1.1 | 2.7% |  |  |  |  |  |  |
| Seizure | Gruskin | 1999 | <2 | 3.4% | 0.7 | 1.0 | 0.4% |  |  |  |  |  |  |

| **14. Skull fractures** | | | |  |  |  |  |  |  |  |  |  |  |
| --- | --- | --- | --- | --- | --- | --- | --- | --- | --- | --- | --- | --- | --- |
| **Risk factor** | **Study** | **Year** | **Age** | **Prev risk factor** | **PLR CT** | **NLR CT** | **Prev CT** | **PLR ICI** | **NLR ICI** | **Prev ICI** | **PLR NS** | **NLR NS** | **Prev NS** |
| **Age <4** |  |  |  |  |  |  |  |  |  |  |  |  |  |
| palpable skull fracture | Kupperman | 2009 | < 2 | 3.4% |  |  |  | 11.0 | 0.7 | 0.3% |  |  |  |
| Evidence of significant skull fracture | Oman | 2006 | 0-3 | 2.7% |  |  |  | 5.5 | 0.9 | 0.9% |  |  |  |
| temporal fracture (on CT) | Shane | 1997 | <13 mo | 15.6% |  |  |  | na | 0.7 | 15.6% |  |  |  |
| frontal fracture (on CT) | Shane | 1997 | <13 mo | 12.5% |  |  |  | 3.4 | 0.9 | 9.4% |  |  |  |
| Depressed SF | Ramundo | 1995 | <2 | 5.4% | na | 0.8 | 5.4% |  |  |  |  |  |  |
| **Age any** |  |  |  |  |  |  |  |  |  |  |  |  |  |
| Clinical sign of skull fracture | Klemetti | 2009 | <17 | 5.4% | na | 0.7 | 5.4% |  |  |  |  |  |  |
| Fracture (any) | Osmond | 2010 | < 17 | 3.7% |  |  |  | 9.7 | 0.8 | 1.1% |  |  |  |
| Skull fracture | Simon | 2001 | <16 | 22.8% |  |  |  | 2.4 | 0.7 | 9.3% |  |  |  |
| Skull fracture | Quayle | 1997 | <18 | 26.7% |  |  |  | 4.0 | 0.2 | 7.2% | 1.7 | 0.7 | 1.56% |
| Skull fracture vs no fracture | Hahn | 1993 | <16 | 48.9% |  |  |  | 1.6 | 0.5 | 6.2% |  |  |  |
| Skull# | Chan | 1990 | 11-15 | 6.2% |  |  |  | 31.2 | 0.0 | 3.1% | 22.3 | 0.2 | 2.4% |
| Clinical signs of skull fracture | Palchak | 2003 | <18 | 5.3% |  |  |  | 6.3 | 0.8 | 1.8% |  |  |  |
| Evidence of significant skull fracture | Oman | 2006 | 0-18 | 5.1% |  |  |  | 9.3 | 0.7 | 2.3% |  |  |  |
| Evidence of significant skull fracture | Oman | 2006 | 0-3 | 2.7% |  |  |  | 5.5 | 0.9 | 0.9% |  |  |  |
| palpable skull fracture | Kupperman | 2009 | < 2 | 3.4% |  |  |  | 11.0 | 0.7 | 0.3% |  |  |  |
| Skull defect - susp. Fracture? | Atabaki | 2008 | <21 | 2.9% |  |  |  | 3.0 | 0.9 | 0.5% |  |  |  |
| Signs of skull fracture  (risky area or susp depressed #) | Da Dalt | 2006 | <16 | 5.5% |  |  |  | 9.6 | 0.5 | 0.3% |  |  |  |
| Depressed SF | Ramundo | 1995 | <2y | 5.4% | na | 0.8 | 5.4% |  |  |  |  |  |  |
| depressed skull# | Ramundo | 1995 | 2-18 | 7.3% | 8.2 | 0.8 | 4.6% |  |  |  |  |  |  |
| penetrating trauma | Ramundo | 1995 | 2-18 | 1.9% | 7.2 | 0.9 | 1.2% |  |  |  |  |  |  |
| Penetrating or depressed skull injury,  susp or tense fontanelle | Dunning | 2006 | <16 | 0.5% |  |  |  | 63.5 | 0.8 | 0.2% |  |  |  |
| Evidence of skull or skullbase  fracture (fused) | Fabbri | 2011 | < 11 | 0.8% |  |  |  | 164.8 | 0.4 | 0.4% |  |  |  |
| Evidence of skull or skullbase  fracture (DaDalt) | Fabbri | 2011 | < 11 |  |  |  |  | 185.3 | 0.4 |  |  |  |  |
| Evidence of skull fracture  (NEXUSII) | Fabbri | 2011 | < 11 |  |  |  |  | 185.3 | 0.4 |  |  |  |  |
| suspected skull base fracture | Dunning | 2006 | <16 | 2.4% |  |  |  | 15.1 | 0.7 | 0.4% |  |  |  |
| Skull base fracture | Osmond | 2010 | < 17 | 3.1% |  |  |  | 10.0 | 0.8 | 0.9% |  |  |  |
| Basilar skull fracture | Kupperman | 2009 | 2-17 | 0.7% |  |  |  | 21.7 | 0.9 | 0.1% |  |  |  |
| Signs of skull base fracture  (yes vs no) | Da Dalt | 2006 | <16 | 0.3% |  |  |  | 43.0 | 0.9 | 0.1% |  |  |  |
| Basal skull fracture | Atabaki | 2008 | <21 | 2.5% |  |  |  | 2.0 | 1.0 | 0.3% |  |  |  |
| signs of basilar fracture | Quayle | 1997 | <18 | 1.7% |  |  |  | 8.2 | 0.9 | 0.8% | 0.0 | 1.9 | 0% |
| racoon eyes | Ramundo | 1995 | 2-18 | 1.5% | 1.6 | 1.0 | 0.4% |  |  |  |  |  |  |
| battle sign | Ramundo | 1995 | 2-18 | 0.8% | na | 1.0 | 0.8% |  |  |  |  |  |  |
| Periorbital eccymoses | Guzel | 2009 | <17 | 3.2% | 11.8 | 0.8 | 1.5% |  |  |  |  |  |  |
| Rhinorrhea | Guzel | 2009 | <17 | 0.4% | 12.7 | 1.0 | 0.2% |  |  |  |  |  |  |
| rhinorrea | Ramundo | 1995 | 2-18 | 0.8% | 0.0 | 1.0 | 0.0% |  |  |  |  |  |  |
| CSF leak | Mitchell | 1994 | <17 | 3.5% | 35.5 | 0.4 | 1.8% |  |  |  |  |  |  |
| Otorrhea | Guzel | 2009 | <17 | 1.3% | 38.0 | 0.9 | 1.0% |  |  |  |  |  |  |
| Otorrhea | Ramundo | 1995 | 2-18 | 2.7% | 6.4 | 0.9 | 1.5% |  |  |  |  |  |  |
| Skull fracture on XR | Lloyd | 1997 | <17 | 68.6% | 0.9 | 1.1 | 9.6% |  |  |  |  |  |  |
| SXR abnormal vs SXR normal* | Melo | 2008 | 0-19 | 23.7% | 7.9 | 0.4 | 18.5% |  |  |  |  |  |  |
| temporal fracture on CT | Shane | 1997 | <13 mo | 15.6% |  |  |  | na | 0.7 | 15.6% |  |  |  |
| frontal fracture on CT | Shane | 1997 | <13 mo | 12.5% |  |  |  | 3.4 | 0.9 | 9.4% |  |  |  |
| XR positive for fracture | Boran | 2006 | <17 | 10.2% |  |  |  | 6.2 | 0.6 | 3.8% |  |  |  |
| Skull fracture on CT** GCS 13-15 | Mandera | 2000 | 0-17 | 62.0% |  |  |  | 0.8* | 1.5* | 18.7% |  |  |  |
| Skull fracture on CT(?) GCS 13-15 | Levi | 1991 | <15 | 74.7% |  |  |  | 0.9 | 1.4 | 11.5% |  |  |  |
| Skull fracture on CT(?) GCS any | Levi | 1991 | <15 | 72.0% |  |  |  | 0.9 | 1.2 | 23.7% |  |  |  |

*XR+CT performed on all

**ICI=edh,sdh only

| **15. Scalp haematoma & location** | | | |  |  |  |  |  |  |  |  |  |  |
| --- | --- | --- | --- | --- | --- | --- | --- | --- | --- | --- | --- | --- | --- |
| **Risk factor** | **Study** | **Year** | **Age** | **Prev risk factor** | **PLR CT** | **NLR CT** | **Prev CT** | **PLR ICI** | **NLR ICI** | **Prev ICI** | **PLR NS** | **NLR NS** | **Prev NS** |
| **If Age < 4 y** |  |  |  |  |  |  |  |  |  |  |  |  |  |
| Scalp hematoma | Kupperman | 2009 | < 2 | 44.2% |  |  |  | 1.5 | 0.6 | 0.6% |  |  |  |
| Scalp hematoma | Oman | 2006 | 0-3 | 42.9% |  |  |  | 1.6 | 0.6 | 5.3% |  |  |  |
| Small scalp hematoma | Greenes | 2001 | 0-23mo | 21.1% |  |  |  | 0.7 | 1.1 | 2.6% |  |  |  |
| Scalp hematoma and ≤ 2y old | Palchak | 2003 | <18 | 6.1% |  |  |  | 2.7 | 0.9 | 6.5% |  |  |  |
| Scalp abnormality | Gruskin | 1999 | <2 | 61.2% | 1.8 | 0.1 | 17.3% |  |  |  |  |  |  |
| **Size of hematoma** |  |  |  |  |  |  |  |  |  |  |  |  |  |
| Scalp hematoma significant | Greenes | 1999 | <2 | 43.1% |  |  |  | 1.9 | 0.4 | 3.8% |  |  |  |
| Large scalp hematoma | Greenes | 2001 | 0-23mo | 22.4% |  |  |  | 3.4 | 0.5 | 9.2% |  |  |  |
| Moderate scalp hematoma | Greenes | 2001 | 0-23mo | 30.3% |  |  |  | 0.7 | 1.1 | 3.9% |  |  |  |
| **Location** |  |  |  |  |  |  |  |  |  |  |  |  |  |
| Parietal scalp hematoma | Greenes | 2001 | 0-23mo | 43.3% |  |  |  | 2.4 | 0.2 | 14.5% |  |  |  |
| Temporal scalp hematoma | Greenes | 2001 | 0-23mo | 5.3% |  |  |  | 1.6 | 1.0 | 1.3% |  |  |  |
| Temporal haematoma | Kupperman | 2009 | < 2 | 10.0% |  |  |  | 5.0 | 0.6 | 0.4% |  |  |  |
| Occipital haematoma | Kupperman | 2009 | < 2 | 6.0% |  |  |  | 1.7 | 1.0 | 0.1% |  |  |  |
| Occipital scalp hematoma | Greenes | 2001 | 0-23mo | 10.5% |  |  |  | 0.0 | 1.1 | 0.0% |  |  |  |
| Frontal scalp hematoma | Greenes | 2001 | 0-23mo | 19.7% |  |  |  | 0.0 | 1.3 | 0.0% |  |  |  |
| Frontal haematoma | Kupperman | 2009 | < 2 | 28.0% |  |  |  | 0.3 | 1.3 | 0.1% |  |  |  |
| **If Age = any** |  |  |  |  |  |  |  |  |  |  |  |  |  |
| **Large/significant hematoma** |  |  |  |  |  |  |  |  |  |  |  |  |  |
| Large boggy haematoma | Osmond | 2010 | < 17 | 6.6% |  |  |  | 7.0 | 0.7 | 1.5% |  |  |  |
| scalp hematoma significant | Greenes | 1999 | <2 | 43.1% |  |  |  | 1.9 | 0.4 | 3.8% |  |  |  |
| Large scalp hematoma | Greenes | 2001 | 0-23mo | 22.4% |  |  |  | 3.4 | 0.5 | 9.2% |  |  |  |
| Moderate scalp hematoma | Greenes | 2001 | 0-23mo | 30.3% |  |  |  | 0.7 | 1.1 | 3.9% |  |  |  |
| Scalp hematoma | Kupperman | 2009 | < 2 | 44.2% |  |  |  | 1.5 | 0.6 | 0.6% |  |  |  |
| Small scalp hematoma | Greenes | 2001 | 0-23mo | 21.1% |  |  |  | 0.7 | 1.1 | 2.6% |  |  |  |
| Scalp hematoma and ≤ 2y old | Palchak | 2003 | <18 | 6.1% |  |  |  | 2.7 | 0.9 | 6.5% |  |  |  |
| Cephalhematoma | Garcia | 2009 | 0-18 | 9.6% |  |  |  | 5.9 | 0.5 | 0.7% |  |  |  |
| Scalp hematoma | Bechtel | 2009 | < 18 | 57.2% |  |  |  | 1.8 | 0.2 | 14.5% |  |  |  |
| Scalp haematoma | Osmond | 2010 | < 17 | 32.4% |  |  |  | 2.3 | 0.4 | 2.9% |  |  |  |
| Scalp hematoma | Fabbri | 2011 | < 11 | 29.7% |  |  |  | 1.5 | 0.8 | 0.3% |  |  |  |
| Scalp hematoma | Atabaki | 2008 | <21 | 27.6% |  |  |  | 1.6 | 0.8 | 2.8% |  |  |  |
| Scalp hematoma | Oman | 2006 | 0-18 | 38.8% |  |  |  | 1.6 | 0.7 | 488.7% |  |  |  |
| Hematoma | Quayle | 1997 | <18 | 37.7% |  |  |  | 1.4 | 0.8 | 4.3% | 0.0 | 1.2 | 0% |
| Scalp hematoma | Davis | 1994 | 2-17 | 16.7% |  |  |  | 1.3 | 0.9 | 1.8% |  |  |  |
| Scalp hematoma | Guzel | 2009 | < 17 | 13.3% | 5.7 | 0.5 | 4.1% |  |  |  |  |  |  |
| Cranial soft tissue trauma | Klemetti | 2009 | <17 | 34.5% | 1.7 | 0.7 | 8.9% |  |  |  |  |  |  |
| Scalp trauma (NexusII) | Klemetti | 2009 | <17 | 64.7% | 1.3 | 0.6 | 13.4% |  |  |  |  |  |  |
| Scalp abnormality | Gruskin | 1999 | <2 | 61.2% | 1.8 | 0.1 | 17.3% |  |  |  |  |  |  |

| **16. Scalp laceration** | | | |  |  |  |  |  |  |  |  |  |  |
| --- | --- | --- | --- | --- | --- | --- | --- | --- | --- | --- | --- | --- | --- |
| **Risk factor** | **Study** | **Year** | **Age** | **Prev risk factor** | **PLR CT** | **NLR CT** | **Prev CT** | **PLR ICI** | **NLR ICI** | **Prev ICI** | **PLR NS** | **NLR NS** | **Prev NS** |
| Head lacerations | Guzel | 2009 | <17 | 8.4% | 4.8 | 0.7 | 2.3% |  |  |  |  |  |  |
| Head abrasions | Guzel | 2009 | <17 | 12.5% | 3.6 | 0.7 | 2.7% |  |  |  |  |  |  |
| Scalp wound | Mitchell | 1994 | <17 | 16.0% | 5.9 | 0.4 | 6.0% |  |  |  |  |  |  |
| Scalp laceration | Atabaki | 2008 | <21 | 9.8% |  |  |  | 0.3 | 1.1 | 0.2% |  |  |  |
| Scalp laceration | Davis | 1994 | 2-17 | 17.9% |  |  |  | 2.2 | 0.8 | 3.0% |  |  | 0.0% |
| abrasion | Quayle | 1997 | <18 | 32.9% |  |  |  | 1.5 | 0.8 | 4.0% |  |  | 0.0% |
| laceration | Quayle | 1997 | <18 | 7.3% |  |  |  | 1.4 | 1.0 | 4.3% |  |  | 0.0% |
| Presence of bruise/swelling or laceration > 5cm in children < 1 year | Dunning | 2006 | <16 | 0.2% |  |  |  | 10.4 | 1.0 | 0.0% |  |  |  |
| cervicocephalic softtissue | Ramundo | 1995 | 2-18 | 68.6% |  |  |  | 1.3 | 0.5 | 14.2% |  |  |  |
| cervicocephalic softtissue | Ramundo | 1995 | <2 | 64.9% |  |  |  | 1.5 | 0.3 | 18.9% |  |  |  |
| Craniofacial soft-tissue injuries | Guzel | 2009 | <17 | 24.0% | 3.8 | 0.3 | 5.6% |  |  |  |  |  |  |
| Craniofacial soft tissue injury | Simon | 2001 | <16 | 36.1% |  |  |  | 3.1 | 0.2 | 12.4% |  |  |  |
| Facial laceration | Davis | 1994 | 2-17 | 47.0% |  |  |  | 0.6 | 1.4 | 2.4% |  |  | 0.0% |

| **17. Deterioration** | | | |  |  |  |  |  |  |  |  |  |  |
| --- | --- | --- | --- | --- | --- | --- | --- | --- | --- | --- | --- | --- | --- |
| **Risk factor** | **Study** | **Year** | **Age** | **Prev risk factor** | **PLR CT** | **NLR CT** | **Prev CT** | **PLR ICI** | **NLR ICI** | **Prev ICI** | **PLR NS** | **NLR NS** | **Prev NS** |
| Deterioration by GCS 2 points | Da Dalt | 2006 | <16 | 0.5% |  |  |  | 34.3 | 0.9 | 0.1% |  |  |  |
| Deterioration (vs no deterioration) | Osmond | 2010 | < 17 | 8.9% |  |  |  | 4.4 | 0.7 | 1.4% |  |  |  |
| Deterioration | Garcia | 2009 | 0-18 | 3.1% |  |  |  | 7.0 | 0.8 | 0.3% |  |  |  |

| **18. Signs of increased ICP** |  |  |  |  |  |  |  |  |  |  |  |  |  |
| --- | --- | --- | --- | --- | --- | --- | --- | --- | --- | --- | --- | --- | --- |
| **Risk factor** | **Study** | **Year** | **Age** | **Prev risk factor** | **PLR CT** | **NLR CT** | **Prev CT** | **PLR ICI** | **NLR ICI** | **Prev ICI** | **PLR NS** | **NLR NS** | **Prev NS** |
| Bulging fontanel | Greenes | 1999 | <2 | 1.3% |  |  |  | 19.3 | 0.9 | 0.7% |  |  |  |
| Vital signs of increased ICP | Greenes | 1999 | <2 | 0.7% |  |  |  | 19.3 | 0.9 | 0.3% |  |  |  |
| Abnormal fundus | Ramundo | 1995 | <2 | 5.3% | na | 0.7 | 5.3% |  |  |  |  |  |  |
| Abnormal fundus | Ramundo | 1995 | 2-18 | 1.5% | 3.5 | 1.0 | 0.5% |  |  |  |  |  |  |

| **19. Helmet use** | | | |  |  |  |  |  |  |  |  |  |  |
| --- | --- | --- | --- | --- | --- | --- | --- | --- | --- | --- | --- | --- | --- |
| **Risk factor** | **Study** | **Year** | **Age** | **Prev risk factor** | **PLR CT** | **NLR CT** | **Prev CT** | **PLR ICI** | **NLR ICI** | **Prev ICI** | **PLR NS** | **NLR NS** | **Prev NS** |
| Helmet use | Osmond | 2010 | < 17 | 30.9% |  |  |  | 1.5 | 0.6 | 2.9% |  |  |  |

| **20. Intoxication** |  |  |  |  |  |  |  |  |  |  |  |  |  |
| --- | --- | --- | --- | --- | --- | --- | --- | --- | --- | --- | --- | --- | --- |
| **Risk factor** | **Study** | **Year** | **Age** | **Prev risk factor** | **PLR CT** | **NLR CT** | **Prev CT** | **PLR ICI** | **NLR ICI** | **Prev ICI** | **PLR NS** | **NLR NS** | **Prev NS** |
| Drug or alcohol intox | Atabaki | 2008 | <21 | 0.9% |  |  |  | 1.8 | 1.0 | 0.1% |  |  |  |
| Evidence of intoxication | Oman | 2006 | 0-18 | 4.9% |  |  |  | 0.8 | 1.0 | 0.3% |  |  |  |
| Drug or alcohol intoxication | Haydel | 2003 | 5-17 | 6.3% |  |  |  | 1.2 | 1.0 | 0.6% |  |  |  |

| **21. Extracranial injury** | | | |  |  |  |  |  |  |  |  |  |  |
| --- | --- | --- | --- | --- | --- | --- | --- | --- | --- | --- | --- | --- | --- |
| **Risk factor** | **Study** | **Year** | **Age** | **Prev risk factor** | **PLR CT** | **NLR CT** | **Prev CT** | **PLR ICI** | **NLR ICI** | **Prev ICI** | **PLR NS** | **NLR NS** | **Prev NS** |
| Associated injury | Chan | 1990 | 11-15 | 5.3% |  |  |  | 3.1 | 0.9 | 0.5% |  |  |  |
| Distant injury | Simon | 2001 | <16 | 18.7% |  |  |  | 0.8 | 1.0 | 2.3% |  |  |  |
| Other fractures (noncranial) | Davis | 1994 | 2-17 | 25.6% |  |  |  | 0.4 | 2.1 | 2.4% |  |  |  |
| Extracranial injury calc  (mod-severe vs nominal) | Wang | 2001 | <15 | 65.6% |  |  |  | 0.9 | 1.2 | 11.5% |  |  | 2.55% |

| **22. Vertigo** | | | |  |  |  |  |  |  |  |  |  |  |
| --- | --- | --- | --- | --- | --- | --- | --- | --- | --- | --- | --- | --- | --- |
| **Risk factor** | **Study** | **Year** | **Age** | **Prev risk factor** | **PLR CT** | **NLR CT** | **Prev CT** | **PLR ICI** | **NLR ICI** | **Prev ICI** | **PLR NS** | **NLR NS** | **Prev NS** |
| Vertigo | Hallen | 2010 | < 18 | 13.5% | 0.0 | 1.2 | 0.0% |  |  |  |  |  |  |
| Vertigo | Klemetti | 2009 | <17 | 34.0% | 1.8 | 0.7 | 9.3% |  |  |  |  |  |  |
| Dizziness | Atabaki | 2008 | <21 | 9.9% |  |  |  | 0.8 | 1.0 | 0.5% |  |  |  |
| dizziness | Quayle | 1997 | <18 | 57.8% | 1.0 | 1.0 | 4.7% | 0.0 | 1.1 | 0.0% |  |  |  |
| Dizziness | Davis | 1994 | 2-17 | 1.8% |  |  |  | 0.0 | 1.0 | 0.0% |  |  | 0.0% |

| **23. Other symptoms / Mixed symptoms** | | | |  |  |  |  |  |  |  |  |  |  |
| --- | --- | --- | --- | --- | --- | --- | --- | --- | --- | --- | --- | --- | --- |
| **Risk factor** | **Study** | **Year** | **Age** | **Prev risk factor** | **PLR CT** | **NLR CT** | **Prev CT** | **PLR ICI** | **NLR ICI** | **Prev ICI** | **PLR NS** | **NLR NS** | **Prev NS** |
| Pallor | Osmond | 2009 | < 16 | 20.8% |  |  |  | 1.9 | 0.8 | 1.6% |  |  |  |
| Nausea | Davis | 1994 | 2 to 17 | 8.9% |  |  |  | 1.7 | 0.9 | 1.2% |  |  |  |
| Nausea | Hallen | 2010 | < 18 | 45.9% | 0.5 | 1.5 | 0.9% |  |  |  |  |  |  |
| Blurred vision | Guzel | 2009 | <17 | 1.6% | 50.7 | 0.8 | 1.3% |  |  |  |  |  |  |
| hematotympanon | Ramundo | 1995 | 2-18y | 5.4% | 12.0 | 0.8 | 3.8% |  |  |  |  |  |  |
| Ear-nose bleeding | Munivenkatappa | 2013 | <13 | 18.0% | 1.9 | 0.9 | 12.0% |  |  |  |  |  |  |
| LOC/seizures vs no LOC,no seizure  (in GCS15 patients) | Boran | 2006 | <17 | 6.9% |  |  |  | 21.0 | 0.5 | 4.5% | 17.4 | 0.7 | 2.14% |

| **24. Falls vs any other mechanism** | | | |  |  |  |  |  |  |  |  |  |  |
| --- | --- | --- | --- | --- | --- | --- | --- | --- | --- | --- | --- | --- | --- |
| **Risk factor** | **Study** | **Year** | **Age** | **Prev risk factor** | **PLR CT** | **NLR CT** | **Prev CT** | **PLR ICI** | **NLR ICI** | **Prev ICI** | **PLR NS** | **NLR NS** | **Prev NS** |
| **If age < 4y** |  |  |  |  |  |  |  |  |  |  |  |  |  |
| Fall | Ramundo | 1995 | <2y | 75.7% | 0.8 | 1.8 | 13.51% |  |  |  |  |  |  |
| Fall ≥1.5m vs fall <1.5m | Greenes | 1999 | <2 | 38.9% |  |  |  | 1.4 | 0.8 | 3.5% |  |  |  |
| Fall down stairs | Greenes | 1999 | <2 | 12.0% |  |  |  | 1.4 | 0.9 | 0.8% |  |  |  |
| Fall > 1.5m (5ft) calc. | Gruskin | 1999 | < 2y | 26.0% | 2.1 | 0.7 | 7.5% |  |  |  |  |  |  |
| Fall > 3m | Gruskin | 1999 | < 2 | 15.4% | 2.1 | 0.8 | 4.4% |  |  |  |  |  |  |
| **If age = any** |  |  |  |  |  |  |  |  |  |  |  |  |  |
| Falling | Klemetti | 2009 | <17 | 53.4% | 0.8 | 1.3 | 7.22% |  |  |  |  |  |  |
| Falls | Guzel | 2009 | <17 | 14.3% | 3.3 | 0.7 | 2.95% |  |  |  |  |  |  |
| Fall | Ramundo | 1995 | <2y | 75.7% | 0.8 | 1.8 | 13.51% |  |  |  |  |  |  |
| Fall | Ramundo | 1995 | 2-18 | 43.0% | 0.6 | 1.4 | 4.82% |  |  |  |  |  |  |
| Fall | Munivenkatappa | 2013 | <13 | 56.4% | 0.9 | 1.1 | 27.8% |  |  |  |  |  |  |
| Fall | Atabaki | 2008 | <21 | 44.4% |  |  |  | 1.4 | 0.7 | 4.0% |  |  |  |
| Fall | Boran | 2006 | <17 | 39.9% |  |  |  | 1.0 | 1.0 | 3.6% |  |  |  |
| Fall | Simon | 2001 | <16 | 31.0% |  |  |  | 1.1 | 1.0 | 4.9% |  |  |  |
| Fall | Davis | 1994 | 2-17 | 25.0% |  |  |  | 1.5 | 0.8 | 3.0% |  |  |  |
| Fall < 1.5m | Bechtel | 2009 | < 18 | 14.5% |  |  |  | 1.2 | 1.0 | 2.6% |  |  |  |
| Fall < 1.5m | Klassen | 2000 | <16 | 25.4% |  |  |  | 0.3 | 1.2 | 0.4% |  |  |  |
| Fall ≥1.5m vs fall <1.5m | Greenes | 1999 | <2 | 38.9% |  |  |  | 1.4 | 0.8 | 3.5% |  |  |  |
| Fall 1-3 m | Osmond | 2010 | < 17 | 16.5% |  |  |  | 2.2 | 0.8 | 1.4% |  |  |  |
| Fall > 1.5m (5ft) calc. | Gruskin | 1999 | < 2y | 26.0% | 2.1 | 0.7 | 7.5% |  |  |  |  |  |  |
| Fall > 1.5m/5 stairs | Klassen | 2000 | <16 | 21.7% |  |  |  | 1.2 | 0.9 | 1.4% |  |  |  |
| Fall > 1.5m | Bechtel | 2009 | < 18 | 17.1% |  |  |  | 1.0 | 1.0 | 2.6% |  |  |  |
| Fall from moving objects | Bechtel | 2009 | < 18 | 11.2% |  |  |  | 1.1 | 1.0 | 2.0% |  |  |  |
| Fall from moving objects | Osmond | 2010 | < 17 | 0.4% |  |  |  | 3.3 | 1.0 | 0.1% |  |  |  |
| Fall from bike | Osmond | 2010 | < 17 | 7.6% |  |  |  | 1.0 | 1.0 | 0.3% |  |  |  |
| Bicycle (fall/injury) | Atabaki | 2008 | <21 | 6.2% |  |  |  | 1.3 | 1.0 | 0.5% |  |  |  |
| Fall from stairs | Bechtel | 2009 | < 18 | 4.0% |  |  |  | 2.7 | 0.9 | 1.3% |  |  |  |
| Fall down stairs | Greenes | 1999 | <2 | 12.0% |  |  |  | 1.4 | 0.9 | 0.8% |  |  |  |
| Fall >3 m | Osmond | 2010 | < 17 | 1.3% |  |  |  | 6.0 | 0.9 | 0.3% |  |  |  |
| Fall >3m | Dunning | 2006 | <16 | 0.6% |  |  |  | 20.2 | 0.9 | 0.1% |  |  |  |
| Fall > 3m | Gruskin | 1999 | < 2 | 15.4% | 2.1 | 0.8 | 4.4% |  |  |  |  |  |  |

| **25. Traffic accidents** | | | |  |  |  |  |  |  |  |  |  |  |
| --- | --- | --- | --- | --- | --- | --- | --- | --- | --- | --- | --- | --- | --- |
| **Risk factor** | **Study** | **Year** | **Age** | **Prev risk factor** | **PLR CT** | **NLR CT** | **Prev CT** | **PLR ICI** | **NLR ICI** | **Prev ICI** | **PLR NS** | **NLR NS** | **Prev NS** |
| **If age = any** |  |  |  |  |  |  |  |  |  |  |  |  |  |
| Road trauma | Klemetti | 2009 | <17 | 23.9% | 1.9 | 0.8 | 6.8% |  |  |  |  |  |  |
| Road accident | Munivenkatappa | 2013 | <13 | 40.6% | 1.2 | 0.9 | 22.6% |  |  |  |  |  |  |
| Motor vehicle accident | Ramundo | 1995 | <2 | 10.8% | 3.6 | 0.8 | 5.4% |  |  |  |  |  |  |
| Motor vehicle accident | Ramundo | 1995 | 2-18 | 28.4% | 2.2 | 0.6 | 8.8% |  |  |  |  |  |  |
| MVA (passenger) | Guzel | 2009 | <17 | 1.6% | 6.3 | 0.9 | 0.6% |  |  |  |  |  |  |
| High-way speed | Osmond | 2010 | < 17 | 0.4% |  |  |  | 6.4 | 1.0 | 0.1% |  |  |  |
| Motor vehicle crash | Osmond | 2010 | < 17 | 3.0% |  |  |  | 2.7 | 1.0 | 0.3% |  |  |  |
| Motor vehicle accident | Bechtel | 2009 | < 18 | 12.5% |  |  |  | 0.6 | 1.1 | 1.3% |  |  |  |
| Dangerous mechanism | Osmond | 2010 | < 17 | 24.7% |  |  |  | 2.4 | 0.4 | 2.3% |  |  |  |
| Motor vehicle crash | Atabaki | 2008 | <21 | 20.4% |  |  |  | 0.5 | 1.1 | 0.7% |  |  |  |
| High-speed RTA | Dunning | 2006 | <16 | 0.9% |  |  |  | 59.5 | 0.7 | 0.4% |  |  |  |
| MVA involvement | Boran | 2006 | <17 | 15.9% |  |  |  | 0.8 | 1.0 | 1.2% |  |  |  |
| Motor vehicle collision | Simon | 2001 | <16 | 20.1% |  |  |  | 0.5 | 1.1 | 1.6% |  |  |  |
| Automobile (occupant) | Davis | 1994 | 2-17 | 35.7% |  |  |  | 1.0 | 1.0 | 3.0% |  |  |  |
| Car-bike collision | Osmond | 2010 | < 17 | 1.0% |  |  |  | 4.1 | 1.0 | 0.2% |  |  |  |
| Motor vehicle-cycle | Klassen | 2000 | <16 | 4.1% |  |  |  | 8.4 | 0.8 | 1.3% |  |  |  |
| MVA-pedestrian accident | Guzel | 2009 | <17 | 5.2% | 6.3 | 0.8 | 1.75% |  |  |  |  |  |  |
| Pedestrian-Car collision | Bechtel | 2009 | < 18 | 23.0% |  |  |  | 1.1 | 1.0 | 3.9% |  |  |  |
| Pedestrian - car collision | Osmond | 2010 | < 17 | 3.4% |  |  |  | 2.1 | 1.0 | 0.3% |  |  |  |
| Pedestrian struck by MV | Atabaki | 2008 | <21 | 8.5% |  |  |  | 0.5 | 1.0 | 0.3% |  |  |  |
| Hit by motor vehicle | Boran | 2006 | <17 | 21.9% |  |  |  | 2.2 | 0.7 | 3.8% |  |  |  |
| pedestrian vs motor vehicle | Simon | 2001 | <16 | 19.1% |  |  |  | 0.6 | 1.1 | 1.6% |  |  |  |
| Motor vehicle vs pedestrian | Davis | 1994 | 2-17 | 13.1% |  |  |  | 0.0 | 1.2 | 0.0% |  |  |  |
| Bicycle accident | Guzel | 2009 | <17 | 3.1% | 2.1 | 1.0 | 0.44% |  |  |  |  |  |  |
| Bicycle injury | Boran | 2006 | <17 | 9.3% |  |  |  | 0.3 | 1.1 | 0.2% |  |  |  |
| Bike accident | Simon | 2001 | <16 | 11.0% |  |  |  | 2.5 | 0.9 | 3.3% |  |  |  |
| Bicycle | Klassen | 2000 | <16 | 11.1% |  |  |  | 1.1 | 1.0 | 0.6% |  |  |  |
| Bicycle crash | Davis | 1994 | 2-17 | 8.9% |  |  |  | 0.0 | 1.1 | 0.0% |  |  |  |
| Pedestrian accident | Guzel | 2009 | <17 | 5.7% | 1.7 | 1.0 | 0.67% |  |  |  |  |  |  |
| Pedestrian | Klassen | 2000 | <16 | 3.4% |  |  |  | 5.3 | 0.9 | 0.8% |  |  |  |
| other vehicle | Simon | 2001 | <16 | 9.1% |  |  |  | 0.5 | 1.1 | 0.7% |  |  |  |
| Sports collision | Bechtel | 2009 | < 18 | 7.9% |  |  |  | 0.0 | 1.1 | 0.0% |  |  |  |

| **26. Assault** | | |  |  |  |  |  |  |  |  |  |  |  |
| --- | --- | --- | --- | --- | --- | --- | --- | --- | --- | --- | --- | --- | --- |
| **Risk factor** | **Study** | **Year** | **Age** | **Prev risk factor** | **PLR CT** | **NLR CT** | **Prev CT** | **PLR ICI** | **NLR ICI** | **Prev ICI** | **PLR NS** | **NLR NS** | **Prev NS** |
| Violence | Klemetti | 2009 | <17 | 2.1% | 0.5 | 1.0 | 0.2% |  |  |  |  |  |  |
| assault | Ramundo | 1995 | 2-18 | 6.5% | 0.3 | 1.1 | 0.4% |  |  |  |  |  |  |
| Assault | Munivenkatappa | 2013 | <13 | 3.0% | 0.9 | 1.0 | 1.5% |  |  |  |  |  |  |
| abuse | Ramundo | 1995 | <2y | 13.5% | 5.4 | 0.7 | 8.1% | 0.6 | 1.0 | 0.1% |  |  |  |
| Child abuse | Atabaki | 2008 | <21 | 0.5% |  |  |  | 0.0 | 1.0 | 0.0% |  |  |  |
| Suspicion of NAI | Dunning | 2006 | <16 | 0.3% |  |  |  | 39.0 | 0.9 | 0.1% |  |  |  |
| Assault | Osmond | 2010 | < 17 | 0.5% |  |  |  | 6.2 | 1.0 | 0.1% |  |  |  |
| fight/assault | Simon | 2001 | <16 | 0.9% |  |  |  | 2.0 | 1.0 | 0.2% |  |  |  |
| Assaulted | Klassen | 2000 | <16 | 5.8% |  |  |  | 1.5 | 1.0 | 0.4% |  |  |  |

| **27. Struck by object/sports trauma** | | | |  |  |  |  |  |  |  |  |  |  |
| --- | --- | --- | --- | --- | --- | --- | --- | --- | --- | --- | --- | --- | --- |
| **Risk factor** | **Study** | **Year** | **Age** | **Prev risk factor** | **PLR CT** | **NLR CT** | **Prev CT** | **PLR ICI** | **NLR ICI** | **Prev ICI** | **PLR NS** | **NLR NS** | **Prev NS** |
| **If age = any** |  |  |  |  |  |  |  |  |  |  |  |  |  |
| Impact from objects | Guzel | 2009 | <17 | 6.4% | 1.2 | 1.0 | 0.55% |  |  |  |  |  |  |
| Struck by object | Bechtel | 2009 | < 18 | 9.9% |  |  |  | 1.3 | 1.0 | 2.0% |  |  |  |
| Head struck | Osmond | 2010 | < 17 | 3.2% |  |  |  | 2.3 | 1.0 | 0.3% |  |  |  |
| High-speed injury from projectile or object | Dunning | 2006 | <16 | 2.0% |  |  |  | 3.3 | 1.0 | 0.1% |  |  |  |
| Sports trauma | Klemetti | 2009 | <17 | 12.4% | 1.1 | 1.0 | 2.27% |  |  |  |  |  |  |
| Sports injury | Boran | 2006 | <17 | 5.7% |  |  |  | 0.0 | 1.1 | 0.0% |  |  |  |
| Sports | Simon | 2001 | <16 | 7.9% |  |  |  | 2.1 | 0.9 | 2.1% |  |  |  |
| Contact sports | Klassen | 2000 | <16 | 10.6% |  |  |  | 0.2 | 1.1 | 0.1% |  |  |  |
| Contact injury (?) | Atabaki | 2008 | <21 | 9.1% |  |  |  | 0.5 | 1.1 | 0.3% |  |  |  |
| Others | Klassen | 2000 | <16 | 19.4% |  |  |  | 0.2 | 1.2 | 0.2% |  |  |  |
| Other | Boran | 2006 | <17 | 7.4% |  |  |  | 0.0 | 1.1 | 0.0% |  |  |  |
| Other | Davis |  | 2-17 | 17.3% |  |  |  | 0.8 | 1.0 | 1.2% |  |  |  |
| Other trauma (what trauma?) | Klemetti | 2009 | <17 | 2.9% | 5.2 | 0.9 | 2.68% |  |  |  |  |  |  |
| Unkown trauma | Klemetti | 2009 | <17 | 0.4% | 0.0 | 1.0 | 0.00% |  |  |  |  |  |  |
| multiple visits | Ramundo | 1995 | 2-18 | 10.7% | 1.3 | 1.0 | 2.30% |  |  |  |  |  |  |
| unsepcified mechanism | Ramundo | 1995 | 2-18 | 23.4% | 0.9 | 1.0 | 3.83% |  |  |  |  |  |  |
| multiple visits | Ramundo | 1995 | <2 | 21.6% | 1.2 | 0.9 | 5.41% |  |  |  |  |  |  |
| unsepcified mechanism | Ramundo | 1995 | <2 | 10.8% | 3.6 | 0.8 | 5.41% |  |  |  |  |  |  |

| **28. Coagulopathy** | | | |  |  |  |  |  |  |  |  |  |  |
| --- | --- | --- | --- | --- | --- | --- | --- | --- | --- | --- | --- | --- | --- |
| **Risk factor** | **Study** | **Year** | **Age** | **Prev risk factor** | **PLR CT** | **NLR CT** | **Prev CT** | **PLR ICI** | **NLR ICI** | **Prev ICI** | **PLR NS** | **NLR NS** | **Prev NS** |
| Coagulopathy | Fabbri | 2011 | < 11 | 0.04% |  |  |  | 0.0 | 1.0 | 0.0% |  |  |  |
| Coagulopathy | Oman | 2006 | 0-18 | 1.5% |  |  |  | 7.0 | 0.9 | 0.5% |  |  |  |
| Coagulopathy | Oman | 2006 | 0-3 | 27.4% |  |  |  | 0.7 | 0.1 | 0.3% |  |  |  |

| **29. Shunts** | | | |  |  |  |  |  |  |  |  |  |  |
| --- | --- | --- | --- | --- | --- | --- | --- | --- | --- | --- | --- | --- | --- |
| **Risk factor** | **Study** | **Year** | **Age** | **Prev risk factor** | **PLR CT** | **NLR CT** | **Prev CT** | **PLR ICI** | **NLR ICI** | **Prev ICI** | **PLR NS** | **NLR NS** | **Prev NS** |
| ventricular shunts | Nigrovic | 2013 | < 18 | 0.3% |  |  |  | 1.2 | 1.0 | 0.0% |  |  |  |

| **30. S100B** | | | |  |  |  |  |  |  |  |  |  |  |
| --- | --- | --- | --- | --- | --- | --- | --- | --- | --- | --- | --- | --- | --- |
| **Risk factor** | **Study** | **Year** | **Age** | **Cut-off**  **(µg/l)** | **Sensitivity** | **Specificity** |  | **PLR CT** | **NLR CT** | **PLR ICI** | **NLR ICI** |  |  |
| S100B | Hallén | 2010 | <18 | 0.195 | 1.0 | 0.88 |  | 8.3 | 0.0 |  |  |  |  |
| S100B | Bechtel | 2009 | <18 | 0.05 | 0.75 | 0.56 |  |  |  | 1.7 | 0.5 |  |  |
| S100B | Bouvier | 2012 | <17 | According to age reference | 1 | 0.33 |  | 1.5 | 0.0 |  |  |  |  |
| S100B | Castellani | 2010 | <18 | 0.16 | 1 | 0.42 |  | 1.7 | 0.0 |  |  |  |  |
