# Supplementary material for: Scandinavian guidelines for initial management of minor and moderate head trauma in children
Source: BMC Med. 2016 Feb 18;14:33. doi: 10.1186/s12916-016-0574-x (PMC4758024; doi:10.1186/s12916-016-0574-x)
Supplement: Additional file 6: Table S6. — GRADE for predictive factors of interest for CT findings, intracranial injury, and neurosurgery. (DOCX 26 kb) [file 12916_2016_574_MOESM6_ESM.docx]

| **Predictive factors** | **Age** | **Study No** | **No of studies (ICI/CT/NS)** | **Initial quality of evidence** | **Bias** | **Indirectness** | **Inconsistency** | **Imprecision** | **Publication bias** | **Large effect** | **GRADE** | | |
| --- | --- | --- | --- | --- | --- | --- | --- | --- | --- | --- | --- | --- | --- |
|  |  |  |  |  |  |  |  |  |  |  | **ICI** | **CT** | **NS** |
| Moderate Head injury | Any | 5, 9, 14, 15, 16, 18, 19, 25, 36, 39, 45, 46, 47, 50 | 10/4 | Moderate | -1 | -1 | -1/0 | 0 | 0 | 1/0 | Very low | Very low |  |
| Focal neurology | < 4 y | 19,30, 33, 36, 40 | 3/2 | High | -2 | -1 | -1 | -1 | 0 | 1 | Very low |  |  |
|  | Any | 5, 6, 8,11, 13, 15, 16, 18, 19, 22, 30, 31,32, 33,36, 37, 38, 40, 42 | 10/11/(2) | High | -1 | -1 | -2 | -1 | 0 | 2 | Very low | Very low | Very low |
| Seizures (unspecified) | < 4 y | 19, 29 | 2 | High | -1 | -2 | -1 | -2 | -1 | 0 | Very low |  |  |
|  | Any | 5, 6, 9,13, 15,17,18, 19, 20, 22, 23, 29, 30, 32, 33, 35, 36, 37, 38, 40, 41, 42, 43 | 17/6 | High | -1 | -1 | -2 | -1 | 0 | 1 | Very low |  |  |
| Sign of skull fracture or skull base fracture | < 4 y | 12,19,33,36 | 3/1 | High | -2 | -1 | -1 | -1 | 0 | 1 | Very low |  |  |
|  | Any | 6, 9, 11,12, 13, 15, 17, 18, 19, 22, 24, 32, 36, 38, 39, 42 | 12/4 | High | -2 | -1 | -2 | -1 | 0 | 1 | Very low |  |  |
| GCS score 14 + 13-14 vs 15 | Any | 9,12,13,14,15, 21,24,27, 39, 43, 45,46 | 8 /4 | High | -1 | -1 | -1 | 0 | 0 | 0 | Very low |  |  |
| Altered mental status  (GCS score 14, drowsiness, sleepiness, altered or depressed consciousness/alertness) | < 4 y | 19,12,29, 30,33 | 5 | High | -1 | -1 | -1 | 0 | 0 | 0 | Very low |  |  |
|  | Any | 6, 9, 12,15,17,18,19,21,22, 24, 30,32, 33,39, 41, 45, 11, 43,46 | 15/4 | High | -1 | -1 | -1 | 0 | 0 | 0 | Very low |  |  |
| LOC ≥ 1min | Any | 5,12,9,19,16,18,32, 25, 36, 37 | 8/2 | High | -1 | 0 | -1 | -1 | 0 | 1 | Low |  |  |
| Coagulopathy | Any | 6,19 | 2 | Moderate | -1 | 0 | -1 | -2 | -1 | 0 | Very low |  |  |
| Post traumatic amnesia | Any | 13,16 | 1/1 | High | -1 | -1 | 0 | -1 | -1 | 1 | Very low |  |  |
| Amnesia > 5min | Any | 18 | 1 | High | 0 | -1 | 0 | -1 | -1 | 2 | Moderate |  |  |
| LOC unspecified | < 4 y | 30, 36, 40, 12, 19,33,29 | 5/2 | High | -1 | -1 | -1 | 0 | 0 | 0 | Very low |  |  |
|  | Any | 5, 6, 8, 9, 10, 11, 12, 13, 15, 17, 19, 20, 22, 24, 27, 28, 29, 30, 33, 35, 36, 37, 38, 40, 42, 43, 50 | 19/8 | High | -1 | -2 | -2 | -1 | 0 | 0 | Very low |  |  |
| Moderate-severe or progressive headache | Any | 12,16,19,32 | 4 | High | -1 | -1 | -2 | -1 | 0 | 0 | Very low |  |  |
| Abnormal behaviour according to guardian | Any | 6,11,12,19,30 | 4/1 | High | -1 | -1 | -1 | 0 | 0 | 0 | Very low |  |  |
| Irritability | < 2 | 29, 35 | 2/0 | Moderate | -1 | -1 | -1 | 0 | -1 | 0 | Very low |  |  |
| Scalp hematoma defined as significant or large | < 2 y | 29,26 | 2 | High | -2 | -1 | -1 | -1 | -1 | 0 | Very low |  |  |
|  | Any | 9, 26,29 | 3 | High | -1 | -1 | -1 | -1 | 0 | 0 | Very low |  |  |
| Scalp hematoma, location | < 2 y | 12, 26 | 2 | High | -1 | -1 | -1 | -1 | 0 | 0 | Very low |  |  |
| Vomiting, repetitive | Any | 6, 9, 12, 17, 18, 19, 50 | 7 | High | 0 | -1 | -1 | -1 | 0 | 0 | Very low |  |  |
| Vomiting, any | Any | 2,5, 8, 10, 11, 12, 13, 15, 16, 17, 22, 23, 29, 30, 32, 33, 35, 36, 37, 38, 40 41, 42, 43 | 16 /9 | High | -1 | -1 | -1 | -1 | 0 | 1 | Very low | Very low | Very low |
| Deterioration | Any | 9, 17,50 | 3 | High | -1 | -1 | -1 | -1 | -1 | 2 | Very low |  |  |
| Bulging fontanel | < 2 y | 29 | 1 | High | -2 | -1 | -1 | -1 | -2 | 2 | Very low |  |  |
| Traffic accident (Motor vehicle involvement) | Any | 9, 10, 11,13,15, 18, 20, 24, 27, 36, 37, 43 | 8/4 | High | -1 | -1 | -1 | 0 | 0 | 1 | Low |  |  |

Additional file 6, Table S6: GRADE for predictive factors of interest for CT findings, intracranial injury and neurosurgery

GRADE (Grading of Recommendations Assessment, Development and Evaluation) for rating the quality of evidence and strength of recommendations. ICI = intracranial injury, CT = computed tomography, NS = neurosurgery (any neurosurgical procedure). GCS = Glasgow Coma Scale, LOC = Loss of consciousness. Study numbers refer to the study numbers given in Table S1; the evidentiary table for the clinical question 1
