# Supplementary material for: Scandinavian guidelines for initial management of minor and moderate head trauma in children
Source: BMC Med. 2016 Feb 18;14:33. doi: 10.1186/s12916-016-0574-x (PMC4758024; doi:10.1186/s12916-016-0574-x)
Supplement: Additional file 7: Table S7. — Results of the modified Delphi process, round 1. Delphi point 1 = strongly disagree, Delphi point 7 = strongly agree. Two members did not reply. Delphi points 1-5 refer to recommendations concerning clinical question 1. Delphi points 6-7 refer to the recommendations regarding clinical question 2. Point 8 refers to the written discharge instructions, point 9 to the observation schedule for in-hospital observation, and point 10 refers to the guideline draft including the guideline flow-chart. Result refers to percentage in favour of the recommendations. Cf = consensus for, nC = no consensus, Ca = consensus against. (DOCX 20 kb) [file 12916_2016_574_MOESM7_ESM.docx]

Additional file 7 Table S7: Results of the modified Delphi process, round 1

| Delphi point | Working group member/Stake holder | | | | | | | | | | | | | | | | |  |  | Result | Cf/nC/ Ca |
| --- | --- | --- | --- | --- | --- | --- | --- | --- | --- | --- | --- | --- | --- | --- | --- | --- | --- | --- | --- | --- | --- |
|  | **1** | **2** | **3** | **4** | **5** | **6** | **7** | **8** | **9** | **10** | **11** | **12** | **13** | **14** | **15** | **16** | **17** | **18** | **19** |  |  |
| 1 | 6 | 6 | 6 | 7 | 7 | 7 | 5 | 7 | 5 | 6 | 7 | 7 | 7 | 6 | 7 | 7 | - |  |  | 100% | Cf |
| 2 | 7 | 7 | 7 | 7 | 7 | 6 | 5 | 7 | 7 | 6 | 7 | 7 | 7 | 7 | 7 | 7 | - |  |  | 100% | Cf |
| 3 | 7 | 3 | 5 | 7 | 7 | 7 | 4 | 7 | 7 | 6 | 7 | 7 | 7 | 6 | 7 | 7 | - |  |  | 88% | Cf |
| 4 | 7 | 4 | 4 | 3 | 7 | 3 | 3 | 6 | 7 | 6 | 5 | 7 | - | 6 | 7 | 7 | - |  |  | 67% | nC |
| 5 | 7 | 2 | 4 | 3 | 7 | 5 | 3 | 5 | 6 | 5 | 4 | 7 | 6 | 7 | 7 | 7 | - |  |  | 69% | nC |
| 6 | 7 | 6 | 7 | 7 | 7 | 6 | 4 | 7 | 7 | 7 | 7 | 7 | 7 | 7 | 7 | 7 | - |  |  | 94% | Cf |
| 7 | 7 | 4 | 7 | 7 | 7 | 7 | 6 | 7 | 6 | 7 | 7 | 1 | 7 | - | 7 | 7 | - |  |  | 87% | Cf |
| 8 | 5 | 6 | 5 | 2 | 7 | 3 | 5 | 7 | 5 | 2 | 6 | 6 | 7 | 5 | 7 | - | - |  |  | 80% | Cf |
| 9 | 7 | 3 | 5 | 4 | 7 | 5 | 6 | 7 | 6 | 2 | 7 | 7 | 7 | 5 | 7 | 7 | - |  |  | 88% | Cf |
| 10 | - | 5 | 5 | 6 | 7 | 6 | 5 | 6 | 5 | 6 | 7 | 5 | 7 | - | 5 | - | 5 |  |  | 100% | Cf |

Delphi point 1 = strongly disagree, Delphi point 7 = strongly agree. Two members did not reply. Delphi points 1-5 refers to recommendations concerning the clinical question 1. Delphi points 6-7 refers to the recommendations regarding the clinical question 2. Point 8 refers to the written discharge instructions, point 9 to the observation schedule for in-hospital observation, and point 10 refers to the guideline draft including the guideline flow-chart. Result refers to percentage in favour of the recommendations. Cf = consensus for, nC = no consensus, Ca = consensus against.
