# Supplementary material for: Scandinavian guidelines for initial management of minor and moderate head trauma in children
Source: BMC Med. 2016 Feb 18;14:33. doi: 10.1186/s12916-016-0574-x (PMC4758024; doi:10.1186/s12916-016-0574-x)
Supplement: Additional file 9: Table S9. — Results of the modified Delphi process, round 3. Delphi point 1 = strongly disagree, Delphi point 7 = strongly agree. Delphi point 7 refers to the revised recommendation regarding discharge after a normal CT, clinical question 2. Delphi points 8 and 9 refer to the finalized written discharge advice and in-hospital monitoring instructions. Result refers to percentage in favour of the recommendations. Cf = consensus for, nC = no consensus, Ca = consensus against. (DOCX 19 kb) [file 12916_2016_574_MOESM9_ESM.docx]

Additional file 9 Table S9: Results of the modified Delphi process, round 3

| Delphi point | Working group member/Stake holder | | | | | | | | | | | | | | | | | | | Result | Cf/nC/ Ca |
| --- | --- | --- | --- | --- | --- | --- | --- | --- | --- | --- | --- | --- | --- | --- | --- | --- | --- | --- | --- | --- | --- |
|  | **1** | **2** | **3** | **4** | **5** | **6** | **7** | **8** | **9** | **10** | **11** | **12** | **13** | **14** | **15** | **16** | **17** | **18** | **19** |  |  |
| 7 | 5 | 6 | 7 | 7 | 7 | 7 | 7 | 6 | 7 | 7 | 7 | 7 | 6 | 7 | 6 | 6 | 6 | 7 | 5 | 100% | Cf |
| 8 | 7 | 6 | 7 | 6 | 7 | 7 | 7 | 7 | 6 | 7 | 6 | 7 | 6 | 6 | 7 | 6 | 5 | 7 | 5 | 100% | Cf |
| 9 | 7 | 6 | 5 | 5 | 7 | 6 | 7 | 6 | 6 | 7 | 6 | 7 | 6 | 6 | 7 | 6 | 6 | 7 | 6 | 100% | Cf |

Delphi point 1 = strongly disagree, Delphi point 7 = strongly agree. Delphi point 7 refers to the revised recommendation regarding discharge after a normal CT, clinical question 2. Delphi point 8 and 9 refer to the finalized written discharge advises and in-hospital monitoring instructions. Result refers to percentage in favour of the recommendations. Cf = consensus for, nC = no consensus, Ca = consensus against.
