# Supplementary material for: Scandinavian guidelines for initial management of minor and moderate head trauma in children
Source: BMC Med. 2016 Feb 18;14:33. doi: 10.1186/s12916-016-0574-x (PMC4758024; doi:10.1186/s12916-016-0574-x)
Supplement: Additional file 10: — Help sheet for the Scandinavian guidelines for initial management of minor and moderate head trauma in children. (DOCX 24 kb) [file 12916_2016_574_MOESM10_ESM.docx]

**Scandinavian guidelines for initial management of minor and moderate head trauma in children**

**General points**

This guideline is based upon evidence-derived recommendations and consensus aspects from the Scandinavian Neurotrauma Committee working group. Being a guideline, it should not override clinical judgement and may be super-seeded if necessary. GCS scores should be noted after resuscitation and a thorough clinical examination of the child should be performed so that the various risk factors may be adequately assessed. The paediatric GCS can be used for non-verbal children, <5 years of age. The guidelines apply to all children (< 18 years of age) who have sustained a head trauma within the last 24 hours.

Head trauma is considered to be any blow or hit towards the head; which is not necessarily equivalent to the child having a brain injury. The classification of head trauma severity is based on a modification of the Head Injury Severity scale, similar to the revised Scandinavian head injury guidelines for adults (2013).

Some children need extra attention apart from these guidelines, as noted in the NB!-box. These are patients with high-velocity trauma mechanisms who should be admitted and CT scanned according to national/local trauma protocols. Patients with suspected non-accidental injury (NAI) should be managed according to national protocols for suspected NAI. Children younger than 1 year of age may be difficult to assess clinically, thus admitting the child for in-hospital observation after a head trauma may be an option regardless of the child being asymptomatic. A bulging fontanel in an infant (examined when the child is not crying) can be a sign of increased intracranial pressure, which is usually also accompanied with a decreased level of consciousness or other neurological deficits. These patients should be admitted to the hospital and CT scanned. The flow chart is read from top to bottom and left to right.

**Moderate**

These children have an initial GCS score of 9-13, and should have a CT and also be admitted for close observation for at least 24 hours, even if the CT is normal.

**Mild, high-risk**

These children have a GCS score of 14-15 and have either clinical signs of depressed skull fracture (palpable depression or abnormal discontinuity of skull) or basal skull fracture (raccoon eyes, Battle’s sign, hemotympanum, cerebrospinal fluid rhinorrhea or otorrhea), have focal neurological deficits (function loss from a specific part of the body, e.g. motor loss in the face, arm or leg, cranial nerve affection or sudden speech disorder, suggestive of cerebral dysfunction), or have had a post-traumatic seizure. These patients are relatively uncommon but they have a relatively high risk of intracranial complications. They should have a CT and also be admitted for close observation for at least 24 hours, even if the CT is normal.

**Mild, medium-risk**

These children have (a) a GCS score of 14 or (b) a GCS score of 15 and have had a loss of consciousness (LOC) for an estimated time of 1 minute or more, or have a known coagulation disorder (such as haemophilia, thrombocytopenia or liver disorders with pathological INR (>1.4)) or are taking anticoagulation medication (coumadin/warfarin, low molecular-weight heparins or other pharmacologic anticoagulation). These patients have a moderate risk of intracranial complication and should be admitted for observation for at least 12 hours (from time of trauma). Alternatively, the child could have a CT scan and if normal, can be discharged with close follow-up.

**Mild, low-risk**

These children have a GCS score of 15 and have suspected or confirmed brief LOC (i.e. you cannot clearly exclude loss of consciousness), confirmed post-traumatic amnesia, repeated vomiting (at least two episodes), severe or progressive headache, have a ventricular shunt, or are acting abnormally according to their guardians. Children < 2 years old with a GCS score 15 and presenting with a large scalp haematoma or a haematoma in the temporal or parietal region or if the child seems irritable to normal touch or stimulus, is also classed as mild, low-risk. These children should be clinically observed for at least 6 hours (from the time of trauma) until the symptoms have resolved (e.g. headache or vomiting) and the child is fully awake and stable without signs of deterioration. If multiple risk factors are present, consider doing a CT scan instead.

**Minimal**

These patients have a GCS score of 15 and have none of the risk factors mentioned in the other boxes. These patients have a very low risk of serious intracranial complication and can be discharged from the emergency department without a CT scan.

---------------------------------------------------------------------------------------------------------------------------------------

**Admission and observation**

CT is recommended as the primary radiological routine. Patients with moderate and mild, high-risk head trauma should be admitted irrespective of CT findings for at least 24 hours. Recommended minimum observation time is measured from the time of trauma. Observation should include the level of consciousness (GCS score), a simplified neurological examination (strength testing in extremities, language and speech) and pulse rate, but may be further extended to assessment of headache intensity and pupil size and reactivity especially in children with decreased level of consciousness. Monitoring these symptoms should be performed ***every 15 minutes*** for the first 4 hours after trauma, ***every 30 minutes*** for the following 4 hours and ***at least every hour*** hereafter.

Some with minimal head trauma or normal CT following mild head trauma may need admission for reasons other than the head trauma (e.g. children with insufficient guardian supervision, children with other injuries or heavily intoxicated adolescents). Since these patients have a very low risk of intracranial injury, they need to be checked at least every 4 hours, but do not need the extensive observation routine mentioned above.

**CT/repeat CT**

A non-contrast CT should be done as quickly as possible, with a greater urgency for moderate and mild, high-risk head trauma. The child should be adequately monitored when waiting for the CT scan. When CT is abnormal, consider contacting a neurosurgeon or neurotrauma centre for advice concerning further management. Repeat CT should be done immediately in patients with deterioration in GCS (≥2 points) or new/progressive neurological deficits.

**Discharge**

Guardians to ALL children with head trauma should receive oral and written instructions at discharge for general information and contact information. (See separate forms: written instructions to guardians after early discharge, and written instructions to guardians after in-hospital/short-term observation.)
