# Supplementary material for: Scandinavian guidelines for initial management of minor and moderate head trauma in children
Source: BMC Med. 2016 Feb 18;14:33. doi: 10.1186/s12916-016-0574-x (PMC4758024; doi:10.1186/s12916-016-0574-x)
Supplement: Additional file 11: — Early discharge advice for the guardians of a child who has sustained a minor head trauma. (DOCX 26 kb) [file 12916_2016_574_MOESM11_ESM.docx]

**Discharge advice for the guardians of a child who has sustained a minor head trauma**

A doctor has examined your child after the head trauma, and based on this medical evaluation we consider it safe to take your child with you for observation at home. You should not leave the child alone for the first 24 hours after the time of head trauma.

**Things you need to know**

It is acceptable for the child to sleep, but you should check your child every 4 hours the first night in order to see that the child is all right.

- Does he or she seem to be breathing normally? Is he or she sleeping in a normal posture? Does he or she react as expected to gentle touch?

If you suspect or are in doubt that your child is not sleeping normally, he/she should be awakened fully to be checked.

It is acceptable to take mild pain killers, e.g. paracetamol, for the first few days in dosages recommended by your doctor.

Alcohol is strongly prohibited and adolescents should not be allowed to drink alcohol as this may worsen the symptoms.

Sports and physical exercise/activities should not be resumed before your child is without symptoms at rest, and it should be gradually increased over a period of 1 week. If symptoms such as mild headache, dizziness, memory problems or sleep disturbances occur, the child should have a 24 hour rest from the physical exercise before resuming the gradual increase in physical activities again.

**Things you should not worry about**

It is quite normal to have some mild symptoms for approximately 2 weeks after a head trauma. This includes being sleepier, having trouble sleeping, having mild headache, light nausea and having trouble concentrating. If any of these symptoms worry you or your child is not back to normal after a couple of weeks, you should contact your local paediatric outpatient clinic or your family doctor in order to evaluate these problems.

**What to look out for**

Contact emergency medical service or your hospital (see contact information below) if any alarming symptoms occur, such as worsening symptoms, severe headache, repeated vomiting, altered behaviour or confusion, drowsiness, seizures, weakness of one or more limbs or watery fluid or blood running from ear, nose or mouth.
