# Supplementary material for: Scandinavian guidelines for initial management of minor and moderate head trauma in children
Source: BMC Med. 2016 Feb 18;14:33. doi: 10.1186/s12916-016-0574-x (PMC4758024; doi:10.1186/s12916-016-0574-x)
Supplement: Additional file 12: — Discharge advice for the guardians of a child who has sustained a mild head trauma or concussion. (DOCX 23 kb) [file 12916_2016_574_MOESM12_ESM.docx]

**Discharge advice for the guardians of a child who has sustained a mild head trauma or concussion**

**What is a mild head trauma or concussion?**

A concussion or mild head trauma is common especially in children. They are a result of e.g. a bump or blow to the head. It is unusual for there to be any significant damage to the brain, and most children with mild head trauma or concussion recover quickly and fully.

A doctor has examined your child after the head trauma; the symptoms have been checked and the child seems to be well on the road to recovery. It is now safe to take your child home. It is very unlikely that the child will have any further problems, but there is some information you need to be aware of.

**Things you need to know**

Rest after mild head trauma/concussion is important for recovery. Physical activity and cognitive loads (computer games, school work) can delay the recovery after a concussion. It is acceptable that the child may miss 1-2 days of school, but extended absence is usually not necessary.

The child should not resume sports activities before he or she has been able to return to school without worsening symptoms; hence, sports and physical activities should not be resumed before your child is without symptoms at rest. These activities should then be gradually increased over a period of 1 week. If symptoms such as e.g., mild headache, dizziness, memory problems or sleep disruption occur, a 24 hour rest from physical exercise should be done before resuming the gradual increase in physical activities again.

If your child has been unconscious or has significant memory loss after head trauma, there should be no full physical contact activity (such as ice hockey, football etc.) within 3 weeks after trauma. Sports should be increased gradually after that over a period of 1 week.

It is acceptable to take mild pain killers, such as e.g. paracetamol for the first few days in dosages recommended by your doctor. Alcohol is strongly prohibited and adolescents should not be allowed to drink alcohol as this may worsen the symptoms.

**Things you should not worry about**

It is quite normal to have some mild symptoms for approximately 2 weeks after a head trauma. This includes being sleepier, having trouble sleeping, having mild headache, nausea, and having trouble concentrating. If any of these symptoms worry you or your child isn’t back to normal after a couple of weeks, you should contact your local paediatric outpatient clinic or see your family doctor in order to evaluate these problems.

**What to watch for**

Contact emergency medical service or your hospital (see contact information below) if any alarming symptoms occur, such as worsening of symptoms, severe headache, repeated vomiting, altered behaviour or increasing confusion, increasing drowsiness, seizures, weakness of one or more limbs or watery fluid or blood running from ear, nose or mouth.
