# Supplementary material for: Scandinavian guidelines for initial management of minor and moderate head trauma in children
Source: BMC Med. 2016 Feb 18;14:33. doi: 10.1186/s12916-016-0574-x (PMC4758024; doi:10.1186/s12916-016-0574-x)
Supplement: Additional file 13: — Observation schedule on admission for children (<18 years) after mild and moderate head trauma. (DOCX 22 kb) [file 12916_2016_574_MOESM13_ESM.docx]

**Observation schedule on admission for children (< 18 years) after mild and moderate head trauma**

**Length of observation:** Paediatric patients with mild, low-risk head trauma should be admitted for > 6 hours, mild, medium risk head trauma for > 12 hours and mild, high-risk and moderate head trauma for > 24 hours.

**Observations:** It is important that admitted children not be left unattended (continuous parental observation can be sufficient). This applies also in the emergency room. Rigorous control should be saved for symptomatic children. All observations should be scheduled and documented.

Asymptomatic, fully awake children (i.e. children behaving as normal) do not need frequent specific monitoring; once an hour is sufficient.

For other children, specific observations should be scheduled every 15 minutes for the first 4 hours *after the trauma*, followed by every 30 minutes for the next 4 hours, and at least every hour thereafter.

Observation should include:

- Level of consciousness according to Glasgow Coma Scale (GCS) score
- Simplified neurological examination (adequately moving extremities, normal language and speech)
- Pulse rate
- Children below 2 years of age: fontanel examination (normal or bulging)

Observation may include:

- Pupil size and, especially, pupil reactivity
- Headache intensity

**Increase attention and contact the physician in case of**

- Decrease in alertness or consciousness
- New or increasing intensity in symptoms/findings
- Visual disturbances or difference in pupil size or reaction
- Inconsolable crying
- Bulging fontanel (in child below 2 years of age and examined when the child is not crying)

**CT or repeat CT should be performed in case of clinical deterioration (e.g. drop in GCS ≥ 2 points or new/worsening neurological deficits).**

**Consider additional observation or CT if the patient has not normalised during the observation period.**
